# Calcium, zinc, and vitamin D in breast milk: a systematic review and meta-analysis

**DOI:** 10.1186/s13006-023-00564-2

**Published:** 2023-06-01

**Authors:** Magali Rios-Leyvraz, Qisi Yao

**Affiliations:** 1 Consultant, Department of Nutrition and Food Safety, World Health Organization, Geneva, Switzerland; 2 Department of Nutrition and Food Sciences, University of Rhode Island, Kingston, USA

**Keywords:** Breast milk, Calcium, Zinc, Vitamin D, 25OHD, Systematic review, Meta-analysis

## Abstract

**Background:**

Global estimates of calcium, zinc and vitamin D content in breastmilk are lacking. The objective of this systematic review was to determine the calcium, zinc, and vitamin D content in breast milk.

**Methods:**

A systematic search of the online databases Embase, MEDLINE, and CENTRAL was conducted in November 2022 and complemented by searches of the African Journals Online database and the LILACS database, and reference lists. Studies reporting the calcium, zinc and vitamin D content in breast milk of apparently healthy mothers and infants were included. Random effects meta-analyses were conducted. The effect of influencing factors were investigated with sub-group analyses and meta-regressions.

**Results:**

A total of 154 studies reporting on breast milk calcium were identified, with a mean calcium concentration in breast milk of 261 mg/L (95% CI: 238, 284). Calcium concentration was influenced by maternal health and decreased linearly over the duration of lactation. Calcium concentration at a specific time during lactation could be estimated with the equation: calcium concentration [mg/L] = 282 – 0.2331 ✕ number of days since birth. A total of 242 studies reporting on breast milk zinc were identified, with a mean zinc concentration of 2.57 mg/L (95% CI: 2.50, 2.65). Zinc concentration was influenced by several factors, such as maternal age, gestational age, and maternal diet. Zinc concentration started high in the first weeks post-partum followed by a rapid decrease over the first months. Zinc concentration at a specific time during lactation could be estimated with the equation: zinc concentration [mg/L] = 6 + 0.0005 ✕ days – 2.0266 ✕ log(days). A total of 43 studies reporting on breast milk vitamin D were identified, with a mean total antirachitic activity of breast milk of 58 IU/L (95% CI: 45, 70), which consisted mostly of 25OHD3, and smaller amounts of vitamin D3, 25OHD2 and vitamin D2. Vitamin D concentration showed wide variations between studies and was influenced by vitamin D supplementation, continent and season.

**Conclusions:**

This review provides global estimates of calcium, zinc and vitamin D content in breast milk, as well as indications on changes over time and depending on influencing factors.

**Supplementary Information:**

The online version contains supplementary material available at 10.1186/s13006-023-00564-2.

## Background

Breast milk is a major component of the diet and an important source of nutrient intake in infants and young children [1, 2]. Exclusive breastfeeding up to 6 months of age is recommended, followed by continued breastfeeding up to two years or beyond [1, 2]. Breast milk from healthy well-nourished women is expected to provide adequate amounts and concentrations of the majority of nutrients for optimal growth of infants [1]. Based on this assumption, breast milk content in most nutrients can be used to estimate requirements in infants up to 6 months of age and children up to 3 years of age.

Calcium, zinc and vitamin D are essential nutrients during infancy and early childhood for growth and health [3–5]. Breast milk is a source of calcium and zinc and, in small amounts, of vitamin D for infants and young children. A multi-national study from the World Health Organization (WHO) provided estimates of calcium and zinc in breastmilk in 1989 [6]. Since then, many new studies have evaluated breast milk content in calcium and zinc as well as vitamin D. Updated reference values on calcium, zinc and vitamin D content in breast milk are needed.

This review was commissioned by the Food and Agriculture Organization (FAO) and WHO, to inform their work on updating nutrient requirements and safe upper levels of intake for calcium, zinc and vitamin D in infants and young children, originally established in 2004. The primary objective of this systematic review was to determine the calcium, zinc, and vitamin D content of breast milk. The secondary objective was to investigate the factors influencing the calcium, zinc, and vitamin D levels in breast milk.

## Methods

The preparation of the review protocol followed Preferred Reporting Items for Systematic reviews and Meta-Analyses Protocols (PRISMA-P) guidelines and is available upon request [7, 8]. The writing of this report followed the Preferred Reporting Items for Systematic reviews and Meta-Analyses (PRISMA) guidelines [9].

### Eligibility criteria

Studies conducted in apparently healthy lactating women and their offspring 0–35.9 months of age, free from any clinical signs or symptoms of undernutrition or illness that might impact milk composition and assessing the breast milk concentrations of calcium, zinc, and vitamin D (including vitamin D2, vitamin D3, 25-hydoxy-vitamin D (25OHD), 25OHD2, and 25OHD3) were included. Studies in which the measurement method, sample size, unit or standard deviation could not be determined were excluded. Cross-sectional, longitudinal, interventional, and case-control studies were included, but case reports and case series were excluded. Conference abstracts, posters, commentaries, editorials and studies for which the full texts were unobtainable were excluded. Studies from all regions of the world, in all languages, and of any date of publication were included.

### Search strategy

The online databases MEDLINE, MEDLINE In-Process & Other Non-Indexed Citations, EMBASE, and Cochrane Central Register of Controlled Trials (CENTRAL) were searched systematically up to 1 October 2020 originally and then again on 22 November 2022 (see full search strategy in **Additional File 1**). The African Journals Online database and the Literatura Latino-Americana e do Caribe em Ciências da Saúde (LILACS) database were searched to find additional studies from these regions. The reference lists of recent systematic reviews and included reports were screened manually to identify further potentially relevant studies.

### Selection process

The identified records were imported into Covidence [8, 10] and duplicates were identified automatically. The records were screened for eligibility in duplicate by two researchers (except for the records identified in the update, which were screened by only one reviewer). Any disagreement was resolved by discussion between the two reviewers.

### Data extraction

Information on the characteristics of the study, mother, child, and milk, as well as the measurement methods was extracted. If data were only available from figures, they were extracted with PlotDigitizer [11]. For trials, data were generally extracted from baseline and from the control group. If the intervention was relevant to sub-group analyses or if there were no significant effect of the intervention, data from baseline and endline and from the intervention and the control group were extracted. If several values for different milk processing steps were reported, the value for minimal processing was extracted. Data were extracted by one researcher and a subset was verified by another researcher.

### Data analysis

Data transformations and imputations were done according to the Cochrane Handbook for Systematic Reviews of Interventions [12] and following the recommendations of Borenstein et al. [13]. If means and SD were not reported, they were imputed from 95% CI, p-values, t-values, medians, percentiles, interquartile ranges, or ranges [12]. If values for several groups were reported, they were merged together [12]. Calcium, zinc, and vitamin D were transformed into a common unit (i.e. mg/L and IU/L) using the following conversions: calcium 40.078 g/mol, zinc 68.38 g/mol, vitamin D2 396.6 g/mol, vitamin D3 384.6 g/mol, 25OHD2 412.6 g/mol, 25OHD3 400.64 g/mol, vitamin D2 and vitamin D3 25ng/IU, and 25OHD2 and 25OHD3 5 ng/IU. Whenever available, vitamin D2, D3, 25OHD2 and 25OHD3 in IU/L, were added together to calculate total vitamin D and 25OHD, and total antirachitic activity (ARA). When non-detectable levels of vitamin D in breast milk were reported, the midpoint between 0 and the detection limit was taken and the standard deviation was set so that the upper 95% confidence interval lay at the detection limit.

Random effects meta-analyses were conducted. Heterogeneity was evaluated with I^2^ and τ^2^. Outlying and influential studies were identified with Baujat plots. Sub-group analyses were conducted by lactation stage (colostrum 1–4 days postpartum, transitional milk 5–15 days and mature milk > 15 days), health status, maternal age, gestation duration, supplementation in the micronutrient of interest, nutrition status, breastfeeding practice, country income category, continent, and measurement method. In addition for vitamin D, sub-group analyses by season and for different supplementation levels were conducted. Meta-regressions were conducted for calcium and zinc over lactation duration. Different meta-regression models, linear and non-linear (i.e. quadratic, cubic, logarithmic, exponential and restricted cubic splines), were tested and the best fitting model was selected based on AIC. Moreover, possible changes in breast milk concentrations of calcium, zinc and ARA over the years were investigated with linear meta-regressions. Meta-regressions of 25OHD, 25OHD2, 25OHD2, vitamin D, vitamin D2, vitamin D3, and ARA by latitude were conducted. A sensitivity analysis was conducted restricting to high quality studies, or ‘key’ studies. The studies were considered of high quality or ‘key’ if they included only healthy mothers and healthy term infants and, if the infants were below 6 months of age, exclusively breastfed.

Statistical analyses were conducted with RAnalyticFlow (version 3.1.8) with the package meta.

## Results

**Fig. 1:**
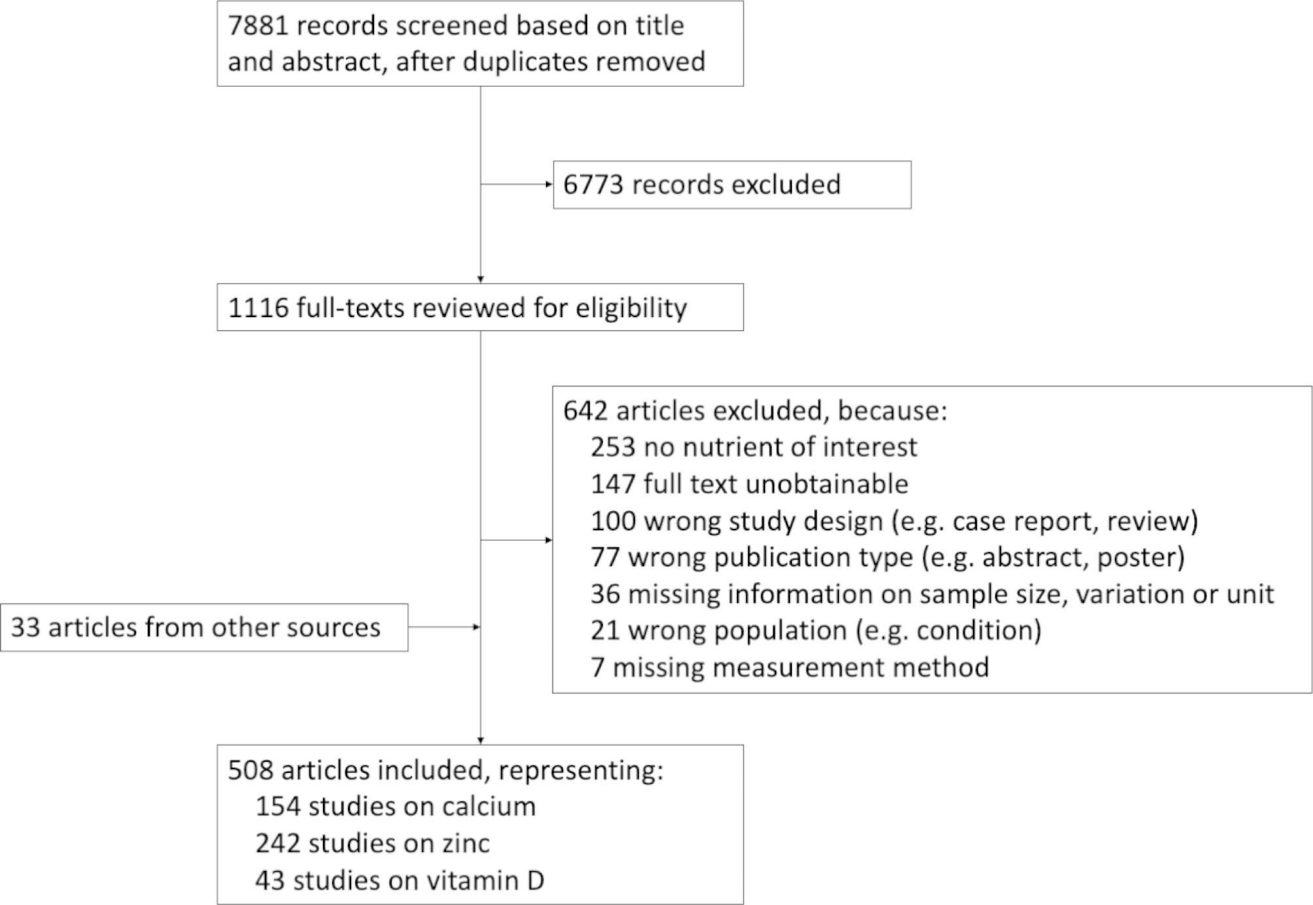
Study selection flowchart

From 7,881 records identified, a total of 507 records, representing 154 studies on calcium, 242 on zinc, and 43 on vitamin D, were included (see Fig. 1).

### Calcium

A total of 154 studies with 22,307 participants reporting on calcium concentration of breast milk were included. The detailed characteristics of the studies included are shown in **Additional File 2**. Among those studies, 76 were cross-sectional studies, 62 were cohorts, 8 were trials and 8 were case-control studies. The studies were conducted in Asia (N = 47), North America (N = 37), Europe (N = 33), South America (N = 22), Africa (N = 19) and Australasia/Oceania (N = 6). The countries in which the studies were the most conducted were the United States (N = 29), China (N = 18), and Brazil (N = 15). Studies were published between 1965 and 2022, with 25% published before 1988 and 25% published after 2014. Most of the studies included healthy participants (N = 83), but many did not report the health status of their participants (N = 63). Eight studies included both healthy and unhealthy participants. The health conditions included were in the mother, anemia (N = 1), COVID-19 (N = 1), diabetes type 1 (N = 1), gestosis (N = 1), mastitis (N = 2), and in the infant, small-for-gestational-age (N = 1) and rickets (N = 1). Most of the studies did not report the nutritional status of the mothers included (N = 134), a few reported good nutritional status (N = 12), poor nutritional status (N = 3) or both (N = 5). The analytical methods the most used to determine calcium in breast milk were atomic absorption spectroscopy (AAS) (N = 54), inductively coupled plasma mass spectrometry (ICP-MS) (N = 27), inductively coupled plasma atomic emission spectroscopy (ICP-AES) (N = 25), and flame atomic absorption spectroscopy (FAAS) (N = 17). The preferred method to measure calcium in breast milk is ICP-MS [14].

The results of the meta-analyses are shown in Table 1. The mean calcium concentration of breast milk was 261 mg/L (95% CI: 238, 284, range: 2, 686). One outlier with a very low value was identified, possibly due to the analytical method used. When removing this outlier, the mean calcium concentration was 262 mg/L (95% CI: 243, 282, range: 14, 686). When restricting the analysis to ‘key’ studies (i.e. studies with healthy women, healthy term infants, exclusively breastfed up to 6 months old), the mean calcium concentration was 249 mg/L (95% CI: 232, 266).

**Table 1:** Meta-analyses for calcium concentration (mg/L)

Group	N	n	Mean (95% CI)	p^a^	p^b^	
All studies	154	22,307	261 (238, 284)	NA	NA	
Studies without outlier	153	22,227	262 (243, 282)			
Key studies^c^	23	2766	249 (232, 266)			
**Infant age**						
0–5.9 months	121	14,999	270 (241, 300)	< 0.001	0.001	
6–11.9 months	32	2375	214 (163, 266)			
12–35.9 months	13	769	197 (177, 218)			
Unspecified	24	1881	252 (175, 329)			
**Infant age, key studies** ^c^						
0–5.9 months, healthy, term, exclusively breastfed	16	1386	271 (256, 286)	< 0.001	NA	
6–11.9 months, healthy, term	11	975	218 (189, 248)			
12–35.9 months, healthy, term	4	200	183 (162, 204)			
**Lactation stage**						
Colostrum	33	2024	269 (252, 286)	0.678	0.781	
Colostrum/transitional milk	11	293	281 (258, 304)			
Transitional milk	37	2257	270 (261, 279)			
Transitional/mature milk	7	402	232 (88, 376)			
Mature milk	120	15,513	257 (234, 280)			
Unspecified/Mixed	17	1774	265 (242, 288)			
**Maternal/infant health**						
Healthy	91	13,985	257 (232, 281)	0.008	0.012	
With condition	8	949	203 (171, 234)			
Unspecified/Mixed	63	7373	266 (227, 306)			
**Maternal age**						
Adults	78	11,909	268 (249, 286)	0.665	0.689	
Adolescents	5	337	254 (196, 313)			
Unspecified/Mixed	73	10,061	253 (224, 283)			
**Gestation**						
Term	66	7155	261 (217, 305)	0.867	0.940	
Preterm	19	1196	265 (249, 281)			
Unspecified/Mixed	85	13,929	260 (231, 288)			
**Breastfeeding practice**						
Exclusive	32	3132	260 (193, 328)	0.365	0.077	
Mixed	15	2056	228 (207, 249)			
Unspecified	115	17,158	264 (240, 288)			
**Calcium supplementation**						
Supplemented	5	571	251 (214, 289)	0.275	0.413	
Not supplemented	19	3742	275 (255, 294)			
Unspecified/Mixed	133	17,543	258 (234, 282)			
**Nutritional status**						
Good	14	2292	261 (243, 280)	0.082	0.178	
Poor	5	708	321 (256, 385)			
Unspecified/Mixed	138	19,342	256 (232, 280)			
**Country income category**						
Low	7	1280	282 (161, 403)	0.902	< 0.001	
Lower-middle	22	2259	246 (171, 321)			
Upper-middle	49	7436	257 (235, 279)			
High	85	9446	264 (256, 272)			
Unspecified/Mixed	1	1442	290 (287, 293)			
**Continent**						
Africa	19	2274	240 (198, 283)	0.618	NA	
Asia	47	8487	255 (233, 277)			
Australasia/Oceania	6	778	271 (217, 324)			
Europe	33	4108	270 (260, 280)			
North America	37	4334	261 (250, 272)			
South America	22	1882	270 (248, 292)			
**Measurement method**						
AAS	54	6964	263 (227, 299)	0.561	0.408	
FAAS	17	1922	277 (247, 307)			
ICP-AES	25	3957	272 (259, 286)			
ICP-MS^d^	27	5682	261 (250, 272)			
Other	31	3782	241 (207, 275)			

^a^ P-value for difference between groups without ‘Unspecified/Mixed’ group. ^b^ P-value for difference between groups for all groups. ^c^ Key studies include only studies conducted in healthy women, healthy and term infants, and exclusively breastfed if aged 0-5.9 months. ^d^ Method recommended for determination of calcium (14). NA: Not applicable, N: number of studies, n: number of participants

**Fig. 2:**
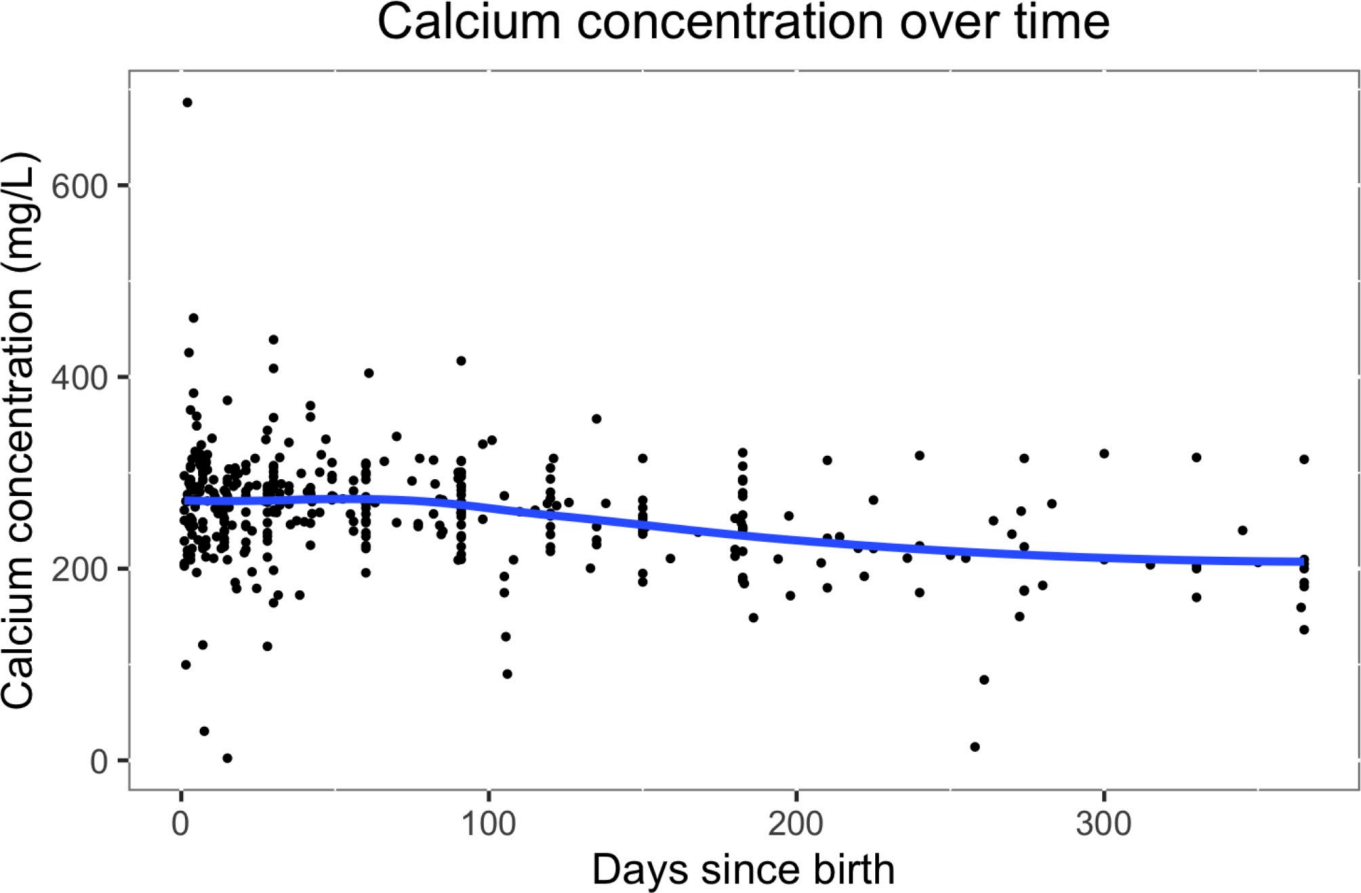
Calcium concentration (mg/L) over time, trendline (blue) fitted with local polynomial regression

Calcium concentration in breast milk changed with the infant’s age (see Table 1). The evolution of calcium concentration in breast milk over time is shown in Fig. 2. Calcium concentration was almost constant over time, with a very slow decrease. The evolution of calcium concentration over time was best modeled with a linear model. When including all studies across all ages, the calcium concentration [mg/L] at a certain timepoint could be estimated with the equation: 282.4357 – 0.2331 ✕ days. When restricting to ‘key’ studies, calcium concentration could be estimated with the equation: 276.7831 – 0.2169 ✕ days. When restricting to studies among 0-5.9 month-olds, the model was 285.5756 – 0.2554 ✕ days and, when restricting to ‘key’ studies and among 0-5.9 month-olds, 333.8875 – 0.7367 ✕ days.

There were no significant differences in calcium concentration between lactation stages, adolescent and adult mothers, preterm and term infants, exclusive and mixed breastfeeding, with or without calcium supplementation, between nutritional statuses, country income categories, continents, and measurement methods (see Table 1). However, women with conditions tended to have lower calcium breast milk concentrations than healthy women (women with conditions 203 mg/L, 95% CI: 171, 234, healthy women 257 mg/L, 95% CI: 232, 281, p = 0.008). Calcium concentration in breast milk did not differ significantly over the years of publication (p = 0.961).

The effect of maternal calcium intake on breast milk concentrations was further investigated. Two randomized controlled trials [15, 16] investigated the effect of calcium supplementation on calcium concentration in milk. When meta-analyzed, no significant differences were found between groups of calcium supplementation (mean difference (MD): 4.8 mg/L, 95% CI: -4.4, 14.1). One cohort study [17] compared women with different calcium intake levels and found no differences in calcium concentration in transitional milk, but a higher calcium concentration in women with higher calcium intakes in mature milk (p < 0.05). A cross-sectional study [18] found no differences between calcium intake groups and calcium concentration in mature breast milk.

### Zinc

A total of 242 studies with 37,614 participants reporting on zinc concentration of breast milk were included. The detailed characteristics of the studies included are shown in **Additional File 3**. Among those studies, 125 were cross-sectional studies, 92 were cohorts, 17 were trials and 8 were case-control studies. The studies were conducted in Asia (N = 84), Europe (N = 54), North America (N = 46), South America (N = 32), Africa (N = 32) and Australasia/Oceania (N = 5). The countries in which the studies were the most conducted were the United States (N = 37), Brazil (N = 24), and China (N = 22). Studies were published between 1971 and 2022, with 25% published before 1990 and 25% published after 2013. Most of the studies included healthy participants (N = 131), but many did not report the health status of their participants (N = 103). Eight studies included both healthy and unhealthy participants. The health conditions included were in the mother, acute febrile infection (N = 1), anemia (N = 1), diabetes type 1 (N = 1), HIV (N = 2), mastitis (N = 2), and in the infant, jaundice or intra-uterine growth restriction (N = 1). Most of the studies did not report the nutritional status of the mothers included (N = 206), a few reported good nutritional status (N = 24), poor nutritional status (N = 6) or both (N = 6). The analytical methods the most used to determine zinc in breast milk were AAS (N = 92), ICP-MS (N = 52), FAAS (N = 51), and ICP-AES (N = 35). The preferred methods to measure zinc in breast milk are AAS, ICP-AES, and ICP-MS [14, 19].

The results of the meta-analyses are shown in Table 2. The mean zinc concentration of breast milk was 2.57 mg/L (95% CI: 2.50, 2.65, range: 0.03–69.07). Three outliers and influential studies were identified, possibly due to errors in reported units. When removing them, the mean zinc concentration was not significantly different, with 2.58 mg/L (95% CI: 2.49, 2.67, range: 0.05–12.9). When restricting the analysis to ‘key’ studies (i.e. studies with healthy women, healthy term infants, exclusively breastfed up to 6 months old), the mean zinc concentration was 2.26 mg/L (95% CI: 2.00, 2.51).

**Table 2:** Meta-analyses for zinc concentration (mg/L)

Group	N	n	Mean (95% CI)	p^a^	p^b^	
All studies	243	37,614	2.57 (2.50, 2.65)	NA	NA	
All studies without outliers	240	37,266	2.58 (2.49, 2.67)			
Key studies^c^	31	4309	2.26 (2.00, 2.51)			
**Infant age**						
0–5.9 months	198	28,267	2.82 (2.73, 2.91)	< 0.001	< 0.001	
6–11.9 months	56	4280	1.18 (1.05, 1.32)			
12–35.9 months	12	411	0.76 (0.54, 0.98)			
Unspecified	27	2619	2.12 (1.80, 2.43)			
**Infant age, key studies** ^c^						
0–5.9 months, healthy, term, exclusively breastfed	25	3238	2.73 (2.44, 3.02)	< 0.001	NA	
6–11.9 months, healthy, term	17	1387	0.96 (0.79, 1.13)			
12–35.9 months, healthy, term	5	190	0.76 (0.30, 1.23)			
**Lactation stage**						
Colostrum	58	4689	6.39 (5.84, 6.95)	< 0.001	< 0.001	
Colostrum/transitional milk	24	1099	4.58 (3.43, 5.73)			
Transitional milk	61	3310	3.73 (3.51, 3.96)			
Transitional/mature milk	18	741	3.04 (1.62, 4.45)			
Mature milk	186	25,031	1.98 (1.88, 2.08)			
Unspecified/Mixed	25	2909	2.18 (1.48, 2.88)			
**Maternal/infant health**						
Healthy	137	23,192	2.58 (2.47, 2.69)	0.456	0.151	
With condition	9	1222	3.05 (1.81, 4.29)			
Unspecified/Mixed	104	13,450	2.76 (2.60, 2.91)			
**Maternal age**						
Adults	109	18,071	2.96 (2.75, 3.18)	< 0.001	< 0.001	
Adolescents	4	557	1.40 (1.28, 1.53)			
Unspecified/Mixed	131	18,856	2.57 (2.44, 2.69)			
**Gestation**						
Term	102	14,040	2.75 (2.60, 2.90)	0.280	0.045	
Preterm	26	2183	3.37 (2.26, 4.47)			
Unspecified/Mixed	137	21,537	2.53 (2.40, 2.67)			
**Breastfeeding practices**						
Exclusive	44	5636	2.58 (2.28, 2.87)	< 0.001	< 0.001	
Mixed	25	3894	1.45 (1.17, 1.74)			
Unspecified	178	27,885	2.84 (2.74, 2.95)			
**Zinc supplementation**						
Supplemented	13	1596	2.06 (1.72, 2.41)	0.012	0.018	
Not supplemented	48	7045	2.63 (2.36, 2.91)			
Unspecified/Mixed	195	28,795	2.57 (2.48, 2.65)			
**Nutritional status**						
Good	26	3996	3.22 (2.62, 3.83)	0.152	0.067	
Poor	9	1141	2.64 (2.13, 3.16)			
Unspecified/Mixed	211	32,762	2.51 (2.44, 2.59)			
**Country income category**						
Low	8	961	2.31 (1.72, 2.90)	< 0.001	< 0.001	
Lower-middle	44	5931	2.38 (2.21, 2.55)			
Upper-middle	83	12,706	2.92 (2.75, 3.09)			
High	116	15,867	2.49 (2.28, 2.70)			
Unspecified/Mixed	4	1960	3.04 (1.97, 4.10)			
**Continent**						
Africa	32	3931	3.03 (2.76, 3.3)	< 0.001	< 0.001	
Asia	84	15,477	2.78 (2.61, 2.95)			
Australasia/Oceania	5	579	1.75 (0.86, 2.64)			
Europe	54	7653	2.77 (2.48, 3.06)			
North America	46	6321	1.93 (1.7, 2.17)			
South America	32	3336	2.43 (2.27, 2.58)			
Unspecified/Mixed	2	128	4.17 (2.5, 5.84)			
**Measurement method**						
AAS^d^	92	13,314	2.91 (2.67, 3.14)	0.001	0.001	
FAAS^d^	51	6577	2.60 (2.46, 2.74)			
ICP-AES^d^	35	5681	2.39 (2.27, 2.51)			
ICP-MS^d^	53	10,210	2.55 (2.19, 2.91)			
Other	12	2082	3.07 (2.55, 3.59)			

^a^ P-value for difference between groups without ‘Unspecified/Mixed’ group. ^b^ P-value for difference between groups for all groups. ^c^ Key studies include only studies conducted in healthy women, healthy and term infants, and exclusively breastfed if aged 0-5.9 months. ^d^ Recommended methods for determination of zinc are AAS, FAAS, ICP-MS and ICP-AES (14, 19). NA: Not applicable, N: number of studies, n: number of participants

**Fig. 3:**
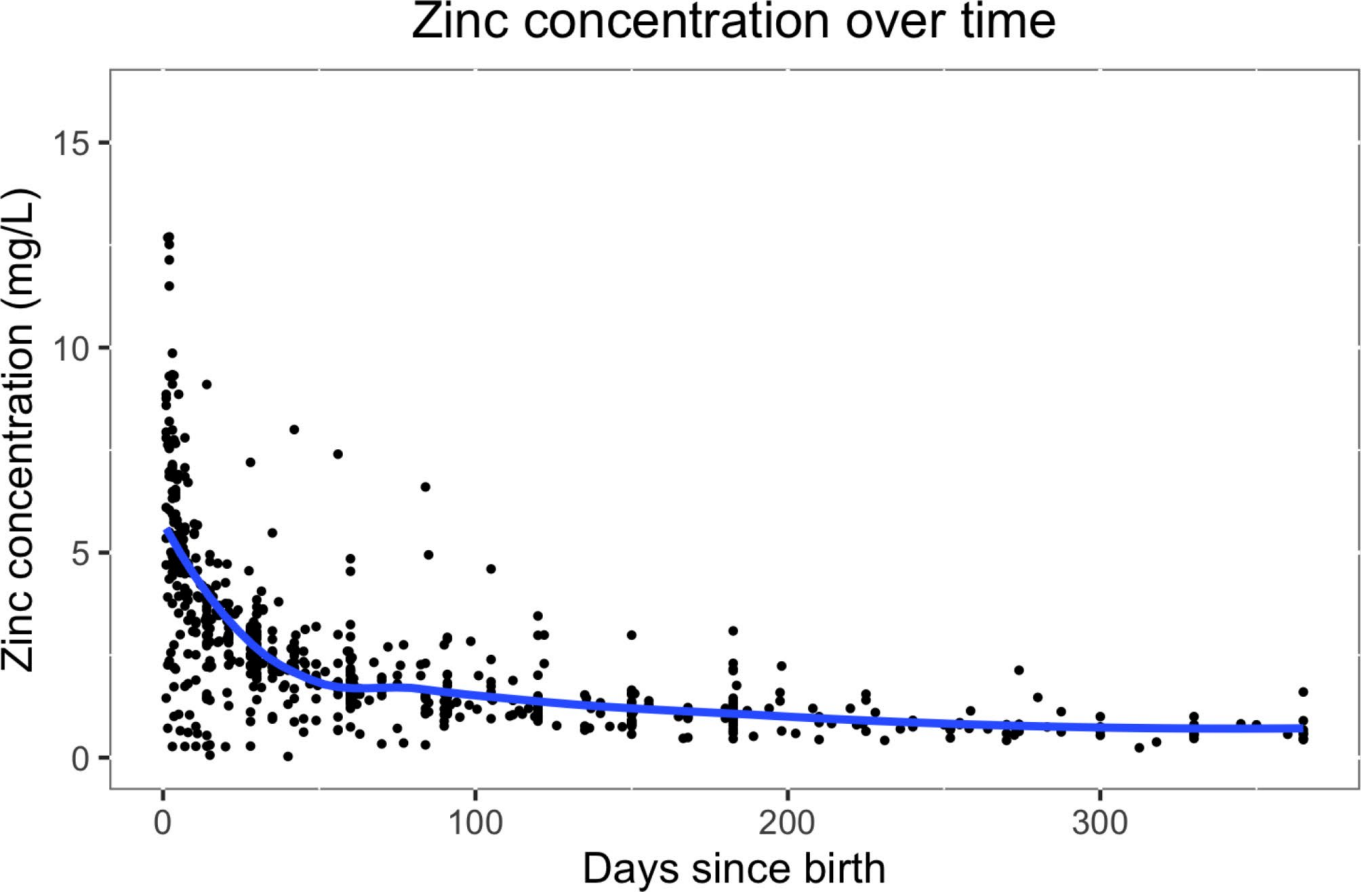
Zinc concentration (mg/L) over time, trendline (blue) fitted with local polynomial regression

There were significant differences between lactation stages (see Table 2). The evolution of zinc concentration in breast milk over time is shown in Fig. 3. Zinc concentration starts high and then decreases rapidly until reaching a plateau. The evolution of zinc concentration over time was best modeled with a logarithmic model. When including all studies across all ages, the zinc concentration [mg/L] at a certain timepoint could be estimated with the equation: 5.9514 + 0.0005 ✕ days – 2.0266 ✕ log(days). When restricting to ‘key’ studies across all ages, the fitted model was 8.4457 + 0.0031 ✕ days – 3.4048 ✕ log(days). When restricting to studies conducted among 0-5.9 month-olds, the model was 5.7859 – 0.0001 ✕ days – 1.9218 ✕ log(days) and, when restricting to ‘key’ studies and among 0-5.9 month-olds, 9.1174 – 0.0036 ✕ days – 3.8383 ✕ log(days).

There were no significant differences between healthy and unhealthy populations, between mothers of preterm and term infants, and between mothers with good or poor nutritional status. However, there were significant differences between maternal age groups, breastfeeding practices, maternal zinc supplementation, country income categories, continents, and measurement methods. Given the influence of time since birth on zinc concentration, many of the sub-group differences could have been confounded by differences in time since birth. Therefore meta-regressions were conducted with the sub-groups, controlling for the numbers of days since birth. When including time since birth as a covariate, the differences between measurement methods and continents became non-significant. However, the differences between maternal age groups, gestation, breastfeeding practices, nutrition, and country income categories remained: The zinc concentrations were higher in adult mothers, in mothers of preterm infants, in mothers exclusively breastfeeding, in mothers with good nutrition, and in mothers living in upper-middle and high income countries. Zinc concentration in breast milk, controlling for age, did not differ significantly over the years of the publication (p = 0.594).

The effect of maternal zinc intake on breast milk concentrations was further investigated. Nine trials looked at the effect of zinc supplementation on zinc concentration in breast milk. When meta-analyzed, no significant differences were found between levels of zinc supplementation (MD: 0.11, 95% CI: -0.18, 0.40). One cohort study [20] compared women with different zinc intake levels and found no differences in zinc concentration in transitional milk, but a higher concentration in women with higher intakes in mature milk (p < 0.05).

The effect of genetic variations was investigated in two studies [21, 22]. These studies found that variants of the zinc ZnT2 (SLC30A2) were common and could be associated with low zinc concentrations in breast milk.

### Vitamin D

A total of 43 studies with 3,726 participants reporting on vitamin D concentration of breast milk were included. The detailed characteristics of the studies included are shown in **Additional File 4**. Among those studies, 19 were cross-sectional studies, 8 were cohorts, 12 were trials and 4 were case-control studies. The studies were conducted in Asia (N = 21), North America (N = 13), Europe (N = 12), Africa (N = 2), and Australasia/Oceania (N = 2). No studies were identified in South America. The countries in which the studies were the most conducted were the United States (N = 11) and Japan (N = 6). Studies were published between 1981 and 2021, with 25% published before 1989 and 25% published after 2017. Most of the studies included healthy participants (N = 24), but many did not report the health status of their participants (N = 15). Four studies included both healthy and unhealthy participants. The health conditions included were in the mother, COVID-19 (N = 1), HIV (N = 1), or long-term hospitalization (N = 1), and in the infant, atopic dermatitis (N = 1). Most of the studies did not report the nutritional status of the mothers included (N = 38), a few reported good nutritional status (N = 4), or both good and poor nutritional status (N = 1). The analytical methods the most used to determine vitamin D concentrations were liquid chromatography with tandem mass spectrometry (LC-MS/MS) (N = 13), high performance liquid chromatography (HPLC) and competitive protein-binding assay (CPBA) (N = 14), HPLC (N = 4), ultra-violet HPLC (UV-HPLC) (N = 3), and radioimmunoassay (RIA) (N = 2). The preferred methods to measured vitamin D in breast milk are HPLC and CPBA or LC- MS/MS [14, 19]. Sixteen studies reported the use of external standards for validation (2 studies participated in a vitamin D standardization program) and 25 studies did not report the use of any external standard.

The concentrations of the different vitamin D forms are shown in Table 3. The mean total ARA of breast milk was 58 IU/L (95% CI: 45, 70). The different forms of vitamin D contributed to total ARA in the following decreasing order: 25OHD3, vitamin D3, 25OHD2 and vitamin D2. Fifteen studies reported undetectable levels of vitamin D in all [23–25] or some of the breast milk samples [26–37]. Several studies had extremely low or high values, however none of the studies were found to be both highly outlying and influential.

**Table 3:** Concentration of different forms of vitamin D (IU/L)

Vitamin D form	N	n	Mean (95% CI)	Range
**Vitamin D**	20	2400	17 (13, 20)	0-600
** Vitamin D2**	13	1264	2 (2, 3)	0-353
** Vitamin D3**	16	1549	15 (11, 19)	0-1896
**25OHD**	24	2470	80 (66, 93)	0-7065
** 25OHD2**	12	975	5 (4, 6)	0-1052
** 25OHD3**	17	1249	44 (34, 54)	0-4411
**Total ARA**	19	2162	58 (45, 70)	1-236

N: Number of studies, n: number of participants

The results of the sub-group meta-analyses for vitamin D, 25OHD and total ARA are shown in Table 4. There were significant differences between infant age groups, country income categories and continents. However there were very few studies in certain sub-groups, making it difficult to interpret. Vitamin D levels were higher in women receiving supplementation than those not (total ARA: 91 IU/L, 95% CI: 73, 109 vs. 48 IU/L, 95% CI: 34, 63). Levels tended to be higher in summer than in other seasons (total ARA summer: 117 IU/L, 95% CI: 108, 126, fall: 63 IU/L, 95% CI: 52, 73, winter: 66 IU/L, 95% CI: 45, 87, spring: 58 IU/L, 95% CI: 50, 66). There were no significant differences between measurement methods. The meta-regressions found a significant association between latitude and 25OHD2, 25OHD3, vitamin D2, and vitamin D3 concentrations, but not for 25OHD, vitamin D, and ARA. Total ARA of milk did not appear to change over the years (p = 0.750).

**Table 4:** Meta-analyses for vitamin D concentration (IU/L)

Group	Vitamin D				25OHD				Total ARA			
	N	n	Mean (95% CI)	p^a^	N	n	Mean (95% CI)	p	N	n	Mean (95% CI)	p
All studies	20	2400	17 (13, 20)	NA	24	2470	80 (66, 93)	NA	19	2162	58 (45, 70)	NA
All studies without outliers	18	2364	18 (14, 22)		20	2013	50 (41, 59)		18	2146	60 (49, 72)	
**Infant age**												
0–5.9 months	14	1404	12 (9, 14)	0.053	21	1870	115 (92, 138)	< 0.001	20	1777	65 (55, 75)	< 0.001
6–11.9 months	2	172	6 (-3, 15)		1	69	81 (79, 83)		3	124	125 (21, 230)	
12–35.9 months	0	0	-		2	70	51 (-30, 133)		0	0	-	
Unspecified	7	918	23 (13, 33)		5	312	46 (23, 69)		4	210	22 (6, 37)	
**Vitamin D supplementation**												
Supplemented	6	620	11 (8, 14)	0.007	7	659	137 (85, 189)	0.032	10	968	91 (73, 109)	< 0.001
Not supplemented	9	589	8 (4, 11)		8	389	71 (39, 103)		9	543	48 (34, 63)	
Unspecified/Mixed	10	1293	21 (13, 29)		15	1295	64 (49, 79)		9	592	36 (24, 49)	
**Country income category**												
Low	1	41	35 (27, 44)	< 0.001	2	62	26 (-5, 56)	< 0.001	1	41	82 (73, 92)	< 0.001
Lower-middle	3	290	24 (11, 36)		1	101	32 (29, 34)		1	101	46 (42, 51)	
Upper-middle	4	315	15 (3, 26)		3	465	1219 (1069, 1368)		1	20	14 (11, 17)	
High	19	1754	15 (12, 18)		22	1842	61 (48, 74)		20	2000	56 (44, 67)	
**Continent**												
Africa	1	41	35 (27, 44)	< 0.001	2	62	26 (-5, 56)	< 0.001	1	41	82 (73, 92)	< 0.001
Asia	11	1211	16 (10, 21)		8	850	143 (107, 178)		8	921	37 (26, 48)	
Australasia/Oceania	2	166	4 (-4, 12)		2	166	22 (-21, 66)		2	166	26 (-24, 77)	
Europe	8	748	18 (13, 23)		9	1245	127 (91, 163)		5	774	75 (54, 97)	
North America	5	234	22 (14, 29)		7	147	49 (37, 62)		7	260	70 (44, 96)	
**Season**												
Fall	2	54	45 (-32, 122)	0.053	1	40	58 (48, 68)	0.055	1	40	63 (53, 73)	< 0.001
Spring	2	99	10 (6, 15)		1	85	50 (42, 58)		1	85	58 (50, 66)	
Summer	3	313	9 (4, 13)		3	262	88 (60, 116)		2	256	117 (108, 126)	
Winter	4	434	5 (4, 6)		3	381	60 (39, 82)		3	381	66 (45, 87)	
**Measurement method**												
HPLC and CPBA	7	489	16 (11, 22)	0.293	9	550	51 (39, 64)	< 0.001	8	517	68 (46, 91)	0.360
LC-MS/MS	7	852	13 (8, 18)		8	1315	60 (39, 80)		9	1558	50 (29, 71)	
Other	6	1059	22 (11, 32)		7	605	497 (409, 585)		2	87	66 (56, 75)	

^a^ P-value for difference between groups. NA: Not applicable, N: number of studies, n: number of participants

Not enough studies reported information on sun exposure and skin pigmentation to be able to conduct these sub-group meta-analyses. One trial [38] found that UVB irradiation could increase vitamin D breast milk concentration. A cross-sectional study [39] found higher vitamin D3, D2 and 25OHD3 in White women than in Black women (p = 0.002, 0.001, and 0.03 respectively), but no differences in 25OHD2 levels (p = 0.21).

An analysis of the studies who specifically looked into the relationship between vitamin D supplementation and vitamin D concentration in breast milk was conducted. A total of 10 trials [26, 27, 31, 32, 35, 37, 40–43] investigated the effect of vitamin D supplementation on breast milk concentration. All studies, except one [40], concluded that vitamin D supplementation could significantly increase vitamin D concentration in breast milk. When meta-analyzed, a significant increase was found in breast milk of mothers receiving vitamin D supplementation in vitamin D3 (MD: 28 IU/L, 95% CI: 6, 50), and ARA (MD: 53, 95% CI: 28, 77), but not vitamin D (MD: 4, 95% CI: -1, 9) or 25OHD (MD: 9, 95% CI: -14, 32). One cohort study [36] found that mothers taking vitamin D supplements had higher vitamin D breast milk concentrations.

## Discussion

### Summary of results

This systematic review included a large number of studies from all continents. The mean calcium concentration in breast milk was 261 mg/L. Calcium concentration was stable over time, decreasing only very slowly, and was stable across most maternal and child characteristics. The mean zinc concentration in breast milk was 2.57 mg/L. Zinc concentration was high in the first weeks post-partum followed by a rapid decrease over the first months and then relatively stable. Several additional factors, such as maternal age, gestational age, and maternal nutrition, influenced zinc concentration in breast milk. The mean total ARA of breast milk was 58 IU/L and consisted of mostly 25OHD3. The large variation between vitamin D estimates could be partly explained by differences in measurement methods, supplementation, countries and seasons.

### Interpretation

#### Calcium

The calcium concentrations found in our review are similar to the ones found in other less recent and less extensive reviews [44–48]. The slow and small decrease of calcium over time was also found in another review [49]. The lack of differences between term and preterm infants [46] and the lack of effect of several maternal conditions [50] were also found in other reviews. A review found that adolescent mothers and specific conditions such as familial hypophosphatemia and hyperparathyroidism could affect calcium concentrations, but no other environmental or constitutional parameter [48].

#### Zinc

The zinc concentration found in our review is similar to ones found in other less recent and less extensive reviews [44, 45, 47, 51, 52]. The rapid decline of zinc concentration during the first days post-partum was also found in other reviews [49, 52, 53]. The lack of effect of several maternal health conditions was also found in another review [50]. One review [51] found lower values for preterm than for term infants, which is the opposite to what was found in our review. The higher concentrations of zinc in preterm milk found in our review could be hypothesized to be an adaptation of the breast milk contents to better fit the needs of preterm infants, which need higher concentrations of zinc in smaller quantities of breast milk.

#### Vitamin D

Very few reviews on vitamin D concentration in breast milk were identified. One large systematic review found only 1–2 studies for vitamin D [14] and another systematic review looking at preterm milk did not identify any studies [54]. The large variations in vitamin D concentration found in our review could be partly explained by supplementation and season. Several factors (e.g. dietary intake of vitamin D, sun exposure, skin pigmentation) which could have strongly influenced levels and confounded sub-group differences were not reported in sufficient studies to allow meaningful analyses. The large variations could also have been due to the lack standardization of the methods used to measure vitamin D forms [55] and the use of methods not validated for the medium breast milk (which contains much higher fat proportions than other mediums, such as plasma serum and urine). In addition, factors such as pre-processing or storage could have had an influence on the levels found. For example, one study [56] found that milk stored in plastic containers were found to have lower levels of vitamin D than in glass containers, possible due to the absorption of vitamin D by the plastic.

#### Translation into nutrient requirements

Based on the assumption that breast milk provides adequate amounts of the majority of nutrients for optimal growth of infants [1] and that exclusive breastfeeding is recommended up to 6 months of age [1, 2], the results of this review can be used to estimate the requirements of infants up to 6 months of age. When taking the age-specific high quality estimates for calcium and zinc concentrations in breast milk multiplied by the age-specific intake of breast milk [57], breast milk is estimated to provide 181 mg/day of calcium and 1.6 mg/day of zinc over the first 6 months of life. These estimates for calcium and zinc could be used to estimate the adequate intake (AI) in infants 0–6 months old. As a comparison, the Institute of Medicine recommends an AI for calcium of 200 mg/d [58] and for zinc of 2 mg/d [59] for infants up to six months of age. Due to the low ARA of breast milk, the calculated values are not appropriate to estimate AI in vitamin D in infants 0–6 month old [60].

### Strengths and limitations

A strength of this review is the considerate effort that was made to review the most extensive literature available on the topic. In fact, an extensive systematic search strategy was developed and complemented with hand searches, including searches of African and Latin American literature databases. Moreover, eligibility criteria were kept wide, with no restrictions for dates of publication or language. When looking at the geographical distribution of the studies included, one can conclude that studies in most regions of the world could be identified and included in our review. Another strength of this study is the multiple sub-group analyses and meta-regressions conducted to compare different groups of populations and study influencing factors.

A limitation of this review was the quality of the studies included and the information available. Several studies did not provide sufficient information on influencing factors, especially on nutrition status. Moreover, the statistical heterogeneity was high for all three nutrients (I^2^ of 100% and significant τ^2^). There was also an uneven distribution of the studies between some of the sub-groups (i.e. healthy vs. unhealthy, adult vs. adolescent mothers, with vs. without supplementation) indicating that the results of these sub-group analyses should be interpreted with caution [12]. To mitigate this limitation, for zinc, the differences between the sub-groups were further investigated by controlling the effect of time since birth.

## Conclusion

This review provides global estimates of calcium, zinc and vitamin D content in breastmilk and indications on changes over time and other influencing factors. Results of the review can be used as an aid in assessing infant and young child nutrition, including the estimation of nutrient intakes and requirements.

## Electronic supplementary material

Below is the link to the electronic supplementary material.

Supplementary Material 1

## Data Availability

The complete datasets used during the current study are available from the corresponding author on reasonable request.

## List of abbreviations

25OHD: 25-hydroxy-vitamin D
AAS: Atomic absorption spectroscopy
AIC: Akaike information criterion
ARA: Anti-rachitic activity
CENTRAL: Cochrane Central Register of Controlled Trials
CI: Confidence interval
CPBA: Competitive protein-binding assay
FAAS: Flame atomic absorption spectroscopy
FAO: Food and Agriculture Organization
HPLC: High-performance liquid chromatograph
ICP-AES: Inductively coupled plasma atomic emission spectroscopy
ICP-MS: Inductively coupled plasma mass spectrometry
LC-MS/MS: Liquid Chromatography Tandem Mass Spectrometry
LILACS: Literatura Latino-Americana e do Caribe em Ciências da Saúde
MD: Mean difference
NA: Not applicable
SD: Standard deviation
UVB: Ultraviolet B
