# Supplementary material for: Calcium, zinc, and vitamin D in breast milk: a systematic review and meta-analysis
Source: Int Breastfeed J. 2023 Jun 1;18:27. doi: 10.1186/s13006-023-00564-2 (PMC10233556; doi:10.1186/s13006-023-00564-2)
Supplement: Supplementary file 1 — Supplementary Material 1 [file 13006_2023_564_MOESM1_ESM.docx]

# ADDITIONAL FILES

**Calcium, zinc, and vitamin D in breast milk:**

**a systematic review and meta-analysis**

# ADDITIONAL FILE 1. Search strategy

**Embase and Medline**

1. **'breast milk'/exp OR 'breast feeding'/exp**
2. **breastfed OR breastfeed OR breastfeeding OR 'breast fed' OR 'breast feed' OR 'breast feeding' OR 'breast milk$’ OR 'breast milk$’ OR 'human* milk' OR 'mother* milk$' OR 'woman* milk$' OR 'women* milk$' OR ((lactating OR lactation) AND milk)**
3. **1 OR 2**
4. **'zinc'/exp OR zinc OR 'zn'**
5. **'calcium'/exp OR calcium**
6. **'vitamin d'/exp OR 'vitamin d$' OR cholecalciferol$ OR ergocalciferol$ OR calciferol OR calcidol OR 25$OH$D$ OR '25-hydroxy vitamin D$' OR '25-hydroxyvitamin D$' OR calcifediol OR '25-hydroxycholecalciferol' OR '25-hydroxy cholecalciferol'**
7. **(micronutrient$ OR nutrient$ OR vitamin$ OR mineral$) NEAR/2 (content$ OR composition OR level$ OR intake$)**
8. **4 OR 5 OR 6 OR 7**
9. **3 AND 8**
10. **('animal'/exp OR 'nonhuman'/exp OR 'animal cell'/exp OR 'animal cell culture'/exp OR ‘animal experiment’/exp OR ‘animal tissue’/exp OR ‘animal model’/exp) NOT (('animal'/exp OR 'nonhuman'/exp OR 'animal cell'/exp OR 'animal cell culture'/exp OR ‘animal experiment’/exp OR ‘animal tissue’/exp OR ‘animal model’/exp) AND 'human'/exp)**
11. **10 NOT 11**

**Cochrane CENTRAL**

1. “milk, human”/exp OR “breast feeding”/exp
2. breastfed OR breastfeed OR breastfeeding OR “breast fed” OR “breast feed” OR “breast feeding” OR “breast milk$” OR “breast milk$” OR “human* milk” OR “mother* milk$” OR “woman* milk$” OR “women* milk$” OR ((lactating OR lactation) AND milk)
3. 1 OR 2
4. “zinc”/exp OR zinc OR “zn”
5. “calcium”/exp OR calcium
6. “vitamin d”/exp OR “vitamin d$” OR cholecalciferol$ OR ergocalciferol$ OR calciferol OR calcidol OR 25$OH$D$ OR “25$hydroxy vitamin D$” OR “25$hydroxyvitamin D$” OR calcifediol OR “25$hydroxycholecalciferol” OR “25$hydroxy cholecalciferol”
7. (micronutrient$ OR nutrient$ OR vitamin$ OR mineral$) NEAR/2 (content$ OR composition OR level$ OR intake$)
8. 4 OR 5 OR 6 OR 7
9. 3 AND 8

#

# ADDITIONAL FILE 2. Characteristics of studies included for breast milk calcium concentration

| **ID [Reference]** | **Study design** | **Country** | **Mother description** | **Child description** | **Milk description** | **Analytical method** | **Sample size** |
| --- | --- | --- | --- | --- | --- | --- | --- |
| Abdel-Kader 1969 (1) | Controlled trial | Egypt | Women 18-40 years, given placebo+IUCD, Lyndiol 2.5, Lyndiol 1.0, Lynestrenol 0.5 or Deladroxate injection. Data from baseline were used in the analysis. |  | Collection of milk samples before contraception and then every 2 weeks for 16 weeks, all milk at 9 and 12 AM from one breast (opposite to one given to infant), extracted with mechanical pump. | Method developed by Abdel Rehim and Dokhana 1960 | 50 |
| Adesiyan 2011 (2) | Case-control study | Nigeria | Women 26-46 years, taking or not hormonal oral or injectable contraceptives. |  | Collection of mature milk, approximately 8 months pp, manually. Stored at -20°C until analysis. | Spectrophotometry | 182 |
| Allen 1991 (3)^[[1]](#footnote-1)^ | Cohort study | USA | Women 25-39 years, non-smoking, Caucasian, multiparous, who had successfully breast-fed at least one infant previously, who planned on exclusive breastfeeding for >=6 months and partial breast-feeding for >=1 year. | Infants from 0 days old to >=1 years old. | Collection of milk (2x/d for 3 days pp, 1x/d up to 7 days pp, 1x/2d up to 14 days, 1x/week up to 8 weeks pp, and 1x/month for ≥1 years pp) after 2-3 min nursing at midmorning, extracted by hand. Stored and transported on ice within 24h and stored -70°C until analysis. | AAS | 13 |
| Anastacio 2004 (5) | Cross-sectional study | Brazil | Healthy women 19-43 years (mean age: 28), BMI 18.3-34, exclusively or predominantly breast-feeding, no history of occupational or accidental lead exposure and no complications during pregnancy or lactation. |  | Collection of mature milk (20-102 days postpartum, mean 56 days postpartum), extracted with manual pump. Stored at 4°C up to 2h after collection and frozen at -70°C until analysis. | ICP-OES | 38 |
| **Anderson 1992b** (6) | Cohort study | USA | Women 20-30 years, graduates or wives of graduates, adequate diets, no vegetarian. | Healthy full-term infants, exclusively breastfed. | Collection of colostrum/transitional/mature milk from 1 to 164 d pp, with breast pump or plastic breast shield, from morning feed. Frozen until analysis. | ICP-AES | 6 |
| Arias-Borrego 2022 (7) | Case-control study | Spain | Women with COVID-19, taking no drugs and not in intensive care, mean age 35±4 y, and healthy prepandemic controls, mean age 31±5 y. | Infants, mean birthweight 3.3 kg, 77-80% exclusively breastfed. | Collection of colostrum, transitional and mature milk, during morning, following standardized protocol. Stored at -20 and then -80°C. | ICP-MS | 55 |
| Arruda Carneiro 1973 (8) | Cohort study | Brazil | Women 19-40 years, from low (n=10) and high (n=10) SES, no signs of nutritional deficiency. | Normal infants. | Collection of colostrum, transitional and mature milk at 1-5, 7, 14, 28, 46, 82, 110, 138, 159, 186 d pp, from first breast of previous feeding, in the morning, by hand expression. | Colorimetric | 20 |
| Arver 1984 (9) | Cohort study | Sweden | Healthy women. |  | Collection of colostrum/transitional milk 0-2 and 4-6 d pp. Stored at 4°C and then frozen until analysis. | AAS | 75 |
| Astolfi 2018 (10) | Cross-sectional study | Italy | Women, living in Rome. |  | Collection of mature milk, 4-6 weeks pp, extracted with manual pump or passive breast milk sampler. Stored at 4°C and then -20°C.. | ICP-MS | 20 |
| Atkinson 1980 (11) | Cohort study | Canada | Women, mean age 28-31±1 years, no pre-eclampsia, not taking diuretics. | Infants, preterm (n=13, GA 26-33 weeks, two sets of twins, only one survived in each set) and term (n=10, GA 38-40 weeks), AGA. | Collection of colostrum/transitional/mature milk 0-30 d pp, 24h expressions, both complete breasts, with manual or electric pump. Stored at -20°C until analysis. | FAES | 23 |
| Atkinson 1983 (12) | Cohort study | Canada |  | Low birth weight (<1.3 kg) infants, AGA, mean GA 28-29±1 weeks, no major congenital abnormality, tolerance to oral feedings within the first 48h of life, no requirements for respiratory support by ventilator on entry to the study. | Collection of transitional and mature milk 6-9, 13-15, 26-28 d pp. Analyzed within 48h. | FAES | 7 |
| Barakat 1969 (13) | Cross-sectional study | Egypt | Healthy women. |  |  | Method of Ling 1958 | 50 |
| Barltrop 1974 (14) | Cross-sectional study | UK |  |  | Collection of colostrum/transitional and mature milk at 26-140 h, 6 d, 6 weeks pp, from one breast, at beginning of midday feed. | AAS | 58 |
| Bauer 2011 (15) | Cohort study | Germany | Healthy women, with sufficient milk supply to give sample, no hypertension or diabetes, not vegetarian or no special nutritional diet. | Preterm and term infants. | Collection of pooled 24h milk samples, extracted with electric pump, weekly from week 1-8 pp. Stored at 4°C and then -70°C until analysis. Calcium was measured with absorption spectrometer and colorimetric assay. | Spectrophotometry | 102 |
| **Bezerra 2008** (16) | Cross-sectional study | Brazil | Healthy adolescents 15-18 years, mean BMI 23 ±4, mixed black and white ethnicity, non-smoking, no history of bone or renal disorders affecting calcium metabolism. | Full-term infants, exclusively or predominantly breastfed. | Collection of mature milk at 2-24 weeks pp (mean 11 weeks pp), extracted manually. Stored at -20°C until analysis. | ICP-OES | 40 |
| **Bilston-John 2021b** (17) | Cohort study | Australia | Women, non-smoking, high SES. | Healthy term infants, singleton, exclusively breastfed up to 5 months, continued breastfeeding up to 12 months. | Collection of milk samples over a 24h period, with manual expression, before and after feed. Storage at 20°C until analysis. | ICP-MS | 11 |
| Bjorklund 2012 (18) | Cross-sectional study | Sweden | Women, mean age 29±4 years, BMI 23±4, primiparous, from a random sample. |  | Collection of mature milk sample during 7 days (during 3rd week postpartum, 14-21 days pp), sampling from both beginning and end of breastfeeding session, extracted with manual pump or passive sampler extraction. Stored in freezers until analysis. | ICP-MS | 60 |
| Bloom 1978 (19) | Cross-sectional study | Australia | Women 23-27 years. |  | Stored in refrigerator until analysis. | AAS | 3 |
| Bocca 2000 (20) | Cross-sectional study | Italy | Women 19-40 years old, Italian. |  |  | ICP-AES | 60 |
| Bolognini Pereira 2013 (21) | Cohort study | Brazil | Adolescents <18 years old, no infectious disease, not taking nutritional supplements, no medication. | Infants, exclusively or predominantly breastfed. | Collection of mature milk, extracted manually at 6, 10, 14 weeks pp, from breast not sucked in last feeding, 9-10AM. | AAS | 51 |
| Bosscher 2001 (22) | Cross-sectional study | Belgium |  |  | Pooled mature milk sample. | FAAS | 4 |
| Bosscher 2003 (23) | Cross-sectional study | Belgium |  |  | Pooled mature milk sample. | FAAS | 4 |
| Braga 2007 (24) | Cross-sectional study | Brazil |  |  | Pool of donor milk, extracted manually, minimum 30 d pp, no processing. | AAS | 12 |
| Butte 1984 (25) | Cohort study | USA | Women 20-35 years, parity <=2, max 2 servings/d of carbonated beverages, coffee, tea and alcohol, no routine medication, no steroid contraceptives, good health (all, except n=2 with hypertension in pregnancy), taking vitamin-mineral tablets (all, except n=2), non- and smoking (n=4). | Term (>=37 weeks) and preterm (<37 weeks) infants, no congenital or acquired disease, exclusively breastfed (all, except a few cases who were introduced foods at 6-8 weeks and some preterm infants also given formula). | Collection of mature milk at the end of week 2, 4, 6, 8, 10, 12 pp, 8-12 AM, at least 2h after feeding, with breast pump. Stored at 4 and then -20°C.. | AAS | 21 |
| **Butte 1987a** (26)^[[2]](#footnote-2)^ | Cohort study | USA | Healthy women 18-36 years, non-smoking, no long-term medication, parity 1-2, middle-upper SES, White (n=41), Hispanic (n=2), Asian (n=1) and West Indian (n=1), vaginal (84%) and cesarian (16%) delivery. | Heathy full-term AGA infants, exclusively breast-fed, at 1, 2, 3, and 4 months pp, birth weight 2.6-4.6 kg. | Collection of a complete milk sample from the breast opposite than offered to the infant, with electric pump, at 1, 2, 3, and 4 months pp. Stored at -20°C until analysis. | AAS | 45 |
| Butte 1987b (30) | Case-control study | USA | Moderately controlled type 1 diabetic women, otherwise healthy, non-smoking, not taking oral contraceptives. Healthy non-diabetic women as controls. | Healthy infants, no congenital abnormalities, 90 days old, exclusively breastfed. | Collection of 24h mature milk at 3 months pp, alternatively breast opposite to the one feeding the infant, complete breast, extracted with electric pump. | AAS | 5 |
| **Butts 2018** (31) | Cross-sectional study | New Zealand | Healthy women 19-42 years, BMI 20-39, breastfeeding, of different ethnicities (10% Asian, 22% Maori & Pacific Island or 68% European), not dieting, no health disorder. | Full-term infants 6-8 weeks old, exclusively or primarily breastfed, without neonatal care, birth weight 2.4-4.6 kg. | Collection of 3 mature milk samples over 1 week, before first feed of the day, extracted by hand or breast pump. Stored at -18°C, thawed and then stored again at -80°C until analysis. Calcium was measured with ICP-MS. | ICP-MS | 78 |
| **Bzikowska-Jura 2022** (32) | Cross-sectional study | Poland | Healthy women, ≥18 y, no gestational diabetes, no smoking, sufficient milk supply. | Term infants 6-8 weeks old, exclusively breastfed, singleton. | Collection of mature milk 6-8 weeks, 4 times during a 24h period. Stored at -20°C until analysis. | ICP-MS | 30 |
| Campbell-Yeo 2010 (33) | Randomized controlled trial | Canada | Women, delivered preterm infant experiencing lactation failure, no medication that affects domperidone, no mastitis, no chronic illness, no previous breast surgery, no lactose intolerance, randomized to domperidone or placebo for 2 weeks. Data from placebo group were used. | Preterm (<31 weeks) infants. | Collection of milk, with electric pump, at baseline and after 2 weeks. | Chemistry analyzer | 24 |
| Cancela 1986 (34) | Cohort study | France | Women, mainly African origin, not taking vitamin D supplements (all except one, taking 1200 vitamin D2 IU/d). | Full-term infants, 0-1 month. | Collection of colostrum (3-5d pp), transitional (15-18d pp), and mature milk (30-45d pp), extracted with manual pump. Stored at -20°C until analysis. | AAS | 13 |
| Capriati 2019 (35) | Cross-sectional study | Italy | Women 25-37 years, in human milk-donating program, non-smoking, not taking nicotine replacement, no recreational drugs, little alcohol, HIV/hepatitis B or C/HTLV/syphilis negative, no active tuberculosis, no medication, no contaminated milk sample. |  | Milk samples from donors. Stored at -20°C until analysis. | Chemistry analyzer | 25 |
| **Carias 1997** (36) | Cohort study | Venezuela | Healthy women 18-35 years, living in metropolitan Caracas, no chronic disease, no anemia, no signs of malnutrition or vitamin deficiency, mainly from low income families and deficitary sanitary conditions. | Term infants. | Collection of colostrum (48-54h) and mature milk samples (at 1, 3, 6 months pp), extracted manually from breast opposite to the one offered to the infant, in morning and evening. Stored at -20°C until analysis. | AAS | 45 |
| Castro 2014 (37) | Cohort study | Chile | Women 15-40 years old, mean BMI 27-28 (range 21-39), primi- and multiparous, living in major mine tailing deposition (n=24) or control area (n=11). | Term singleton infants, at least 90% exclusively breastfed, at 2-4 months and 4-6 months of age. | Collection of mature milk samples at 2-4 months pp, in the morning. Stored at -80°C until analysis. | ICP-MS | 35 |
| Chan 1982 (38) | Cohort study | USA | Women, eating regular diet, taking daily multivitamins including 400 IU vitamin D2 and 250 mg calcium. | Preterm and term infants, AGA, exclusively breastfed, received daily vitamin D supplements. | Collection of milk on 2-4 d, 14-21 d and 26-31 d pp, extracted manually the first 9 AM morning milk, fore and hindmilk. | AAS | 25 |
| Chen 2007 (39) | Cross-sectional study | China | Healthy women 18-36 years, non-smoking, no chronic medication, parity of 1 or 2. |  | Collection of mature milk at 1, 2, 3, 4, 5, 6 months pp. | ICP-AES | 240 |
| Citrakesumasari 2019 (40) | Cross-sectional study | Indonesia |  | Full-term infants 2-6 weeks old, breastfed, normal weight (n=31) or low birth weight (n=6). | Collection of mature milk (2-6 weeks pp), manually and then with pump. Stored in a freezer until analysis. | AAS | 37 |
| Codo 2018 (41) | Cross-sectional study | Brazil | Milk donors for term infants and mothers of preterm infants, mean age 25 ±6 y. | Term and preterm infants. | Collection of milk at different moments of the day. Stored in freezer until analysis. | ICP-OES | 68 |
| Dagnelie 1992 (42) | Cross-sectional study | Netherlands | Healthy women, on macrobiotic or omnivorous diet. | Infants, 2.0-3.3 and 9.0-13.0 months pp. | Collection of mature milk, on 2 consecutive days, from opposite breast after finishing first breast. Stored at -4°C and then -20°C. | AAS | 31 |
| De Salim 2010 (43) | Cross-sectional study | Venezuela | Healthy women 18-40 years, non-smoking, no alcohol, no disease (mastitis, fever, mammary abscess, diabetes, hypertension, renal insufficiency, cancer, chronic neuropathy), no drugs, no medication, 78% from poor SES, 9% underweight. | Term infants 25-30 days old, exclusively breastfed, singleton. |  | AAS | 82 |
| Deng 2009 (44) | Cross-sectional study | China | Healthy women 18-36 years, non-smoking, no chronic medication, parity of 1 or 2. |  | Collection of mature milk, from 3 weeks to 2 months pp. | ICP-MS | 60 |
| **DeSantiago 2002** (45) | Cross-sectional study | Mexico | Women 18-35 years, parity 2-6, BMI mean 22-23±1-2 range 19-24, non-smoking, no oral contraceptives, no chronic illness, no alcohol abuse. | Term infants of 1, 3, 6, 12 months old, birth weight >=2.5 kg, exclusively breastfed for 6 months. | Collection of 24h milk samples at 8AM, 12AM, 7PM for 2 consecutive days. | AAS | 27 |
| Dewey 1983 (46) | Cohort study | USA | Women 20-36 years, high education, some taking supplements (n=16), parity 1-2. | Infants, 1-6 months, at least partially breastfed. | Collection of milk monthly (1, 2, 3, 4, 5, 6 months pp), from one breast at 2nd feeding of the morning, extracted by hand. Frozen until analysis. | FAAS | 20 |
| Dewey 1984 (47) | Cohort study | USA | Women 21-37 years, vegetarian and non-vegetarian. |  | Collection of milk at 4-6 months, 7-11 months and 12-20 months pp, extracted by hand or manual pump, at 2nd feeding of morning, monthly or bimonthly. Stored at -20°C until analysis. | FAAS | 46 |
| Dorea 1998 (48) | Cohort study | Brazil | Women, who used combination pill (n=12), mini-pill (n=21) or no hormonal contraceptive (n=21). |  | Collection of milk before contraception (2.5-4 months pp) and after (approximately 2 weeks later), 7-11:30 AM, expressed manually. Stored at -20°C until analysis. | ICP-AES | 54 |
| **Dumrongwongsiri 2021** (49) | Cross-sectional study | Thailand | Healthy women, no medication. | Term infants, 4-6 months old, exclusively and non-exclusively breastfed. | Collection of milk samples at 4-6 months pp, 2-5 PM, after cleaning breast, first 10-15 ML from one breast. Stored at -80°C until analysis. | ICP-MS | 34 |
| Dutta 2014 (50) | Cohort study | India | Women, mean BMI of 25, several with conditions (pregnancy induced hypertension, antepartum hemorrhage, oligohydramnios, prolonged rupture of membranes, preterm premature rupture of membranes). | Pre-term (<34 weeks) infants, singleton, exclusively breastfed for at least 4 months, mean birth weight of 1.3 kg. | Collection of transitional and mature milk (7, 28, 90 and 180 ±2 days postpartum), after 3 min of breastfeeding, extracted by hand. Stored -20°C until analysis. | Ion selective electrodes | 33 |
| El-Farrash 2012 (51) | Case-control study | Egypt | Women, mean age 25-27±3-5 years, mean BMI 27±3, mean parity 2.6±1, healthy or anemic (hemoglobin <11 g/dL), no iron supplementation during pregnancy, no other causes of anemia, no pregnancy-induced hypertension, no diabetes, no prolonged rupture of membranes, no fever or foul smelling liquor, no antepartum hemorrhage, no mastitis, no other systemic illness. | Term infants, mean birth weight 2.5-3.3±0.3-4 kg, exclusively breastfed, singleton, no hemolytic disease, no neonatal sepsis. | Collection of transitional/mature milk 15±3 d pp, by manual expression, after discarding 4-5 mL initial milk. Stored at -20°C until analysis. | AAS | 80 |
| Feeley 1983a (52) | Cohort study | USA | Healthy women 16-38 years, from middle SES, parity 1-2, 94% took supplements (96% calcium, 72% magnesium). | Healthy full-term infants, birth weight 2.5-4.8 kg. | Collection of colostrum (4-7d pp), transitional milk (10-14d) and mature milk (30-45d pp), from beginning, middle and end of feeding, from morning and evening feeds, extracted by hand or manual pump. Stored at -20°C until analysis. | ICP-ES | 102 |
| Fly 1998 (53) | Randomized controlled cross-over trial | USA | Healthy women 25-38 years, active, mean BMI 23±4, white, parity 1-3, with <2 risk factors for heart disease (i.e. family history of heart disease, smoking, high blood pressure, high total cholesterol, diabetes). | Infants, breastfed, >2-<8 months. | Collection of mature milk before exercise or rest period, extracted with electric pump. Stored at -15°C until analysis. | ICP-AES | 14 |
| Fransson 1982 (54) | Cross-sectional study | Sweden | Healthy women. |  | Collection of milk, 2nd feeding of the day, 9-11 AM, one complete breast, with hand pump. Stored at -20°C until analysis. | AAS | 23 |
| Fransson 1983 (55) | Cross-sectional study | USA | Healthy women. |  | Collection of mature milk (0.5-12 months pp), one complete breast, 2nd feeding of the day, extracted with manual pump. | AAS | 30 |
| Fransson 1984 (56) | Cross-sectional study | Ethiopia, Sweden | Healthy women, normal blood pressure, no glucose or protein in urine, normal hemoglobin values, normal deliveries, from Ethiopia (n=18, mean age 19-23) with iron-rich diet from both non- and privileged groups and women from Sweden (n=23, mean age 28) with normal pregnancy and no medication but with supplements. | Infants, from Ethiopia (mean GA 38-41 weeks, mean birth weight 2.5-3.2 kg) and from Sweden (GA 37-42 weeks, mean birth weight 3.6 kg). | Collection of colostrum (4-5 d pp), from Ethiopia from both complete breasts at first feeding of day, from Sweden from one complete breast 2nd feeding of day. Stored at -20°C until analysis. | AAS | 41 |
| Friel 1999 (57) | Cohort study | Canada | Women 20-35 years, with no known pathological condition, not vegetarian, mainly European origin, well-nourished. | Full-term and preterm infants. | Extraction with electric pump (15 mL, at 2-3 days pp and week 2, 3, 4, 5, 6, 7, 8 and 12 pp), during a regular feed, 15 mL from one whole breast, between 10 AM and 2 PM. Stored at -20°C until analysis. | ICP-MS | 43 |
| Garg 1988 (58) | Cross-sectional study | India | Healthy women 18-30 years, parity 1-4, well-nourished (n=20) and under-nourished (n=15), vaginal delivery. | Full-term infants. | Collection of colostrum 0-3 days pp, in the morning before feeding. | AAS | 35 |
| **Garza 1983** (59) | Cohort study | USA | Healthy women, 26-35 years, parity 1-2, no medication, no oral contraceptive, limited coffee, tea and alcohol intake to <2/d, nonsmoking. | Healthy normal term infants, 5-7 months, AGA, exclusively breastfed at baseline and gradually weaning over 3 months. | Collection of mature milk samples at baseline, before weaning, and then during weaning every 2 weeks for 12 weeks, 8:00AM-12:00AM, from one complete breast 3-4h after previous nursing, extracted with pump. Stored at 4°C and then -20°C until analysis. | AAS | 6 |
| Gates 2021 (60) | Cohort study | USA | Women, 18-37 y, not vegan or vegetarian, not restricting caloric intake, HIV negative. | Preterm (≤33 GA) infants. | Collection of milk at 7, 14, 21 and 28 d pp, with electric breastpump, 24h collection, emptying both breasts every 3h. Stored at -29°C until analysis. Vitamin D was measured with UV-HPLC. | ICP-OES | 36 |
| **Gibson 2020** (61)^[[3]](#footnote-3)^ | Cohort study | Indonesia | Women, mean age 28±6 years, BMI at 5 months pp mean 24±4, 33% primiparous, from urban and rural site, no chronic disease, no acute malnutrition. | Healthy full-term infants, 2 and 5 months old, birth weight >=2.5 kg, exclusively breastfed, no chronic disease or malnutrition. | Collection of mature milk (2 and 5 months pp), extracted with pump, from one complete breast, in the morning. Frozen at -80°C until analysis. | ICP-MS | 212 |
| Goes 2002 (64) | Cross-sectional study | Brazil | Healthy adult women in general. | Term infants in general. | Human milk bank. Collection of mature milk, extracted by hand or manual/electric pump or drip milk. Stored at -20°C until analysis. | Colorimetric kit | 60 |
| **Greer 1982** (65) | Cohort study | USA |  | Healthy term infants, AGA, exclusively breastfed. | Collection of mature milk (3, 6, 12, 26 weeks pp), during first morning feed, fore-, mid and hindmilk, from one breast. Stored frozen until analysis. | AAS | 18 |
| Greer 1988 (66) | Cross-sectional study | USA | Women of preterm low birth weight infants who chose to supply their own milk. | Preterm infants, GA <32 weeks, low birth weight <1.6 kg, no major congenital anomality, no gastrointestinal disease, no seizure disorder requiring anticonvulsant therapy, AGA. | Collection of milk weekly. | AAS | 10 |
| Gross 1980 (67) | Cohort study | USA |  | Infants, term (n=18, 38-42 weeks GA) and preterm (n=33, 28-36 weeks GA). | Collection of colostrum (3 d pp), transitional (7, 14 d pp) and mature milk (21, 28 d pp), in the morning, both complete breasts, by manual or mechanical pump. Stored at -20°C. | AAS | 51 |
| Gulson 2001 (68) | Cross-sectional study | Australia |  |  |  | ICP-MS | 17 |
| Gupta 1984 (69) | Cohort study | India | Women, no anemia, no undernutrition, no vitamin deficiency. | Infants, term (n=50, mean GA 39 weeks) and preterm (n=14, mean GA 35 weeks). | Collection of colostrum/transitional/mature milk, (2-5, 6-10, 11-30 d pp) by manual expression, foremilk, in morning. Stored at -20°C until analysis. | FAAS | 64 |
| Gutikova 2007 (70) | Case-control study | Russia | Women with natural births, with no complications during pregnancy (n=96) or with gestosis (n=249). | Full-term infants. | Collection of transitional milk 6 days pp. | Chemistry analyzer | 345 |
| Harzer 1986 (71) | Cohort study | Germany | Healthy women. | Term infants. | Collection of milk 1, 3, 5, 15, 22, 29, 36 d pp, from both breasts complete during 24h, with electric breast pump. Stored at -20°C until analysis. | AAS | 10 |
| Hibberd 1982 (72) | Cohort study | Germany | Healthy women. | Term infants. | Collection of transitional and mature milk during the first 5 weeks (3, 5, 7, 11, 18, 25, 32, 39 d pp), both complete breasts, extracted with electric pump. Stored at -20°C until analysis.. | Colorimetric | 10 |
| Honda 2003 (73) | Cross-sectional study | Japan | Women 19-38 years old, Japanese, living in non-industrial area close to the hospital, vaginal and cesarean section. |  | Collection of transitional milk 5-8 d pp. Stored at -20°C until analysis. | ICP-AES | 68 |
| **Hou 2008** (74) | Cross-sectional study | China | Healthy women 18-36 years, non-smoking, no chronic medication, parity of 1 or 2. | Term infants, exclusively breastfed. |  | AAS | 240 |
| Hsu 2014 (75) | Cohort study | Taiwan | Healthy women, mean age 30-33 ±5 y, mean BMI 24 ±2. | Preterm (<35 weeks and birth weight <2 kg) and full-term infants. | Collection of colostrum, transitional and mature milk from mothers of preterm infants on 5-7, 12-14, 19-21, 26-28, 33-35, 39-42 d pp, and from mothers of term infants on 3-7, 28-35 d pp. Samples were analyzed within 24h. | Spectrophotometry | 30 |
| Huang 2014 (76) | Cross-sectional study | China | Healthy women 18-40 years of age, non-smoking, no alcohol consumption. | Infants <1 month old, singleton. | Collection of milk, by trained staff, 8am-11am, with electronic pump. Stored at -80°C within 4 hours after extraction until analysis. | ICP-MS | 269 |
| **Hunt 2005** (77) | Cohort study | USA | Healthy women 18-36 years, non-smoking, no medication, parity 1-2, mainly Caucasian. | Full-term infants, exclusively breastfed, AGA, birth weight 2.56-4.57. | Collection of mature milk at 1, 2, 3, 4 months pp, during 24h content of one breast not offered to infant, extracted with pump. Stored refrigerated up to 24h. | ICAPS | 36 |
| Itabashi 1999 (78) | Cohort study | Japan |  | Preterm infants (GA 26-33 weeks). | Collection of milk at 1, 2, 3, 4, 5-6, 7-8, and 9-12 weeks pp, by manual expression, both complete breasts, in morning just before lunch. Stored at -40°C until analysis. | ICP-AES | 15 |
| Itriago 1997 (79) | Cohort study | Venezuela | Women >18 years old, living in city of Caracas, 77% from low-middle SES. | Term infants, singleton. | Collection of colostrum, transitional and mature milk at 3, 7 and 21 d pp, 7-10AM, expressed manually. Stored at -15°C until analysis. | ICP-AES | 72 |
| Iwai 2022 (80) | Cross-sectional study | Japan | Healthy women 29-40 y. | Healthy children 3-5 months old. | Collection of milk 3-5 months pp, with manual expresion fore- and hindmilk, in the morning, afternoon and evening. Stored at -80°C until analysis. | ICP-MS/MS | 11 |
| Jarjou 2006 (81)^[[4]](#footnote-4)^ | Randomized controlled trial | Gambia | Women, mean age 27±8 years, mean parity 3, in rural villages, no history of medication condition known to affect calcium or bone metabolism, uncomplicated pregnancy, given 1500 mg/d calcium supplements during pregnancy vs placebo. | Healthy infants, singleton. | Collection of mature milk (2, 13, 52 weeks pp), by hand. Stored at -20°C until analysis. | Colorimetric | 125 |
| Karbasi 2022 (83) | Cross-sectional study | Iran | Healthy women 20-35 y, no acute or chronic illness, categorized into healthy and unhealthy dietary behaviors. | Infants 1-6 months old. | Collection of milk 1-6 months pp, with manual expression, 7-10 AM, before feeding, from one breast. Stored at -80°C until analysis. | Colorimetric | 350 |
| Karra 1986 (84) | Cohort study | USA | Women 21-38 years old, no known disease, with income to enable nutritionally adequate diet, half were consuming vitamin supplements at the beginning of the study. | Infants, not exclusively breasted. | Collection of mature milk monthly 7-25 months pp, foremilk at a single feeding, 7-10AM, expressed manually or with pump. Stored frozen and then at -30°C until analysis. | AAS | 55 |
| Karra 1988 (85) | Cohort study | Egypt, USA | Women, from United States (n=49) 21-38 years old from middle-income SES with some receiving supplements without (n=25) and with (n=24) 25 mg/d zinc, and from Egypt (n=68) 17-34 years, from marginally malnourished, low-income, illiterate, rural population, no supplements. |  | Collection of mature milk at 1, 2, 3, 4, 5, 6 months pp, expressed manually or with pump, after milk letdown, 24h sample or 10 AM-2 PM sample. Stored at -30°C until analysis. | AAS | 117 |
| Kent 1992 (86) | Cohort study | Australia | Women 19-41 years old, vaginal and caesarean delivery. |  | Collection of colostrum milk on day 1-5 pp, from both breasts. | AAS | 12 |
| Khatir Sam 1998 (87) | Cross-sectional study | Sudan | Women, volunteer nurses. |  | Collection of milk, morning samples on 2 consecutive days, sometimes in morning and at noon, expressed with hand pump. | XRF | 20 |
| **Kim 2017** (88) | Cross-sectional study | South Korea | Women 21-45 years, BMI 15-33 (mean 22), 51% took dietary supplements. | Infants, 30-360 days of age, term born 37-42 weeks, normal birth weight 2.5-4.5 kg, exclusively breastfed. | Collection of mature milk from full breast, extracted with pump. Stored at -20°C until analysis. | ICP-OES | 334 |
| Kippler 2009 (89) | Randomized controlled trial | Bangladesh | Women participating in micronutrient supplementation trial. |  | Collection of mature milk at 2 months pp, expressed by hand. Stored at -70°C until analysis. | ICP-MS | 123 |
| Kippler 2012 (90) | Cross-sectional study | Bangladesh | Women, median age 7 (10-90th percentile: 20-36 years old), mean BMI 19, primiparous (n=57) and multiparous (n=43), from rural area, received food supplement and micronutrient supplement (with iron and folic acid or 15 different micronutrients, not containing zinc nor calcium). | Infants, singleton. | Collection of mature milk at 2 months pp, expressed by hand, from one or if insufficient two breasts. Stored at -70°C until analysis. | ICP-MS | 123 |
| Kirksey 1979 (91) | Cross-sectional study | USA | Women 18-31 years, Caucasian, middle SES, primiparous and multiparous, no difficulties with delivery, normal eating habits, some used oral contraception (n=14), with iron supplements (all except one) and other nutrients (n=21). |  | Collection of colostrum, transitional and mature milk 3d, 14 d, 1-3 months, 5-7 months, 1 year pp, before taking supplement, expressed manually. Stored at -20°C until analysis. | AAS | 52 |
| Klein 2017 (92) | Cross-sectional study | Argentina, Namibia, Poland, USA | Women, no indication of mastitis, non-smoking. | Infants 2 weekss-2 years old, singleton. | Collection of mature milk (2 weeks-2 years pp), mid-feed, 8-11:30AM, 2h since last feed, expressed by hand. Stored at -20 or -80°C until analysis. | ICP-MS | 70 |
| Kulski 1981 (93) | Cohort study | Australia | Women, normal delivery. | Term infants, one set of twins. | Collection of colostrum, transitional, and mature milk, by manual expression, from both breasts, 10-12 AM, before feed. Stored at -15° until analysis. | AAS | 18 |
| Laskey 1990 (94)^[[5]](#footnote-5)^ | Cross-sectional study | Gambia, UK | Women, from rural Gambia (n=144), 16-49 years old, parity 1-12, or from UK (n=72), 20-42 years old, parity 1-5. | Infants, 0.5-26 months old. | Collection of mature milk 0.5-26 months pp, from both breasts. | AAS | 216 |
| **Laskey 1998** (96) | Cross-sectional study | UK | Healthy women 20-40 years, mean BMI 25, white, no history of bone disease, not taking medications to affect bone. | Healthy full-term infants, 6-8 weeks old, breastfed for >=3 months. | Collection of mature milk, from both breasts, extracted manually. Stored at -20°C until analysis. | AAS | 47 |
| Lemons 1982 (97) | Cohort study | USA | Women with healthy normal pregnancy, vaginal delivery, with normal pre-pregnancy weight and weight gain. | Preterm (27-37 weeks) and term (39-41 weeks) infants, with AGA. | Collection of milk on 7, 14, 21, 28 d pp and bi-weekly until GA 44 week. Stored at -80°C until analysis. | Modified method of Connerty and Briggswith 1965 | 27 |
| Levi 2018 (98) | Cohort study | Argentina | Women, exposed to varying concentrations of drinking water pollutants. | Infants, exclusively breastfed. | Collection of mature milk (0-3 and 3-6 months pp), expressed by gentile compression. Stored at -20 and then -80°C until analysis. | ICP-MS | 237 |
| Li 1990 (99) | Cross-sectional study | Japan | Healthy women, Japanese, mean age 29±5 years, mean BMI 22±2. | Full-term infants, mean birth weight 3.1±0.4 kg. | Collection of transitional milk (6-9 d pp), in the morning. Stored at -20°C until analysis. | ICP-AES | 27 |
| Li 2008 (100) | Cross-sectional study | China | Healthy women. | Healthy infant. | Collection of colostrum within 3 days pp. | ICP-OES | 16 |
| Li 2016 (101) | Cross-sectional study | Guatemala | Women, mean age 24±6-7 years, mean parity 3±2, with no indication of mastitis. | Infants 5-180 days old, exclusively (56-71%) or predominantly breastfed, 7-16% underweight, 30-45% stunted. | Collection of transitional (5-17 d pp) and mature (18-180 d pp) milk, in the morning, from breast not recently suckled on, by full manual expression. Stored at -30 and then -80°C until analysis. | ICP-MS | 228 |
| Li 2018 (102)^[[6]](#footnote-6)^ | Cross-sectional study | Guatemala | Women, mean age 25, mean BMI 24, primi- (30%) and multiparous (70%), Guatemalan Mam-Mayan. |  | Collection of transitional and mature milk 5-17 d, 18-46 d, 4-6 months pp. Stored at -30°C and then at -80°C. | ICP-MS | 108 |
| **Lin 1998** (104) | Cohort study | China | Healthy women >=20 years, primiparous (n=84) and multiparous (n=127), uncomplicated pregnancy. | Healthy term infants, singleton, growing well. | Collection of colostrum (2-5 d pp), transitional (6-10 d pp) and mature milk (11-365 d pp), expressed with manual pump, one complete breast, second feeding of the day, 9-11AM. Stored at -40°C until analysis. | ICP-AES | 211 |
| Lipsman 1985 (105) | Cohort study | USA | Adolescents 14-20 years, from low-middle income families, White (n=12), Hispanic (n=8), Black (n=3), American Indian (n=2). | Infants, mean birth weight 3.3 kg. | Collection of mature milk monthly 1-10 months pp, from one breast, expressed manually or with manual pump, 9AM-6PM. Stored at -20°C until analysis. | FAAS | 25 |
| Liu 2008 (106) | Cross-sectional study | China | Healthy women 18-36 years, non-smoking, no chronic medication, parity of 1 or 2. |  | Collection of mature milk. | GB/AAS | 99 |
| Liu 2014 (107) | Cohort study | China | Healthy women 20-37 years, no chronic diseases, parity of 1 or 2. |  | Collection of colostrum (3 day postpartum) and mature milk (90 day postpartum). Stored at -40°C until analysis. | AAS | 352 |
| Luo 2010 (108) | Cross-sectional study | China | Healthy women 22.5-34.7 years. |  | Collection of milk, from right breast, expressed by hand or with manual or electric pump. Stored at -20°C until analysis. | FAAS | 758 |
| **Mahdavi 2015** (109) | Randomized controlled trial | Iran | Women, mean age 26-28±5 years, mean BMI 28-29±11, no chronic illness, no gastrointestinal disorder, no supplements, no antibiotics, no corticosteroid, living in urban areas, were randomized to synbiotic supplement (n=27) or placebo (n=27). Data from baseline and placebo group were used. | Full-term infants 3-4 months old, normal birth weight 2.5-4.0 kg, exclusively breastfed, no chronic illness, no gastrointestinal disorder, no supplements, no antibiotics, no corticosteroid. | Collection of mature milk 90-120 d pp, by self-expression, in the morning, before first feeding, after discarding the first 5 ccml. Stored at 4 and then -80°C until analysis. | FAAS | 57 |
| Mandia 2021 (110) | Cohort study | Spain | Women 24-44 y, without chronic disease, not taking nutrient supplements. | Term and preterm infants. | Collection of colostrum (3-4 d pp, only term infants), transitional (7-10 d pp, only term infants) and mature milk (from both preterm and term infants), by manual expression. Stored -20°C until analysis. | ICP-MS | 170 |
| Maru 2013 (111) | Cross-sectional study | Ethiopia | Healthy women 18-35 years old, no supplementation except from iron/folic acid supplement, in rural and urban area. | Healthy full-term infants. | Collection of colostrum milk (within 4 d pp), by manual expression. Stored at-20°C until analysis. | FAAS | 45 |
| Mastroeni 2006 (112) | Cohort study | Brazil | Women, mean age 23±5 years, mean parity 2.7±1.5, vaginal (n=27) and caesearan (n=4) delivery. | Term infants, mean birth weight 3.1±0.5 kg. | Collection of colostrum (2 d pp) and mature milk (2 months pp), by manual expression. Stored at -20°C until analysis. | ICAP-AES | 43 |
| Mataloun 2000 (113) | Case-control study | Brazil | Healthy women, good nutritional health, not receiving any medication that could interfere with lactation. | Healthy term infants, singleton, AGA (n=41) or SGA (n=30). | Collection of colostrum (3 d pp), transitional (15 d pp), and mature (30 d pp) milk, from both complete breasts, by manual expression, 8-11 AM. Stored at -20°C until analysis. | AAS | 71 |
| Minato 2019 (114) | Cohort study | Japan | Healthy women, mean age 34 years, no thyroid disease, high blood pressure or diabetes, no dietary restriction. | Infants, singleton, at 1 and 3 months old. | Collection of mature milk at 1 and 3 months pp. Stored at <5°C and then -80°C until analysis. | ICPE | 129 |
| Moron de Salim 2016 (43) | Cross-sectional study | Venezuela | Apparently healthy women 20-35 years, mean BMI 25, no pathology before or during pregnancy, no mastitis, no fever, no mammary abscess, no diabetes, no hypertension, no renal insufficiency, no cancer, no chronic neuropathy, non-smoking, no alcohol, drugs or medication that could interfere with calcium metabolism. | Infants, GA 36-41 weeks, singleton, with no fetal pathology. | Collection colostrum, expressed manually. Stored at -70°C until analysis. | FAAS | 50 |
| Motil 1997b (115) | Cohort study | USA | Healthy women, adolescents (n=11) and adults (n=11), well-nourished, non-smoking, no chronic medications, parity ≤3, uncomplicated pregnancy. | Term infant, AGA. | Collection of mature milk 6, 12, 18, 24 weeks pp. Stored at -70°C until analysis. | AAS | 22 |
| Nagra 1989 (116) | Cohort study | Pakistan | Women 27-35 years, medium SES. |  | Collection of mature milk every month 1, 2, 3, 4, 5, 6, 7, 8, 9, 10 , 11, 12 months pp, fore- and hindmilk, 9-11 AM, after eating breakfast. | AAS | 20 |
| **Neville 1984b** (117) | Cross-sectional study | USA | Women 18-40 years, primiparous and multiparous, White and Black (n=1). | Healthy full-term infants, exclusively breastfed. | Collection of mature milk 33-210 d pp, expressed with pump. Stored at -70°C until analysis. | Colorimetric | 12 |
| Nickkho-Amiry 2008 (118) | Cross-sectional study | UK | Women 22-42 years, from veiled of Arab origin (n=14) and white Caucasian (n=10), not taking vitamin D supplements. | Infants, exclusively breastfed. | Collection of mature milk 9-13 weeks pp, expressed by hand, at the beginning of feeding, in morning or afternoon. | Colorimetric | 24 |
| Noh 2021 (119) | Cross-sectional study | South Korea | Women ≥20 y, no mental illness. | Healthy term infant, birthweight >2.5 kg. | Collection of milk 7 d pp, after first feeding of the morning. Stored at -20°C until analysis. | ICP-MS | 40 |
| Oliveira 2019 (120) | Cross-sectional study | Brazil | Women 17-44 years, BMI 18-38. | Infants, GA 27-42 weeks. | Mature milk >15 d pp from milk bank. Pasteurized and stored at -20°C for 24h. | FAAS | 50 |
| Ortega 1998 (121) | Cohort study | Spain | Healthy women 18-35 years old, no diabetes, no pre-eclampsia, living in urban area, with calcium intake </>=1100 mg/d. | Infants, singleton, normal birth weight >=2.5 kg. | Collection of transitional milk 13-14 d pp and mature milk 40 d pp, 10-11AM, by manual expression, fore- and hindmilk. | Colorimetric | 57 |
| Parr 1991 (122) | Cross-sectional study | Guatemala, Hungary, Nigeria, Philippines, Sweden, Democratic Republic of the Congo | Women from different countries, traditions, SES and nutritional backgrounds. | Infants, 3 months old. | Collection of mature milk (3 months pp) of one complete breast, around noon, approximately 4h after the previous feed, extracted with pump. Frozen at -11°C until analysis. | AAS | 330 |
| **Perrin 2017** (123) | Cohort study | USA |  | Healthy term infant 9-11 months old. | Collection of mature milk monthly 11-17 months pp, one complete breast, first or second feeding of the morning. Stored in freezer and then -80°C until analysis. | ICP-OES | 19 |
| Picciano 1981 (124) | Cohort study | USA | Women >=20 years (mean age 25 years), 69% taking vitamin-mineral supplements. | Full-term infants, birth weight 2.8-5.2 kg, exclusively breastfed. | Collection of mature milk at 1, 2, 3 months pp, expressed manually or with manual pump. Stored frozen until analysis. | AAS | 26 |
| Pietrzak-Fiecko 2020 (125) | Cross-sectional study | Poland | Women 21-34 years. |  | Collection of mature milk 3-4 months pp, hindmilk, with pump, one breast. Stored at -20°C until analysis. | FAAS | 18 |
| Prentice 1995 (126)^[[7]](#footnote-7)^ | Randomized controlled trial | Gambia | Healthy women 16-41 years, parity 1-13, living in rural areas, randomized assigned to calcium supplement (1000 mg 5x/week) or placebo. | Infants, singleton. | Collection of mature milk at 6, 13, 19, 26, 39, 52, 65, 78 weeks pp, expressed manually, from each breast. Stored at -20°C until analysis. | Spectrophotometry | 60 |
| Qian 2002 (129) | Cross-sectional study | China | Healthy women 18-36 years, non-smoking, no chronic medication, parity of 1-2. |  | Collection of transitional milk. | FAES | 120 |
| Qian 2010 (130) | Cross-sectional study | China | Healthy women 22-36 years, primiparous, living in residential areas from urban and suburban areas, no chronic illness, consuming normal varied diets. | Healthy full-term infants. | Collection of transitional milk 8-10 d pp, foremilk, 9-11 AM, by manual expression. Stored at -20°C until analysis. | AAS | 120 |
| Qian 2022 (131) | Cohort study | China | Healthy women 20-42 years, no chronic diseases, 449 out of 678 had parity of 1, 405 out of 678 had vaginal delivery, |  | Collection of colostrum (0-5 day postpartum) and transitional milk (10-14 day postpatum), and mature milk (40-45 d, 200-240d, and 300-400d postpartum). Stored at -80°C until analysis. | ICP-MS | 678 |
| Queiroz Bortolozo 2004 (132) | Cross-sectional study | Brazil | Milk donors of term and pre-term infants. | Term (n=20) and preterm (<=36 weeks, n=10) infants. | Milk from milk bank, pasteurized. Collection of colostrum and mature milk. | AAS | 30 |
| Rana 1990 (133) | Cross-sectional study | Pakistan | Women 19-32 years, wives of army personnel from low-income group, BMI 13.5-23.7, parity 1-4. |  | Collection of milk samples 2-6 d pp. | AAS | 26 |
| Rodriguez Rodriguez 2002 (134) | Cohort study | Spain | Healthy women 21-35 years. |  | Collection of mature milk 2 weeks-5 months pp, from fore- and hindmilk, representative of different times of the day. | AAS | 11 |
| Rona 2008 (135) | Cross-sectional study | Brazil | Healthy milk donors. | Infants 16-180 days old, term (75%, GA >=39 weeks) and preterm (25%, GA 34-38 weeks). | Collection of mature milk (16-180 d pp), from milk donor bank, expressed manually. Stored immediately at -20°C. | Colorimetric | 37 |
| Sabatier 2019 (136) | Cohort study | Switzerland | Healthy women >18 years, no diabetes, no alcohol or drugs consumption. | Term (<37 weeks) and preterm (28-32 weeks) infants. | Collection of transitional and mature milk at 1, 2, 3, 4, 5, 6, 7, 8 weeks pp for preterm and term infants, and at 10, 12 , 14, 16 weeks pp for preterm infants, from a full single breast, between 6-12 AM, with electric pump. Stored at -18 and then -80°C until analysis. | ICP-MS | 61 |
| Salah 2016 (137) | Cohort study | Sudan | Healthy women 18-38 years, 2 weeks after fasting month of Ramadan, non-smoking, no medication, no supplements, no chronic diseases. | Infants 3-6 months old. | Collection of mature milk 3-6 months pp, by manual expression, from both breasts, immediately after first feeding of the day, 9-11AM. Stored at -21°C until analysis. | FAES | 24 |
| Samuel 2020 (138) | Cohort study | France, Italy, Norway, Portugal, Romania, Spain, Sweden | Women 18-40 years, BMI 19-29, no condition or medication that contraindicates breastfeeding, with (n=108) or without (n=197) subclinical mastitis. | Infants, mean GA 39-40 ±1 weeks, mean birth weight 3.2-4±0.4-5 kg. | Collection of colostrum and mature milk at 2, 17, 30, 60, 90, 120 days pp, with an electric pump, 9 AM-1 PM, from one complete breast. Stored at -18 and then -80°C until analysis. | ICP-MS | 331 |
| Sann 1981 (139) | Cross-sectional study | France |  | Infants, term (n=61, GA 38-41 weeks) and preterm (n=41, GA 26-35 weeks). | Collection of milk <=6 d, 7-14 d, >=15 d pp, expressed by mechanical or manual methods, 4-6x per day. Stored at 4°C and pasteurized. | AAS | 102 |
| Schanler 1980 (140) | Cross-sectional study | USA | Mothers of preterm infants and milk donors of term infants. | Preterm and term infants. | Collection of milk from mothers of preterm infants 1-98 d pp of from donors of term infants 1-8 month pp, expressed manually or by breast pump, from each feeding and each day. | AAS | 29 |
| Seki 1997 (141) | Cohort study | Japan | Women, primi- and multiparous, uncomplicated pregnancies, no medication, vaginal and cesarean deliveries. | Infants, born 37-39 weeks gestational age, birthweight 2.85-3.45 kg. | Collection of milk 1-30 d pp, at different times of the day. Stored at -20°C until analysis. | AAS | 21 |
| **Shehadeh 2006** (142) | Cross-sectional study | Israel | Healthy women. | Healthy term infants, birth weight AGA, exclusively breastfed until 3 months. | Collection of milk, from one breast, expressed with pump, 3 min after start of feeding and until complete emptying of breast, in morning 8-10AM. Stored at -20°C until analysis. | AAS | 84 |
| Shi 2011 (143) | Cohort study | China | Healthy women, mean age 28 years, mainly primigravida, non-vegetarian. | Healthy full-term infants. | Collection of colostrum, transitional, and mature milk, from both breasts, after lunch (2nd feed of the day), extracted with pump. Stored at -20°C until analysis. | AAS | 80 |
| Silva 1997 (144) | Cross-sectional study | Brazil | Women, from city of Brasilia. |  | Collection of milk at different stages of lactation, by manual expression. Stored at -20°C until analysis. | ICP-AES | 203 |
| Sunaric 2017 (145) | Cohort study | Serbia | Healthy women 30-44 years, from urban area, well-nourished, non-smoking, non-vegetarian, no vitamin supplements. | Term infants. | Collection of colostrum (1-7d pp), transitional (8-14 d pp) and mature (after 15 d pp) milk. Stored at -20°C until analysis. | Colorimetric | 67 |
| Szukalska 2021 (146) | Case-control study | Poland | Healthy women 18-40 y, non-smoking, exposed to second-hand smoke or smoking. |  | Collection of colostrum (0-2 d pp) and mature milk (23-37 d pp), by electronic pump. Stored at -80°C until analysis. | ICP-MS | 150 |
| **Taravati Javad 2018** (147) | Cross-sectional study | Iran | Healthy women 19-42 years, no supplements, non-vegetarian, no medication to enhance milk production. | Full-term infants, normal birth weight, exclusively breastfed, no chronic disease. | Collection of mature milk at 1, 2, 6, 7, 12 months pp, in the morning, more than 1h since last feed, expressed with manual pump. Stored at 4 and the -20°C until analysis. | ICP-MS | 100 |
| Terheggen 1965 (148) | Cohort study | Germany |  |  | Collection of colostrum (1 d pp), transitional (6 d pp) and mature milk, first morning feeding, fore- and hindmilk. | FAAS | 39 |
| **Thacher 2006** (149) | Case-control study | Nigeria | Women, mean age 28-30±5-6, mean parity 4-5. | Infants, mean age 40-41 weeks old, mean gestational age 40 weeks (all term infants, except 1 infant 36 weeks GA), with rickets (n=35) and without (n=105). | Collection of mature milk, from right breast. Stored at -20°C and then -80°C until analysis. | Colorimetric | 140 |
| Toddywalla 1977 (150) | Controlled trial | India | Healthy women 20-30 years old, fully established lactation, previous history of successful lactation, similar socioeconomic background, assigned to conventional contraceptives (control) or taking a low-dose combination pill, a low-dose progestational compound, or a 3 or 6 monthly injection. Data from the control group were used. |  | Collection of mature milk, from both breasts, 3-3.5 since last feed. | Kraemer and Tisdel method as modified by Clark and Collip | 36 |
| Umeta 2003 (151) | Cross-sectional study | Ethiopia | Women, mean age 26-27±0.4-6 years, BMI 20±0.1-0.2, in rural area. | Infants 5-11 months old, not stunted. | Collection of mature milk, from right breast 1h after last feeding. Stored -20°C until analysis. | AAS | 195 |
| Van Steenbergen 1981 (152) | Cross-sectional study | Kenya | Women, in rural area, during lean and post-harvest seasons. | Infants 0-24 months old. | Collection of milk 0-24 months pp, around 11 AM, after 4 h without feeding, expressed manually. Stored at -20°C until analysis. | AAS | 85 |
| Vanderja 2002 (153)^[[8]](#footnote-8)^ | Cross-sectional study | Nigeria | Women 15-45 years, from Fulani ethnicity, parity 1-10, BMI 14.5-24. | Infants 2-24 weeks old (mean age 11 weeks). | Collection of mature milk 2-24 weeks pp, by manual expression, mid-feed. Stored at 20°C and then frozen until analysis. | ICP-OES | 34 |
| **Vaughan 1979** (155) | Cohort study | USA | Women, Caucasian, good or excellent nutritional status, no complication during pregnancy or delivery, primiparous (n=22) and multiparous (n=16). | Full-term infants. | Collection of mature milk monthly 1-31 months pp, expressed by hand, over 3-5 days in morning, afternoon and evening feeds, randomly during feed. Stored frozen until analysis. | AAS | 38 |
| Vitolo 2004 (156) | Cross-sectional study | Brazil | Healthy adolescents and women 14-39 years, non-smoking, no medication, no vitamin supplements, normal childbirth and post-partum, BMI >19, from low and high SES. | Infants 1-3 months old, exclusively breastfed, singleton. | Collection of mature milk 30-90 d pp, by manual expression, both breasts for several days at different times during afternoon. Stored at -20°C until analysis. | FAAS | 90 |
| Wang 2002 (157) | Cohort study | China | Healthy women 24-30 years of age, no heart, liver, kidney, or pulmonary-related diseases, no medication history. | Healthy full-term infants. | Collection of colostrum (2-5 d pp) and mature milk (30-35 d pp) before feeding by hand. | FAAS | 31 |
| Wang 2007 (158) | Cross-sectional study | China | Healthy women 18-38 years with no recent supplementation of calcium, magnesium, manganese, or iron. |  | Collection of mature milk 38-510 d pp, from one breast, in the morning, 7-10 AM. Stored at -70°C until analysis. | AAS | 55 |
| Wei 2020 (159) | Cross-sectional study | China | Healthy women 20-40 years, from different geographical regions of China. |  | Collection of colostrum (3-6 d pp), transitional (10-13 d pp) and mature (21-25 d pp), from second feeding in the morning (9 AM). Stored -20°C and the -80°C until analysis. | GB/AAS | 175 |
| Yamawaki 2005 (160) | Cross-sectional study | Japan | Women <40 years, non-smoking, no vitamin supplements. | Infants, birth weight >=2.5 kg, no symptoms of atopy. | Collection of colostrum/transitional/mature milk 1-365 d pp, mid-feed. Stored at -20 and then -40°C until analysis. | ICP-AES | 1197 |
| Yoneyama 1997 (161) | Cross-sectional study | Japan | Healthy women 21-42 years, no disease that affects bone metabolism, non-smoking, parity 1-3. |  | Collection of mature milk 21-590 d pp, by manual expression. | AAS | 105 |
| Yoshinaga 1991 (162) | Cross-sectional study | Japan | Women, mean age 28±5 years, mean BMI 22±2. | Infants, mean birth weight 3.1±0.4 kg. | Collection of transitional (5-9 d pp) milk, by manual expression. Stored at -20°C until analysis. | ICP-AES | 51 |
| Zhao 2011 (163) | Cross-sectional study | China | Healthy women with mean age of 26.36, primigravida, non-smoking, no medication history. | Infants, exclusively breastfed. | Collection of milk samples, Stored at -20°C until analysis. | GB/T | 39 |
| **Zhao 2014** (164) | Cross-sectional study | China | Healthy women 18-45 years old, living in 3 cities of China, without diabetes, hypertension or other chronic or acute disease, no nipple or lacteal gland disease, no use of hormone, no postpartum depression or other mental disease, 96% Han ethnicity, mean BMI 23, vaginal and cesarean delivery. | Health full-term infants, singleton. | Collection of milk at 5-11, 12-30, 31-60, 61-120, 121-240 d pp, from one complete breast, 9-11 AM, with electric pump. Stored at -80°C until analysis. | ICP-MS | 444 |
| Zimmerman 2009 (165) | Cohort study | Israel | Healthy women 24-38 years, Jewish women who were planning a religious fast. Data from before fast were used. | Healthy infants 1-6 months old, exclusively breastfed. | Collection of mature milk 1-6 months pp, 2h before fast, foremilk, expressed by hand, manual pump or electrical pump. Stored at -20°C until analysis. | Colorimetric | 48 |

Note : Studies included in selection of key studies in **bold**.

# ADDITIONAL FILE 3. Characteristics of studies included for breast milk ZINC concentration

| **ID [Reference]** | **Study design** | **Country** | **Mother description** | **Child description** | **Milk description** | **Analytical method** | **Sample size** |
| --- | --- | --- | --- | --- | --- | --- | --- |
| Abdulrazzaq 2008 (166) | Cross-sectional study | United Arab Emirates | Women 18-50 years, Unites Arab Emirates (35%) and other nationalities, 1% smoking. | Infants 0-80 weeks old. | Collection of colostrum/transitional/mature milk 0-80 weeks pp, expressed with pump, both complete breasts. Stored at -30°C until analysis. | ICP-MS | 205 |
| Adesiyan 2011 (2) | Case-control study | Nigeria | Women 26-46 years, taking or not hormonal oral or injectable contraceptives. |  | Collection of mature milk, approximately 8 months pp, manually. Stored at -20°C until analysis. | AAS | 182 |
| Ahmed 2004 (167) | Cross-sectional study | Bangladesh | Women 16-40 years (mean age 23 years, monthly income 106 USD, BMI 22, and parity 3.6). | Infants of 2 days old. | Collection of colostrum (2 days pp), extracted by hand. | AAS | 105 |
| **Al-Awadi 2000** (168)^[[9]](#footnote-9)^ | Cross-sectional study | Kuwait | Healthy women 25-40 years, Kuwaiti and non-Kuwaiti nationality, from middle/upper-middle income class, no history of medical or breast-feeding problems, not taking any kind of mineral or trace-element supplementation. | Healthy full-term infants, delivered normally, from 2 weeks to 18 months old, no history of medical or breast-feeding problems, not taking any kind of mineral or trace-element supplementation. | Collection of mature milk (2 weeks - 18 months pp) before first feeding in the morning, extracted by hand. | AAS | 34 |
| Al-Terehi 2015 (170) | Cross-sectional study | Iraq | Women 20-42 years. |  | Collection of milk 1-51 weeks pp, with manual pump. Stored at -20°C until analysis. | AAS | 70 |
| Alam 2015 (171) | Cross-sectional study | USA | Healthy women 18-40 years, non-smoking. | Singleton full-term infants, 4 months old. | Complete collection of mature milk (4 months pp) from one breast in the morning. | AAS | 54 |
| Almeida 2008 (172) | Cohort study | Portugal | Healthy women 18-40 years (mean age: 28.3 years), primiparous (48%) and multiparous. | Full-term (86%) and pre-term infants. | Collection of colostrum (2 days pp) and mature milk (1 month pp), extracted by hand. Stored at 4°C until analysis. | ICP-MS | 44 |
| Alves Peixoto 2019 (173) | Cross-sectional study | Brazil | Women, mean age 24-25±5-6 years, primiparous and multiparous. | Infants, preterm and term. | Collection of milk. Stored at -20°C until analysis. | ICP-MS | 156 |
| Anastacio 2004 (5) | Cross-sectional study | Brazil | Healthy women 19-43 years (mean age: 28), BMI 18.3-34, exclusively or predominantly breast-feeding, no history of occupational or accidental lead exposure and no complications during pregnancy or lactation. |  | Collection of mature milk (20-102 days postpartum, mean 56 days postpartum), extracted with manual pump. Stored at 4°C up to 2h after collection and frozen at -70°C until analysis. | ICP-OES | 38 |
| Anderson 1992a (174) | Cohort study | USA |  |  | Collection of several milk samples, up to 5 months pp, extracted with breast pump or cup. | ICAPS | 7 |
| Anderson 1993 (175) | Cohort study | USA | Women 20-30 years, graduates or wives of graduates, adequate diets, no vegetarian, from USA, Libya, Peru, Portugal, and Rwanda. | Healthy full-term infants, exclusively breastfed. | Collection of colostrum/transitional/mature milk from 1 to 164 d pp, with breast pump or plastic breast shield, from morning feed. Frozen until analysis. | ICAPS | 6 |
| Aquilio 1996 (176) | Cohort study | Italy | Women 21-29 years. | Infants, term (n=8, GA 40±1 weeks, mean birth weight 3.2±0.3 kg) and preterm (n=6, GA 35±2 weeks, mean birth weight 2.1±0.2 kg), AGA. | Collection of colostrum, transitional and mature milk from 0 to 21 d pp, by manual expression or pump. Frozen until analysis. | ICP-AES | 14 |
| Arnaud 1992 (177) | Cross-sectional study | France | Healthy women 19-39 years, apparently well fed, no albumin or glucose in urine, hemoglobin 11.5 g/dL at delivery, uncomplicated pregnancy, primiparous (n=45) and multiparous, urban (n=68) and rural (n=15) areas, mainly Caucasian (n=61), mainly middle income (n=56), pre-pregnancy BMI 17-31. | Healthy full-term infants, singleton, growing well, GA 36-43 seeks, birth weight 2.4-4.3 kg. | Collection of milk during first 2 months pp, expressed by hand, before morning feeding, 9-11 AM. Stored at -18°C until analysis. | ETAAS | 83 |
| Arnaud 1993 (178) | Randomized controlled trial | Niger | Healthy women 17-45 years, parity 0-14, multiparous (n=194), uncomplicated pregnancy and delivery, randomized to iron supplement (100 mg/d) or placebo. | Infants, birth weight 1.5-4.1 kg (mean 3.0-1±0.4-5 kg), mean Apgar score 9. | Collection of transitional and mature milk at 5 d, 3 and 6 months pp, one complete breast. Stored at -20°C until analysis. | FAAS | 197 |
| Arnaud 1995 (179) | Cohort study | France | Healthy women 19-39 years, apparently well-nourished, mainly Caucasian (n=60), mainly middle-income (n=64), mean parity 2 (range: 1-8), primiparous (n=46) and multiparous (n=36), mean pre-pregnancy BMI 22 (range 17-31), no history of serious disease, uncomplicated pregnancy. | Healthy full-term infants, singleton, mean birth weight 3.3 kg (range: 2.6-4.3 kg). | Collection of colostrum and transitional milk 1-7 d pp, by hand expression, foremilk, morning feed 9-11 AM. Stored at -20°C until analysis. | FAAS | 82 |
| Arver 1984 (9) | Cohort study | Sweden | Healthy women. |  | Collection of colostrum/transitional milk 0-2 and 4-6 d pp. Stored at 4°C and then frozen until analysis. | AAS | 75 |
| Astolfi 2018 (10) | Cross-sectional study | Italy | Women, living in Rome. |  | Collection of mature milk, 4-6 weeks pp, extracted with manual pump or passive breast milk sampler. Stored at 4°C and then -20°C. | ICP-MS | 20 |
| Atinmo 1982 (180) | Cohort study | Nigeria | Women, predominantly from low SES. | Infants, preterm (n=15, GA 28-34 weeks) and term (n=20, GA 37-31 weeks), singleton. | Collection of colostrum, transitional, and mature milk at 2-7, 8-14 d, 2 months pp, by manual expression, at noon, before feeding. Stored at -20°C until analysis. | AAS | 35 |
| Aumeistere 2018 (181) | Cross-sectional study | Latvia | Healthy women, mean age 31 ±4 years, living in Latvia, breastfeeding only one child. | Healthy infants, birth weight >2.5 kg, singleton, exclusively or partially breastfed, 1.5-16.0 months old. | Collection of mature milk, a pooled sample representative of a 24h period with samples from morning, mid-day and evening feeds, hindmilk only. Stored at 4°C and then -18°C until analysis. | ICP-MS | 62 |
| Bamgbose 2012 (182) | Cohort study | Nigeria | Women 17-38 years old (mean age 27), primiparous (23%) and multiparous (77%), 22% no education, 33% only primary education. |  | Collection of transitional/mature milk 10-336 d pp, by manual expression, before feed. Stored at -15°C until analysis. | AAS | 86 |
| Benemariya 1995 (183)^[[10]](#footnote-10)^ | Cohort study | Burundi | Apparently healthy women, mean age 32±1 years, primiparous (n=2) and multiparous (n=3), middle class, uncomplicated pregnancy. |  | Collection of colostrum, transitional and mature milk at 2-4 d, 5-14 d, 1-10 months pp, by manual expression, 3x per day, fore- and hindmilk. Stored in refrigerator for 24h and then at -20°C until analysis. | FAAS | 5 |
| Biego 1998 (185) | Cross-sectional study | France |  |  | Mature milk samples from milk bank. | ICP-MS | 17 |
| **Bilston-John 2021b** (17) | Cohort study | Australia | Women, non-smoking, high SES. | Healthy term infants, singleton, exclusively breastfed up to 5 months, continued breastfeeding up to 12 months. | Collection of milk samples over a 24h period, with manual expression, before and after feed. Storage at 20°C until analysis. | ICP-MS | 11 |
| Bjorklund 2012 (18) | Cross-sectional study | Sweden | Women, mean age 29±4 years, BMI 23±4, primiparous, from a random sample. |  | Collection of mature milk sample during 7 days (during 3rd week postpartum, 14-21 days pp), sampling from both beginning and end of breastfeeding session, extracted with manual pump or passive sampler extraction. Stored in freezers until analysis. | ICP-MS | 60 |
| Bloom 1978 (19) | Cross-sectional study | Australia | Women 23-27 years. |  | Stored in refrigerator until analysis. | AAS | 3 |
| Bocca 2000 (20) | Cross-sectional study | Italy | Women 19-40 years old, Italian. |  |  | ICP-AES | 60 |
| Bolognini Pereira 2013 (21) | Cohort study | Brazil | Adolescents <18 years old, no infectious disease, not taking nutritional supplements, no medication. | Infants, exclusively or predominantly breastfed. | Collection of mature milk, extracted manually at 6, 10, 14 weeks pp, from breast not sucked in last feeding, 9-10AM. | AAS | 51 |
| Bosscher 2001 (22) | Cross-sectional study | Belgium |  |  | Pooled mature milk sample. | FAAS | 4 |
| Bosscher 2003 (23) | Cross-sectional study | Belgium |  |  | Pooled mature milk sample. | FAAS | 4 |
| Butte 1984 (25) | Cohort study | USA | Women 20-35 years, parity <=2, max 2 servings/d of carbonated beverages, coffee, tea and alcohol, no routine medication, no steroid contraceptives, good health (all, except n=2 with hypertension in pregnancy), taking vitamin-mineral tablets (all, except n=2), non- and smoking (n=4). | Term (>=37 weeks) and preterm (<37 weeks) infants, no congenital or acquired disease, exclusively breastfed (all, except a few cases who were introduced foods at 6-8 weeks and some preterm infants also given formula). | Collection of mature milk at the end of week 2, 4, 6, 8, 10, 12 pp, 8-12 AM, at least 2h after feeding, with breast pump. Stored at 4 and then -20°C. | AAS | 21 |
| **Butte 1987a** (26)^[[11]](#footnote-11)^ | Cohort study | USA | Healthy women 18-36 years, non-smoking, no long-term medication, parity 1-2, middle-upper SES, White (n=41), Hispanic (n=2), Asian (n=1) and West Indian (n=1), vaginal (84%) and cesarian (16%) delivery. | Heathy full-term AGA infants, exclusively breast-fed, at 1, 2, 3, and 4 months pp, birth weight 2.6-4.6 kg. | Collection of a complete milk sample from the breast opposite than offered to the infant, with electric pump. Stored at -20°C until analysis. | AAS | 45 |
| Butte 1987b (30) | Case-control study | USA | Moderately controlled type 1 diabetic women, otherwise healthy, non-smoking, not taking oral contraceptives. Healthy non-diabetic women as controls. | Healthy infants, no congenital abnormalities, 90 days old, exclusively breastfed. | Collection of 24h mature milk at 3 months pp, alternatively breast opposite to the one feeding the infant, complete breast, extracted with electric pump. | AAS | 5 |
| **Butts 2018** (31) | Cross-sectional study | New Zealand | Healthy women 19-42 years, BMI 20-39, breastfeeding, of different ethnicities (10% Asian, 22% Maori & Pacific Island or 68% European), not dieting, no health disorder. | Full-term infants 6-8 weeks old, exclusively or primarily breastfed, without neonatal care, birth weight 2.4-4.6 kg. | Collection of 3 mature milk samples over 1 week, before first feed of the day, extracted by hand or breast pump. Stored at -18°C, thawed and then stored again at -80°C until analysis. | ICP-MS | 78 |
| **Bzikowska-Jura 2021** (186) | Cross-sectional study | Poland | Healthy women, ≥18 y, no gestational diabetes, no smoking, sufficient milk supply. | Term infants 4-6 weeks old, exclusively breastfed, singleton, birthweight ≥2.5 kg. | Collection of mature milk 6-8 weeks pp. | ICP-MS | 32 |
| Cardoso 2014 (187) | Cross-sectional study | Brazil | Women, living in Coceicao das Alagoas. |  | Collection of duplicated milk samples during 1st month pp. Stored at -20°C until analysis. | ICP-MS | 58 |
| **Carias 1997** (36) | Cohort study | Venezuela | Healthy women 18-35 years, living in metropolitan Caracas, no chronic disease, no anemia, no signs of malnutrition or vitamin deficiency, mainly from low income families and deficitary sanitary conditions. | Term infants. | Collection of colostrum (48-54h) and mature milk samples (at 1, 3, 6 months pp), extracted manually from breast opposite to the one offered to the infant, in morning and evening. Stored at -20°C until analysis. | AAS | 45 |
| Casey 1985 (188) | Cohort study | USA | Women 26-39 years, white, middle-class, apparently well-nourished, average parity 2.5, who had previously breastfed at least one infant, with uncomplicated pregnancy. | Healthy full-term infants, singleton, birth weight 2.7-4.1 kg. | Collection of colostrum, transitional and mature milk samples during the first 14 days and then weekly until 1 month, extraction of colostrum with electric pump and then extraction by hand, mid-feed at mid-morning feed, from both breasts. Stored in the refrigerator and then -70°C until analysis. | AAS | 11 |
| **Casey 1989** (189) | Cohort study | USA | Healthy women, non-smoking, normal pregnancy weight, uneventful pregnancy, no problems with breastfeeding. | Healthy full-term infants, singleton. | Collection of colostrum, transitional and mature milk, weekly up to 1 month and then monthly up to 18 months postpartum or to weaning, during midmorning feed, midfeed from both breasts, extracted with pump for the first 1-2d and then by hand. | FAAS | 13 |
| Castro 2014 (37) | Cohort study | Chile | Women 15-40 years old, mean BMI 27-28 (range 21-39), primi- and multiparous, living in major mine tailing deposition (n=24) or control area (n=11). | Term singleton infants, at least 90% exclusively breastfed, at 2-4 months and 4-6 months of age. | Collection of mature milk samples at 2-4 months pp, in the morning. Stored at -80°C until analysis. | ICP-MS | 35 |
| Cebi 2022 (190) | Cross-sectional study | Turkey | Women, 21-39 years old, non-smoking. | Infants 1 week-6 months old. | Collection of milk 1 week-6 months pp. Stored at -80°C until analysis. | ICP-MS | 50 |
| Chen 2007 (39) | Cross-sectional study | China | Healthy women 18-36 years, non-smoking, no chronic medication, parity of 1 or 2. |  | Collection of mature milk at 1, 2, 3, 4, 5, 6 months pp. | ICP-AES | 240 |
| **Chierici 1999** (191) | Controlled trial | Italy | Healthy women, good SES, no history of thyroid disease, non-smoking, non-vegetarian, normal weight gain during pregnancy, uncomplicated pregnancy, labor and delivery, given or not a nutritional supplement (with zinc, copper, iodine). | Full-term healthy infants, singleton, AGA, exclusively breastfed. | Collection of milk at 3, 30, 90 d pp, extracted with breast pump, before start of feeding. Stored at -30°C until analysis. | ICP-MS | 22 |
| Cinar 2011 (192) | Cross-sectional study | Turkey | Women, in industrial, urbanized and rural areas. |  | Collection of mature milk (4 week pp), by hand. Stored at -20°C until analysis. | ICP-OES | 90 |
| Cisse 2002 (193) | Controlled trial | Senegal | Women, mean age 27±6 years, mean BMI 22-23±3-4, supplemented (with maize or millet supplement with calcium, zinc, iron, niacin, folic acid and vitamins A, C, D1, B1, B2, B12) and non-supplemented, age-matched and similar socioeconomic conditions, living in poor suburban areas of Dakar. |  | Collection of transitional milk (14d pp), from each breast, mid-morning and mid-afternoon, extracted by hand. | AAS | 133 |
| Citrakesumasari 2019 (40) | Cross-sectional study | Indonesia |  | Full-term infants 2-6 weeks old, breastfed, normal weight (n=31) or low birth weight (n=6). | Collection of mature milk (2-6 weeks pp), manually and then with pump. Stored in a freezer until analysis. | AAS | 37 |
| Da Costa 2002 (194) | Cross-sectional study | Brazil | Women 20-38 years, mean parity 1.8, mean BMI 21. | Full-term infants. | Collection of colostrum sample (0-4d pp), during a regular feeding of the infant, from both breasts between 10 AM and 2 PM, extracted by hand using disposable gloves. Stored at -20°C until analysis. | XRF | 48 |
| Da Costa 2003 (195) | Cross-sectional study | Brazil | Women >=21 years, with a minimum of 2y inter-parturition, not taking supplements. | Infants, preterm (32-36 weeks) and full-term (37-31 weeks). | Collection of colostrum (1-7d pp), 10AM-2PM, extracted by hand. Stored at -20°C until analysis. | ICP-MS | 86 |
| Dagnelie 1992 (42) | Cross-sectional study | Netherlands | Healthy women, on macrobiotic or omnivorous diet. | Infants, 2.0-3.3 and 9.0-13.0 months pp. | Collection of mature milk, on 2 consecutive days, from opposite breast after finishing first breast. Stored at -4°C and then -20°C. | AAS | 31 |
| Dang 1983 (196) | Case-control study | India | Women, no taken any hematinic during or after pregnancy. | Full-term infants, normal (n=16), with neonatal obstructive jaundice (n=6) or neurological abnormality (n=9). | Collection of mature milk (1.5-3 months pp), after drink a cup of tea before 6 AM, collected at 10-11 AM, no feeding since 4 AM, after 4 drops of milk discarded. Stored frozen until analysis. | INAA | 31 |
| Dang 1984 (197) | Cross-sectional study | India | Women, from low (n=17) and middle (n=14) income families. |  | Collection of colostrum/transitional and mature milk at 3-5 d and 4-6 weeks pp, 11:00-11:30 AM. Stored frozen until analysis. | INAA | 31 |
| Dang 1985 (198) | Cross-sectional study | India | Women, economically poor, from urban (n=76) and tribal (n=10) areas, including undernourished women (n=26), no hematinics during or after pregnancy. |  | Collection of mature milk (1 month - 2.5 years), after drink a cup of tea before 6 AM, collected at 10-11 AM, no feeding since 6 AM, after 4 drops of milk discarded. Stored frozen until analysis. | INAA | 86 |
| De Figueiredo 2010 (199) | Controlled trial | Brazil | Women 14-36 years (mean age 24-25±5-6 years), given 0 (n=20) or 50 (n=18) mg/d zinc supplement. | Preterm infants (GA <= 34 weeks), birth weight 1.3-1.9 kg, exclusively breastfed, breast milk intake >=100 mL/kg/d. | Collection of milk every 15 d from birth until infants reached corrected GA 40 weeks, expressed by hand. | AAS | 38 |
| De Oliveira Trinta 2020 (200) | Cross-sectional study | Brazil | Women 21-43 years old, no chronic disease, no HIV, no medication incompatible with breastfeeding, no complications with breastfeeding (such as mastitis or fissures). | Preterm (n=7) and term (n=11) infants, mean birthweights of 1.7 and 3.5 kg respectively. | Collection of colostrum (<7 d pp), transitional (7-14 d pp) and mature (>14 d pp) milk. Stored at -18°C until analysis. | ICP-MS | 18 |
| Dempster 1981 (201) | Cross-sectional study | South Africa | Women, well-nourished. | Healthy full-term infants. | Collection of transitional milk (7 days pp), extracted manually. Stored at -80°C until analysis. | AAS | 48 |
| Deng 2009 (44) | Cross-sectional study | China | Healthy women 18-36 years, non-smoking, no chronic medication, parity of 1 or 2. |  | Collection of mature milk, from 3 weeks to 2 months pp. | ICP-MS | 60 |
| Dewey 1983 (46) | Cohort study | USA | Women 20-36 years, high education, some taking supplements (n=16), parity 1-2. | Infants, 1-6 months, at least partially breastfed. | Collection of milk monthly (1, 2, 3, 4, 5, 6 months pp), from one breast at 2nd feeding of the morning, extracted by hand. Frozen until analysis. | FAAS | 20 |
| Dewey 1984 (47) | Cohort study | USA | Women 21-37 years, vegetarian and non-vegetarian. |  | Collection of milk at 4-6 months, 7-11 months and 12-20 months pp, extracted by hand or manual pump, at 2nd feeding of morning, monthly or bimonthly. Stored at -20°C until analysis. | FAAS | 46 |
| Dhonukshe-Rutten 2005 (202) | Cross-sectional study | Guatemala | Women 15-37 years, mean parity 3, from low income areas, some with intestinal parasites. | Healthy infants, 1-6 months. | Collection of mature milk, at least 1h after last feed, full breast while infant suckled on other breast, between 8:30AM-11:30AM, extracted with manual pump. Stored in cooler and -20-40°C until analysis. | ICP-AES | 47 |
| Dijkhuizen 2001 (203) | Cross-sectional study | Indonesia | Healthy women 15-41 years, BMI 19.7-23.0, with no chronic or severe illness, no clinical malnutrition. | Healthy infants 2.4-10.5 months, singleton, no congenital anomalies, no conditions. | Collection of mature milk, complete right breast, 45-60 min after last feeding from breast, extracted by manual pump. | AAS | 155 |
| Djurovic 2017 (204) | Cohort study | Serbia | Healthy women 16-36 years, non-smoking. | Infants, GA 38-42 weeks, birth weight 2.4-4.1 kg, exclusively breastfed, some with neonatal jaundice (n=20) and IUGR (n=3). | Collection of colostrum (1 d pp) and mature milk (28 d and 6 months pp), with manual pump or passive breast milk sampler, fore- and hindmilk. Stored at -20°C until analysis. | ICP-OES | 60 |
| Domellof 2004 (205) | Cross-sectional study | Honduras, Sweden | Women >=16 years. | Full-term infants, 9 months old, birth weight >2.5 kg, no chronic illness, exclusively breastfed at 4 months. | Collection of mature milk (9 months pp), >=1h after previous feeding, extracted by hand or manual pump. Stored at -20°C until analysis. | AAS | 191 |
| Donangelo 1989 (206) | Cross-sectional study | Brazil | Women, mean age 26 ±6 years, low SES, receiving supplements (iron, vitamin B12, and some also folate), 66% vaginal delivery, 53% primiparous, 66% non-smoking. | Infants, exclusively breastfed and partially weaned, mean birth weight 3.3 ±0.5 kg. | Collection of milk from different stages of lactation from birth to 9 months pp, 9-10AM, extracted manually, before feed. Stored at -20°C until analysis. | AAS | 83 |
| **Doneray 2017** (207) | Cohort study | Turkey | Women, uncomplicated pregnancy, no delivery in the last 2 years, no pre-existing maternal disease, no pre-eclampsia, BMI <=35, no hypertension, non-smoking, no hormone or drug therapy, no lactation failure. | Term infants, singleton, exclusively breastfed, not LGA or SGA, no fetal malformation. | Collection of transitional (8-12 d pp) and mature (25-30 d pp) milk, fore- and hindmilk. Stored at -20°C until analysis. | AAS | 37 |
| Dorea 1985 (208) | Cohort study | Brazil | Healthy women, from low (n=8) and high (n=1) socioeconomic group. |  | Collection of mature milk 15, 30, 45, 60, 75, 90 d pp, fore- and hindmilk. | AAS | 9 |
| Dorea 1993 (209) | Cohort study | Brazil | Women, mean age 20-31±3-6 years, primiparous and multiparous, not (n=22) or taking contraceptives (combined pills n=15, minipills n=29, injectable progesterone n=13 or intrauterine device n=5). |  | Collection of milk before (15-380 d pp) and after 20 days (31-394 d pp) initiating contraception, 7:00-11:30 AM. | AAS | 81 |
| Dorea 2002 (210) | Cross-sectional study | Brazil | Women, at the university of Brasilia. | Infants 1-6 months old. | Collection of mature milk 1-6 months pp, expressed manual, in plastic bottles. Stored at -20°C until analysis. | ICP-AES | 58 |
| Dumrongwongsiri 2015 (211) | Cross-sectional study | Thailand | Women, mean age 30-31±5-6 years. | Healthy full-term infants, 4-6 months, exclusive, partial breastfed and formula fed, no illness within 4 weeks before study, not taking supplements, birth weight 3.1-3.2 kg. | Collection of mature milk, beginning of feed, from one breast, 2PM-4PM, extracted with electric pump. Stored at -80°C until analysis. | ICP-MS | 34 |
| **Dumrongwongsiri 2021** (49) | Cross-sectional study | Thailand | Healthy women, no medication. | Term infants, 4-6 months old, exclusively and non-exclusively breastfed. | Collection of milk samples at 4-6 months pp, 2-5 PM, after cleaning breast, first 10-15 ML from one breast. Stored at -80°C until analysis. Calcium and zinc were measured with ICP-MS. | ICP-MS | 34 |
| Dumrongwongsiri 2022 (212) | Cohort study | Thailand | Healthy women. | Infants, singleton, breastfed. | Collection of mature milk at 2 and 4 months pp, with electric pump. Stored at -80°C until analysis. Zinc was measured with ICP-MS. | ICP-MS | 64 |
| Duncan 1988 (213) | Cross-sectional study | South Africa | Healthy women 19-27 years, no dietary supplements, white women from upper-middle SES and Indian and colored women from low SES. |  | Collection of colostrum-transitional milk 2-7 d pp, by hand expression, from left breast after feeding. Stored frozen until analysis. | AAS | 107 |
| Durovic 2017 (214) | Cross-sectional study | Serbia | Healthy women, mean age 31 ±6 years. | Term infants, 1 day old. | Collection of colostrum milk on day 1 pp, extracted with manual pump or passive breast milk sampler, from beginning and end of feeding session. Stored at -20°C until analysis. | ICP-OES | 28 |
| Edem 2016 (215) | Cross-sectional study | Nigeria | Healthy women 19-40 years, mean BMI 23 ±.4, no malignant conditions, 17% with zinc deficiency. | Term infants, 42-180 days old. | Collection of mature milk (42-180 d pp), by manual expression. Stored at -20°C until analysis. | AAS | 92 |
| Ehsani 2009 (216) | Controlled trial | Iran | Healthy women, mean age 23±4-5 years, 66-68% medium SES, 26-27% good SES, 6% very good SES, assigned to iron supplementation (50 mg/d) or placebo. |  | Collection of milk samples at 14 and 40 d pp. | AAS | 156 |
| Ejezie 2011 (217) | Cohort study | Nigeria | Women 19-42 years old, no zinc supplement, non-smoking, HIV negative, no obstetric hemorrhage, fever, sickle cell disease, diabetes, malignancy, chronic alcoholism, tuberculosis, hypertension or diagnose inflammatory disorder. | Infants, singleton. | Collection of colostrum, transitional and mature milk at 0-15 d pp, by manual expression, fore- and hindmilk, 9-11 AM. Stored at -20°C. | AAS | 40 |
| El Din 2004 (218) | Cross-sectional study | Egypt | Healthy women 17-40 years, parity 1-9, from low or intermediate SES, without disease, malnutrition or medication. | Infants, from 2 weeks to 1 year old. | Collection of mature milk, extracted with hand or pump. Analyzed immediately. | AAS | 61 |
| El-Farrash 2012 (51) | Case-control study | Egypt | Women, mean age 25-27±3-5 years, mean BMI 27±3, mean parity 2.6±1, healthy or anemic (hemoglobin <11 g/dL), no iron supplementation during pregnancy, no other causes of anemia, no pregnancy-induced hypertension, no diabetes, no prolonged rupture of membranes, no fever or foul smelling liquor, no antepartum hemorrhage, no mastitis, no other systemic illness. | Term infants, mean birth weight 2.5-3.3±0.3-4 kg, exclusively breastfed, singleton, no hemolytic disease, no neonatal sepsis. | Collection of transitional/mature milk 15±3 d pp, by manual expression, after discarding 4-5 mL initial milk. Stored at -20°C until analysis. | AAS | 80 |
| Elmastas 2005 (219) | Cross-sectional study | Turkey | Healthy women. |  | Collection of mature milk at 2 months pp, expressed with manual pump, two complete breasts, between 10AM-3PM. Stored at -20°C until analysis. | FAAS | 32 |
| Ergul 2010 (220) | Cohort study | Turkey |  | Infants, preterm (n=47) and term (n=41). | Collection of colostrum (0-3 d pp), transitional (7-14 d pp), and mature milk (30-40 d pp), expressed by hand from both breasts, at any time of the day, at least 30 min before breastfeeding. Stored at -20°C until analysis. | ICP-AES | 88 |
| **Ezechukwu 2004** (221) | Cross-sectional study | Nigeria | Healthy women 18-35 years, parity 1-6, lactating well. | Full-term healthy infants 1-6 months, well-nourished. | Collection of mature milk 1-6 months pp, by manual expression, from both breasts. Stored at -20°C until analysis. | AAS | 120 |
| Feeley 1983b (222) | Cohort study | USA | Healthy women 16-38 years, from middle SES, parity 1-2, 94% took supplements (96% iron, 38% zinc, 38% copper). | Healthy full-term infants, birth weight 2.5-4.8 kg. | Collection of colostrum (4-7d pp), transitional milk (10-14d) and mature milk (30-45d pp), from beginning, middle and end of feeding, from morning and evening feeds, extracted by hand or manual pump. Stored at -20°C until analysis. | ICP-AES | 102 |
| Fernandez-Menendez 2016 (223) | Cohort study | Brazil, Spain | Women, from Spain (n=31) and Brazil (n=1), no underlying chronic disease, no zinc supplementation during pregnancy. | Infants, preterm and full-term. | Collection of colostrum, transitional (7, 14 d pp) and mature (28 d pp) milk, by manual expression, in the morning, >=1h since previous feeding. Stored at -20°C until analysis. | ICP-MS | 32 |
| Fouche 2016 (224) | Cohort study | South Africa | Women, mean age 28 years, with HIV receiving ART (n=38) or without HIV with premature infants. | Preterm infants, gestational age 24-37 weeks. | Collection of transitional milk 9-10 d pp, by manual expression. Stored at -20°C until analysis. | ICP-OES | 74 |
| Fransson 1982 (54) | Cross-sectional study | Sweden | Healthy women. |  | Collection of milk, 2nd feeding of the day, 9-11 AM, one complete breast, with hand pump. Stored at -20°C until analysis. | AAS | 23 |
| Fransson 1983 (55) | Cross-sectional study | USA | Healthy women. |  | Collection of mature milk (0.5-12 months pp), one complete breast, 2nd feeding of the day, extracted with manual pump. | AAS | 30 |
| Fransson 1984 (56) | Cross-sectional study | Ethiopia, Sweden | Healthy women, normal blood pressure, no glucose or protein in urine, normal hemoglobin values, normal deliveries, from Ethiopia (n=18, mean age 19-23) with iron-rich diet from both non- and privileged groups and women from Sweden (n=23, mean age 28) with normal pregnancy and no medication but with supplements. | Infants, from Ethiopia (mean GA 38-41 weeks, mean birth weight 2.5-3.2 kg) and from Sweden (GA 37-42 weeks, mean birth weight 3.6 kg). | Collection of colostrum (4-5 d pp), from Ethiopia from both complete breasts at first feeding of day, from Sweden from one complete breast 2nd feeding of day. Stored at -20°C until analysis. | AAS | 41 |
| Friel 1999 (57) | Cohort study | Canada | Women 20-35 years, with no known pathological condition, not vegetarian, mainly European origin, well-nourished. | Full-term and preterm infants. | Extraction with electric pump (15 mL, at 2-3 days pp and week 2, 3, 4, 5, 6, 7, 8 and 12 pp), during a regular feed, 15 mL from one whole breast, between 10 AM and 2 PM. Stored at -20°C until analysis. | ICP-MS | 43 |
| Frkovic 1996 (225) | Cohort study | Croatia | Women 17-45 years, parity 1-7. | Infants, birth weight 2.2-4.4 kg. | Collection of colostrum and transitional milk on 2 and 12 d pp. | FAAS | 29 |
| **Fung 1997** (226) | Cohort study | USA | Women 22-40 years, BMI 19-26, non-smoking, non-diabetic, non-vegetarian, no drug or alcohol abuse, no previous obstetric or gynecologic complication, White (n=11) and Hispanic (n=2), given daily zinc-free multivitamin-mineral supplement, from middle-upper SES. | Healthy full-term infants, mean GA 39.5 weeks, mean birth weight 3.65 kg. Extracted values for exclusively breastfed. | Collection of mature milk samples 3x over 72h at 43-75 d pp, on breast opposite to offered infant, complete breast. Stored at -20°C until analysis. | AAS | 9 |
| Garg 1988 (58) | Cross-sectional study | India | Healthy women 18-30 years, parity 1-4, well-nourished (n=20) and under-nourished (n=15), vaginal delivery. | Full-term infants. | Collection of colostrum 0-3 days pp, in the morning before feeding. Calcium and zinc were measured with AAS. | AAS | 35 |
| **Garza 1983** (59) | Cohort study | USA | Healthy women, 26-35 years, parity 1-2, no medication, no oral contraceptive, limited coffee, tea and alcohol intake to <2/d, nonsmoking. | Healthy normal term infants, 5-7 months, AGA, exclusively breastfed at baseline and gradually weaning over 3 months. | Collection of mature milk samples at baseline, before weaning, and then during weaning every 2 weeks for 12 weeks, 8:00AM-12:00AM, from one complete breast 3-4h after previous nursing, extracted with pump. Stored at 4°C and then -20°C until analysis. | AAS | 6 |
| Gates 2021 (60) | Cohort study | USA | Women, 18-37 y, not vegan or vegetarian, not restricting caloric intake, HIV negative. | Preterm (≤33 GA) infants. | Collection of milk at 7, 14, 21 and 28 d pp, with electric breastpump, 24h collection, emptying both breasts every 3h. Stored at -29°C until analysis. Vitamin D was measured with UV-HPLC. | ICP-OES | 36 |
| **Gibson 2020** (61)^[[12]](#footnote-12)^ | Cohort study | Indonesia | Women, mean age 28±6 years, BMI at 5 months pp mean 24±4, 33% primiparous, from urban and rural site, no chronic disease, no acute malnutrition. | Healthy full-term infants, 2 and 5 months old, birth weight >=2.5 kg, exclusively breastfed, no chronic disease or malnutrition. | Collection of mature milk (2 and 5 months pp), extracted with pump, from one complete breast, in the morning. Frozen at -80°C until analysis. | ICP-MS | 212 |
| Goes 2002 (64) | Cross-sectional study | Brazil | Healthy adult women in general. | Term infants in general. | Human milk bank. Collection of mature milk, extracted by hand or manual/electric pump or drip milk. Stored at -20°C until analysis. | AAS | 60 |
| Gross 1998 (227) | Cross-sectional study | Indonesia | Women 18-40 years, living in middle-income urban area of Jakarta, mean BMI 16.2-33.9, no undernutrition | Infants 0.1-5.2 months old (mean age 2.4 months), birth weight 1.7-4.3 kg, no undernutrition. | Collection of mature milk, from one breast during 5 consecutive days, 9-11AM, with manual pump. Stored at -20°C until analysis. | ICP-AES | 91 |
| Gulson 2001 (68) | Cross-sectional study | Australia |  |  |  | ICP-MS | 17 |
| Gupta 1984 (69) | Cohort study | India | Women, no anemia, no undernutrition, no vitamin deficiency. | Infants, term (n=50, mean GA 39 weeks) and preterm (n=14, mean GA 35 weeks). | Collection of colostrum/transitional/mature milk, (2-5, 6-10, 11-30 d pp) by manual expression, foremilk, in morning. Stored at -20°C until analysis. | FAAS | 64 |
| Hampel 2018 (228) | Randomized controlled trial | Malawi | Women, HIV infected, randomized to control group (no LNS, no ARV). | Infants, singleton, HIV negative, exclusively breastfed up to 24 weeks old. | Collection of milk at 2 or 6 and 24 weeks pp, manually expressed. Stored -80°C until analysis. | ICP-AES | 177 |
| Hannan 2005 (229) | Cohort study | Libya | Healthy women 20-40 years old. |  | Collection of colostrum, transitional and mature milk 0-20 d pp, expressed manually. Freeze-dried and stored frozen until analysis. | INAA | 25 |
| Hannan 2009 (230) | Cross-sectional study | USA | Healthy women, 20-38 years (mean 28 years), BMI 18-42 (mean 29), in WIC program (low income) in Texas. |  | Collection of mature milk (30-45d pp and 75-90d pp), extracted by hand or pump. Stored at -20°C until analysis. | FAAS | 30 |
| Harzer 1986 (71) | Cohort study | Germany | Healthy women. | Term infants. | Collection of milk 1, 3, 5, 15, 22, 29, 36 d pp, from both breasts complete during 24h, with electric breast pump. Stored at -20°C until analysis. | AAS | 10 |
| Hibberd 1982 (72) | Cohort study | Germany | Healthy women. | Term infants. | Collection of transitional and mature milk during the first 5 weeks (3, 5, 7, 11, 18, 25, 32, 39 d pp), both complete breasts, extracted with electric pump. Stored at -20°C until analysis. | AAS | 10 |
| Higashi 1982 (231) | Cohort study | Japan | Women 21-37 years, no complication during pregnancy or delivery, primiparous (n=20) and multiparous (n=45), good nutrition condition based on clinical observation. | Healthy full-term infants, birth weight >2.5 kg. | Collection of colostrum, transitional (1 week pp) and mature (1, 3, 5 months pp) milk, by manual expression, in morning, before feeding. | AAS | 65 |
| Honda 2003 (73) | Cross-sectional study | Japan | Women 19-38 years old, Japanese, living in non-industrial area close to the hospital, vaginal and cesarean section. |  | Collection of transitional milk 5-8 d pp. Stored at -20°C until analysis. | FAAS | 68 |
| Hou 2008 (74) | Cross-sectional study | China | Healthy women 18-36 years, non-smoking, no chronic medication, parity of 1 or 2. | Term infants, exclusively breastfed. |  | AAS | 240 |
| Huang 2014 (76) | Cross-sectional study | China | Healthy women 18-40 years of age, non-smoking, no alcohol consumption. | Infants <1 month old, singleton. | Collection of milk, by trained staff, 8am-11am, with electronic pump. Stored at -80°C within 4 hours after extraction until analysis. | ICP-MS | 269 |
| Hunt 2004 (232) | Cohort study | Canada | Women, mean 31±4-5 years, European origin. | Preterm and full-term infants. | Collection of transitional and mature milk weekly from birth to 12 weeks, expressed by pump or hand, before feeding, 10AM-2PM, from right breast. Stored at -20°C until analysis. | ICP-OES | 20 |
| **Hunt 2005** (77) | Cohort study | USA | Healthy women 18-36 years, non-smoking, no medication, parity 1-2, mainly Caucasian. | Full-term infants, exclusively breastfed, AGA, birth weight 2.56-4.57. | Collection of mature milk at 1, 2, 3, 4 months pp, during 24h content of one breast not offered to infant, extracted with pump. Stored refrigerated up to 24h. | ICAPS | 36 |
| Islam 2014 (233) | Cohort study | Bangladesh | Women, BMI at 4-24 weeks pp mean 19-22±2-3, from low-income community, no pregnancy-induced hypertension, no known HIV or hepatitis B infection, non-smoking, no alcohol. | Term infants, singleton, AGA (n=20) and SGA (n=26), exclusively breastfed, no congenital abnormality, no chronic infection, no acute illness. | Collection of milk on day 1 and 5 of weeks 4, 12, 24 pp, after 2-3 min feeding, expressed manually. | AAS | 46 |
| Itabashi 1999 (78) | Cohort study | Japan |  | Preterm infants (GA 26-33 weeks). | Collection of milk at 1, 2, 3, 4, 5-6, 7-8, and 9-12 weeks pp, by manual expression, both complete breasts, in morning just before lunch. Stored at -40°C until analysis. | ICP-AES | 15 |
| Itriago 1997 (79) | Cohort study | Venezuela | Women >18 years old, living in city of Caracas, 77% from low-middle SES. | Term infants, singleton. | Collection of colostrum, transitional and mature milk at 3, 7 and 21 d pp, 7-10AM, expressed manually. Stored at -15°C until analysis. | ICP-AES | 72 |
| Iwai 2022 (80) | Cross-sectional study | Japan | Healthy women 29-40 y. | Healthy children 3-5 months old. | Collection of milk 3-5 months pp, with manual expresion fore- and hindmilk, in the morning, afternoon and evening. Stored at -80°C until analysis. | ICP-MS/MS | 11 |
| Jagodic 2020 (234) | Cross-sectional study | Slovenia | Women 19-39 years old, primiparous, living in coastal area (frequent consumption of seafood) and inland area (less frequent consumption of seafood). | Infants, singleton, mean birth weight 3.3 kg. | Collection of milk 5-11 weeks pp. Stored at -20°C until analysis. | ICP-MS | 74 |
| Jariwala 2014 (235) | Cross-sectional study | India | Healthy women <40 years old, vaginal delivery, no antenatal illness (such as toxemia, diabetes, hypothyroidism), taking only iron, calcium and folic acid supplement. | Healthy term infants, singleton, no visible congenital abnormality. | Collection of colostrum on 3 d pp. Stored at -20°C until analysis. | FAAS | 42 |
| Kantola 2001 (236) | Cross-sectional study | Finland | Healthy women, mean age 28-29±4-5 years, primiparous and multiparous, non- and smoking, in urban and rural areas. |  | Collection of mature milk 4 weeks pp, in morning and afternoon, after 5 min feeding. Stored -20°C until analysis. | FAAS | 257 |
| Karra 1986 (84) | Cohort study | USA | Women 21-38 years old, no known disease, with income to enable nutritionally adequate diet, half were consuming vitamin supplements at the beginning of the study. | Infants, not exclusively breasted. | Collection of mature milk monthly 7-25 months pp, foremilk at a single feeding, 7-10AM, expressed manually or with pump. Stored frozen and then at -30°C until analysis. | AAS | 55 |
| Karra 1988 (85) | Cohort study | Egypt, USA | Women, from United States (n=49) 21-38 years old from middle-income SES with some receiving supplements without (n=25) and with (n=24) 25 mg/d zinc, and from Egypt (n=68) 17-34 years, from marginally malnourished, low-income, illiterate, rural population, no supplements. |  | Collection of mature milk at 1, 2, 3, 4, 5, 6 months pp, expressed manually or with pump, after milk letdown, 24h sample or 10 AM-2 PM sample. Stored at -30°C until analysis. | AAS | 117 |
| Karra 1989 (237) | Controlled trial | Egypt, USA | Women, from urban United States middle-income group (n=33) and rural Egypt low SES (n=30), supplemented with 0 or 50 mg/d zinc for 34 days from 7-9 months pp. |  | Collection of mature milk at 1-2 (baseline, 7-9 months pp), 13, 18, 25, 33-34 d after start of intervention, expressed manually or with pump, after letdown, 24h or 6AM-12PM or 10-12AM collections. Stored at -30°C until analysis. | AAS | 63 |
| Keizer 1995 (238) | Randomized controlled trial | Canada | Adolescents 14-19 years, no anemic, 92% Caucasian, randomized to 0 or 300 ug/d folic acid supplement for 4 weeks starting within 1 week pp. | Term infant, singleton. | Collection of mature milk at 4, 8, 12 weeks pp, from one complete breast, 1-2:30PM. | FAAS | 71 |
| Khaghani 2010 (239) | Cross-sectional study | Iran | Women, non-smoking, normal pre-pregnancy weight and pregnancy weight gain. | Infants, 2 months old. | Collection of mature milk, extracted manually. Stored at -20°C until analysis. | FAAS | 30 |
| Khatir Sam 1998 (87) | Cross-sectional study | Sudan | Women, volunteer nurses. |  | Collection of milk, morning samples on 2 consecutive days, sometimes in morning and at noon, expressed with hand pump. | XRF | 20 |
| Kim 2012 (240) | Cohort study | South Korea | Women 24-40 years, mean BMI 22±4 (range: 16-34), non-vegetarian, not taking multivitamins or medications to increase milk production. | Preterm infants, GA 25-37 weeks, birth weight 0.7-2.7 kg. | Collection of transitional and mature milk (end of week 1, 2, 3, 4, 6, 8, 12 pp), expressed manually or with electric pump, early morning. Stored at -70°C until analysis. | AAS | 67 |
| **Kim 2017** (88) | Cross-sectional study | South Korea | Women 21-45 years, BMI 15-33 (mean 22), 51% took dietary supplements. | Infants, 30-360 days of age, term born 37-42 weeks, normal birth weight 2.5-4.5 kg, exclusively breastfed. | Collection of mature milk from full breast, extracted with pump. Stored at -20°C until analysis. | ICP-OES | 334 |
| Kippler 2009 (89) | Randomized controlled trial | Bangladesh | Women participating in micronutrient supplementation trial. |  | Collection of mature milk at 2 months pp, expressed by hand. Stored at -70°C until analysis. | ICP-MS | 123 |
| Kippler 2012 (90) | Cross-sectional study | Bangladesh | Women, median age 7 (10-90th percentile: 20-36 years old), mean BMI 19, primiparous (n=57) and multiparous (n=43), from rural area, received food supplement and micronutrient supplement (with iron and folic acid or 15 different micronutrients, not containing zinc nor calcium). | Infants, singleton. | Collection of mature milk at 2 months pp, expressed by hand, from one or if insufficient two breasts. Stored at -70°C until analysis. | ICP-MS | 123 |
| Kirksey 1979 (91) | Cross-sectional study | USA | Women 18-31 years, Caucasian, middle SES, primiparous and multiparous, no difficulties with delivery, normal eating habits, some used oral contraception (n=14), with iron supplements (all except one) and other nutrients (n=21). |  | Collection of colostrum, transitional and mature milk 3d, 14 d, 1-3 months, 5-7 months, 1 year pp, before taking supplement, expressed manually. Stored at -20°C until analysis. | AAS | 52 |
| Kirsten 1985 (241) | Cohort study | South Africa | Women 18-43 years old, primiparous and multiparous, from middle-upper SES, uncomplicated pregnancy and delivery, no anemia, no history of bacterial or viral infection, no antibiotic therapy, no hepatitis, no blood transfusions, no antepartum hemorrhage, no serious illness during pregnancy. | Healthy infants, breastfed, mean birth weight 3.1 kg. | Collection of colostrum, transitional and mature milk on 3d, 7d, 6, 12, 24, 36, 52 weeks pp, fore- and hindmilk from morning feed 9-12 AM. Stored at -80°C until analysis. | AAS | 55 |
| Klein 2017 (92) | Cross-sectional study | Argentina, Namibia, Poland, USA | Women, no indication of mastitis, non-smoking. | Infants 2 weekss-2 years old, singleton. | Collection of mature milk (2 weeks-2 years pp), mid-feed, 8-11:30AM, 2h since last feed, expressed by hand. Stored at -20 or -80°C until analysis. | ICP-MS | 70 |
| Kosanovic 2008 (242) | Cross-sectional study | United Arab Emirates | Milk donors. |  | Collection of mature milk from milk donors, extracted of both breast completely. Stored at -30°C until analysis. | ICP-MS | 120 |
| **Krebs 1985a** (243) | Cohort study | USA | Healthy women, mean age 28±4 years, middle-income, unremarkable obstetric histories. |  | Collection of mature milk, mid-feed or from beginning, middle and end of feed, from 1 month pp, monthly up to 12 months pp. Stored at -20°C until analysis. | FAAS | 48 |
| Krebs 1985b (244) | Cohort study | USA | Healthy women mean age 28-30±3 years, unremarkable obstetric history, mainly Caucasian, from middle-income families, mean parity 1.5, received a daily zinc supplement or not. | Healthy term infants. | Collection of milk, extracted manually. Stored at -20°C until analysis. | FAAS | 39 |
| **Krebs 1995** (245)^[[13]](#footnote-13)^ | Randomized controlled trial | USA | Healthy women, mean age 30±4 years, parity 1-5, all white except one, from middle-income families, well educated, uncomplicated pregnancy, labor and delivery, randomized to zinc supplements (15 mg zinc/d) or placebo. | Healthy term infants, AGA, mean birth weight 3.4±.4 kg. | Collection of mature milk monthly from 0.5-9 months pp, expressed by hand, midfeed. Stored at -20°C until analysis. | FAAS | 71 |
| Lamounier 1989 (246) | Cohort study | Brazil | Women 17-39 years, low SES, normal delivery, white (n=12) and non-white (n=11), primiparous (n=6) and multiparous (n=17). | Infants, birth weight 2.5-4.0 kg. | Collection of colostrum (3-7 d pp) and mature milk (at 15d intervals, until 6 months pp), 9 AM-5 PM, by manual expression, both breasts. Stored at -15°C until analysis. | AAS | 23 |
| Lastre-Amell 2020 (247) | Cross-sectional study | Venezuela | Healthy women 18-35 years, no conditions, non-smoking. |  | Collection of milk, expressed manually. Stored on ice during transport. | FAAS | 91 |
| Lauber 1979 (248) | Cohort study | Ivory Coast | Women, living in rural area, primiparous (n=8) and multiparous (n=24). | Infants 1-16 months old. | Collection of mature milk (1-16 months pp), every 4 weeks, in the morning, from one complete breast, breast not suckled on during whole night, expressed with electrical pump and occasionally manually. Stored at -20°C until analysis. | AAS | 33 |
| Leotsinidis 2005 (249) | Cohort study | Greece | Women 16-39 years, 66% non-smoking, 72% vaginal delivery, 53% primiparous, with and without supplement (9% vitamin A&E, 94% calcium, 98% iron, 16% magnesium). | Healthy full-term infants, birth weight 1.8-4.5 kg. | Collection of colostrum and transitional milk at 3 and 14 d pp, in the morning, 2h after last feeding, expressed by hand. Stored at -20°C until analysis. | FAAS | 180 |
| Levi 2018 (98) | Cohort study | Argentina | Women, exposed to varying concentrations of drinking water pollutants. | Infants, exclusively breastfed. | Collection of mature milk (0-3 and 3-6 months pp), expressed by gentile compression. Stored at -20 and then -80°C until analysis. | ICP-MS | 237 |
| Li 1990 (99) | Cross-sectional study | Japan | Healthy women, Japanese, mean age 29±5 years, mean BMI 22±2. | Full-term infants, mean birth weight 3.1±0.4 kg. | Collection of transitional milk (6-9 d pp), in the morning. Stored at -20°C until analysis. | ICP-AES | 27 |
| Li 2013 (250) | Trial | China | Healthy women 22-35 years, non-smoking, no chronic medication, parity of 1 or 2, received dietary counselling for one week. | Healthy full-term infants, 1 month old at baseline, exclusively breastfed. | Collection of milk samples from both breasts, before and after intervention (at 1 and 3 months). Stored at -20°C until analysis. | AAS | 50 |
| Li 2016 (101) | Cross-sectional study | Guatemala | Women, mean age 24±6-7 years, mean parity 3±2, with no indication of mastitis. | Infants 5-180 days old, exclusively (56-71%) or predominantly breastfed, 7-16% underweight, 30-45% stunted. | Collection of transitional (5-17 d pp) and mature (18-180 d pp) milk, in the morning, from breast not recently suckled on, by full manual expression. Stored at -30 and then -80°C until analysis. | ICP-MS | 228 |
| Li 2018 (102)^[[14]](#footnote-14)^ | Cross-sectional study | Guatemala | Women, mean age 25, mean BMI 24, primi- (30%) and multiparous (70%), Guatemalan Mam-Mayan. |  | Collection of transitional and mature milk 5-17 d, 18-46 d, 4-6 months pp. Stored at -30°C and then at -80°C. | ICP-MS | 108 |
| **Lin 1998** (104) | Cohort study | China | Healthy women >=20 years, primiparous (n=84) and multiparous (n=127), uncomplicated pregnancy. | Healthy term infants, singleton, growing well. | Collection of colostrum (2-5 d pp), transitional (6-10 d pp) and mature milk (11-365 d pp), expressed with manual pump, one complete breast, second feeding of the day, 9-11AM. Stored at -40°C until analysis. | ICP-AES | 211 |
| Lipsman 1985 (105) | Cohort study | USA | Adolescents 14-20 years, from low-middle income families, White (n=12), Hispanic (n=8), Black (n=3), American Indian (n=2). | Infants, mean birth weight 3.3 kg. | Collection of mature milk monthly 1-10 months pp, from one breast, expressed manually or with manual pump, 9AM-6PM. Stored at -20°C until analysis. | FAAS | 25 |
| Liu 2008 (106) | Cross-sectional study | China | Healthy women 18-36 years, non-smoking, no chronic medication, parity of 1 or 2. |  | Collection of mature milk. | GB/AAS | 99 |
| Liu 2014 (107) | Cohort study | China | Healthy women 20-37 years, no chronic diseases, parity of 1 or 2. |  | Collection of colostrum (3 day postpartum) and mature milk (90 day postpartum). Stored at -40°C until analysis. | AAS | 352 |
| Lonnerdal 1982 (251) | Cross-sectional study | USA | Milk donors. |  | Milk from Mothers Milk Bank. | FAAS | 5 |
| **Lonnerdal 1996** | Cohort study | Peru | Women 18-44 years, with and without acute febrile infection during labor or early postpartum, BMI >20. | Healthy term infants, birth weight >2.5 kg, exclusively breastfed. | Collection of colostrum and transitional milk at 48h and 14 d pp. Stored at -20°C until analysis. | FAAS | 57 |
| Luo 2010 (108,252) | Cross-sectional study | China | Healthy women 22.5-34.7 years. |  | Collection of milk, from right breast, expressed by hand or with manual or electric pump. Stored at -20°C until analysis. | FAAS | 758 |
| Maeda 1990 (253) | Cohort study | Japan | Women 22-40 years, primiparous (n=17) and multiparous (n=20), normal delivery, 15-18% with zinc deficiency (<=0.5 ug/mL) at 1 week pp, no zinc deficiency at 1 month pp. |  | Collection of colostrum/transitional milk (2-8 d pp) and mature milk (20-30 d pp), 10-11 AM. | FAAS | 37 |
| **Mahdavi 2010** (254) | Cross-sectional study | Iran | Women mean age 27±6, mean BMI 27±4, from rural and urban areas, from low, intermediate and high SES, not taking trace element supplements. | Healthy full-term infants, exclusively breastfed, 90-120 d old, normal birth weight, no chronic disease. | Collection of mature milk, in the morning before nursing. Stored at 4°C and then -20°C. | AAS | 91 |
| **Mahdavi 2015** (109) | Randomized controlled trial | Iran | Women, mean age 26-28±5 years, mean BMI 28-29±11, no chronic illness, no gastrointestinal disorder, no supplements, no antibiotics, no corticosteroid, living in urban areas, were randomized to synbiotic supplement (n=27) or placebo (n=27). Data from baseline and placebo group were used. | Full-term infants 3-4 months old, normal birth weight 2.5-4.0 kg, exclusively breastfed, no chronic illness, no gastrointestinal disorder, no supplements, no antibiotics, no corticosteroid. | Collection of mature milk 90-120 d pp, by self-expression, in the morning, before first feeding, after discarding the first 5 ccml. Stored at 4 and then -80°C until analysis. | FAAS | 57 |
| Mandia 2021 (110) | Cohort study | Spain | Women 24-44 y, without chronic disease, not taking nutrient supplements. | Term and preterm infants. | Collection of colostrum (3-4 d pp, only term infants), transitional (7-10 d pp, only term infants) and mature milk (from both preterm and term infants), by manual expression. Stored -20°C until analysis. | ICP-MS | 170 |
| Mandic 1997 (255) | Cross-sectional study | Croatia | Women, mean age 26 years, primiparous (n=14) and multiparous (n=28), non- (n=30) and smoking (n=12), refugee (n=20) and control (n=22). |  | Collection of colostrum/transitional and mature milk 1->60 d pp. | FAAS | 42 |
| Martinez 2019 (256) | Cross-sectional study | Spain | Healthy women 25-43 years old, Spanish, mean BMI 24.5. | Healthy infants 1-18 months old. | Collection of mature milk 1-18 months pp. Stored at -20°C until analysis. | ICP-MS | 53 |
| Maru 2013 (111) | Cross-sectional study | Ethiopia | Healthy women 18-35 years old, no supplementation except from iron/folic acid supplement, in rural and urban area. | Healthy full-term infants. | Collection of colostrum milk (within 4 d pp), by manual expression. Stored at-20°C until analysis. | FAAS | 45 |
| Mastroeni 2006 (112) | Cohort study | Brazil | Women, mean age 23±5 years, mean parity 2.7±1.5, vaginal (n=27) and caesearan (n=4) delivery. | Term infants, mean birth weight 3.1±0.5 kg. | Collection of colostrum (2 d pp) and mature milk (2 months pp), by manual expression. Stored at -20°C until analysis. | ICAP-AES | 43 |
| **Matos 2009** (257) | Cohort study | Portugal | Women 21-39 years, no pathology preventing breast-feeding (tumor, HIV, hepatitis B or C carrier), no pharmacological treatment preventing breast-feeding. | Healthy term infants, with adequate weight to gestation age, exclusively breastfed until 4 months of age. | Collection of transitional and mature milk (at 1, 4, 8, 12, 16 weeks pp), over a 24h period, before and after each feed, from each breast, extracted with hand or pump. Stored in freezer for max 24h and then -80°C until analysis. | ICP-MS | 31 |
| **Matsuda 1984** (258) | Cohort study | Japan | Women, with good nutritional status. | Healthy full-term infants, birth weight >2.5 kg, exclusively breastfed. | Collection of transitional and mature milk at 1 week, 1, 3, and 5 months pp, in the morning, by manual expression. | AAS | 18 |
| **Mbofung 1984** (259) | Cohort study | Nigeria | Women 17-40 y, from low SES, healthy. | Term normal healthy infants 0-32 weeks old, exclusively breastfed up to 6 months. | Collection of milk (0-36 weeks pp), 9-12 AM, by manual expression, before feeding infant. | AAS | 240 |
| Mello-Neto 2009 (260) | Cross-sectional study | Brazil | Milk donors 16-41 years, BMI 17-41, primiparous (57%) and multiparous (43%), non- (85%) and smoking (15%), not (14%) and taking oral contraceptives (86%). | Infants, exclusively (82%) and partially (18%). | Collection of mature milk 20-60 d pp, both complete breasts for 24h, expressed manually. Stored at 6°C and then -70°C until analysis. | ICP-OES | 136 |
| Mello-Neto 2013 (261) | Cross-sectional study | Brazil | Healthy women 16-41 years, milk donors, with or without iron supplement, primiparous and multiparous, BMI 17-41. |  | Collection of mature milk 20-62 days pp from milk bank, expressed manually, over 24h period, from both breasts, before, during and after feedings. | Spectrometry | 145 |
| Melnikov 2007 (262) | Cross-sectional study | Brazil | Healthy women, mean age 22 years, parity 1-8, non- and smoking (1.7%). | Infants, no malformation, no clinically detectable impairment. | Collection of colostrum milk at 2 d pp, by manual expression. | AAS | 117 |
| Mendelson 1982 (263) | Case-control study | Canada |  | Infants, term (n=10) or preterm (n=14), AGA. | Collection of 24h milk at 3-5, 8-10, 15-17, 28-30 d pp. Stored at -20°C until analysis. | FAAS | 24 |
| Milnerowicz 2003 (264) | Cross-sectional study | Poland | Women 23-39 years, living in heavy metal contaminated area, active (n=10) and passive (n=10) smokers. | Term infants. | Collection of transitional milk (3-8 d pp), once per day, early morning. Stored at -80°C until analysis. | AAS | 20 |
| Mohd-Taufek 2016 (265) | Cross-sectional study | Australia | Milk donors ≥18 years old. |  | Milk from milk donors. Stored at -80°C until analysis. Extracted data pre-pasteurization. | ICP-MS | 16 |
| Moran 1983 (266) | Cohort study | USA | Women, no apparent disease or nutritional inadequacy. | Preterm (<32 weeks) infants. | Collection of milk, weekly until 49 d pp, expressed manually, 24h collection. Stored at -20°C until analysis. | FAAS | 13 |
| Moser 1983 (267) | Cohort study | USA | Women, mean age 28-30±2 years. |  | Collection of mature milk at 1, 3, 6 months pp, first morning feed after 6 AM, fore- and hindmilk. Stored refrigerated and then frozen until analysis. | AAS | 23 |
| Moser 1988 (268) | Cross-sectional study | Nepal | Women, living in Kathmandu valley, consuming mean 10.5 mg zinc/d. | Infants 2-6 months old (mean 3.8 months old), singleton. | Collection of mature milk 2-4 months pp, expressed manually, foremilk, early in the morning. Frozen until analysis. | AAS | 26 |
| Moser-Veillon 1990 (269) | Randomized controlled trial | USA | Healthy women 20-36 years, randomized to 4 different vitamin-mineral supplements with different vitamin B6 and zinc contents (0.6 or 4 mg vitamin B6, 0 or 25 mg zinc) from 1 d to 9 months pp. |  | Collection of transitional and mature milk samples at 1, 2 week, 1, 3, 6, 9 months pp, first feeding in the morning, expressed manually, fore- and hindmilk. Stored at -20°C until analysis. | AAS | 40 |
| Motoyama 2021 (270) | Cohort study | Japan | Healthy women, 34±5 years, BMI 21.7-21.3, without for hypertension or hypothyroidism. | Term infants 1 and 3 months old, singleton, 73% exclusively breastfed. | Collection of milk at 1 and 3 months pp. Stored at -80°C until analysis. | ICP-MS | 79 |
| Murthy 1971 (271) | Cross-sectional study | USA |  |  |  | AAS | 22 |
| Nagra 1989 (116) | Cohort study | Pakistan | Women 27-35 years, medium SES. |  | Collection of mature milk every month 1, 2, 3, 4, 5, 6, 7, 8, 9, 10 , 11, 12 months pp, fore- and hindmilk, 9-11 AM, after eating breakfast. | AAS | 20 |
| Nakamori 2009 (272) | Cross-sectional study | Vietnam | Women 19-37 years old, no medical disorder, no medication, no hormonal contraceptives, in rural area, not pregnancy, only breastfeeding one infant, 25% taking iron-folic acid supplements. | Infants 5.5-12.2 months old, 12% low birth weight. | Collection of mature milk 6-12 months pp, in the morning, expressed by hand. Stored at -20°C until analysis. | ICP-AES | 60 |
| Narang 2006 (273) | Cohort study | India | Women 21-35 years, no chronic illness. | Healthy infants, preterm (n=22 <33 weeks and n=23 33-36 weeks) and term (n=41 37-41 weeks). | Collection of colostrum, transitional and mature milk on 3, 7, 14, 28 d pp, expressed manually. Immediately analyzed. | ICAPS | 100 |
| **Neville 1984b** (117) | Cross-sectional study | USA | Women 18-40 years, primiparous and multiparous, White and Black (n=1). | Healthy full-term infants, exclusively breastfed. | Collection of mature milk 33-210 d pp, expressed with pump. Stored at -70°C until analysis. | FAAS | 12 |
| **Ohtake 1981** (274) | Cohort study | Japan | Healthy women 22-32 years, primiparous (n=14) and multiparous (n=16), well-nourished, no history of serious disease, uncomplicated vaginal delivery. | Healthy full-term infants, singleton, birth weight 2.3-4.3 kg. | Collection of colostrum transitional and mature milk at 1-6 d, 1, 2, 3 months pp, by manual expression, 2-3 PM before feeding. Stored at room temperature and analyzed within 4 days. | AAS | 30 |
| Ohtake 1993 (275) | Cohort study | Japan | Healthy women 19-37 years old, living in urban area, no history of serious disease. | Healthy infants 2-201 days old, normal growth, not all exclusively breastfed. | Collection of colostrum, transitional, mature milk 2-201 d pp, by manual expression, 2-3 PM, before feeding. Stored at room temperature and analyzed within 4 d. | AAS | 80 |
| Okolo 2000 (276) | Cross-sectional study | Nigeria | Healthy women 21-31 years, parity 1-6. | Infants 6-6.5 months old. | Collection of mature milk at 6 months pp. Stored at -20°C until analysis. | ICP-OES | 15 |
| Oliveira 2019 (120) | Cross-sectional study | Brazil | Women 17-44 years, BMI 18-38. | Infants, GA 27-42 weeks. | Mature milk >15 d pp from milk bank. Pasteurized and stored at -20°C for 24h. | FAAS | 50 |
| Ortega 1997 (277) | Cohort study | Spain | Healthy women 18-35 years old, no diabetes, no pre-eclampsia, living in urban area, with zinc intake </>50% RI. | Infants, singleton, normal birth weight >=2.5 kg. | Collection of transitional milk 13-14 d pp and mature milk 40 d pp, 10-11AM, by manual expression, fore- and hindmilk. | AAS | 57 |
| Orun 2012 (278) | Cross-sectional study | Turkey | Women 17-41 years, apparently well-nourished, no cracked nipples, no mastitis, no cream on nipple, primiparous (n=76) and multiparous (n=66), non- and smoking, without and with supplements, vaginal (n=73) and cesarean (n=69) delivery. | Infants, term (n=125) and preterm (n=17), normal (n=33) and low (n=91) birth weight, exclusively (n=116) and predominantly (n=26) breastfed. | Collection of mature milk 52-60 d pp, by manual expression, 2h since last feeding, in the morning. | ICP-MS | 142 |
| Osredkar 2022 (279) | Cohort study | Slovenia | Women, 30±4 y. | Infants 6-8 weeks old. | Collection of milk 6-8 weeks pp. Stored at -20°C until analysis. | ICP-MS | 243 |
| Parr 1991 (122) | Cross-sectional study | Guatemala, Hungary, Nigeria, Philippines, Sweden, Democratic Republic of the Congo | Women from different countries, traditions, SES and nutritional backgrounds. | Infants, 3 months old. | Collection of mature milk (3 months pp) of one complete breast, around noon, approximately 4h after the previous feed, extracted with pump. Frozen at -11°C until analysis. | INAA | 330 |
| **Perrin 2017** (123) | Cohort study | USA |  | Healthy term infant 9-11 months old. | Collection of mature milk monthly 11-17 months pp, one complete breast, first or second feeding of the morning. Stored in freezer and then -80°C until analysis. | ICP-OES | 19 |
| **Picciano 1976** (280) | Cohort study | USA | Women >=20 years, primiparous (n=16) and multiparous (n=34), 74% taking vitamin-mineral supplements (providing <10% RDA zinc). | Healthy full-term infants, exclusively breastfed. | Collection of mature milk 6-12 weeks pp, at early morning feed for 5 consecutive days and 2 additional samples from morning, midday or evening feeds. Stored frozen until analysis. | AAS | 50 |
| Picciano 1981 (124) | Cohort study | USA | Women >=20 years (mean age 25 years), 69% taking vitamin-mineral supplements. | Full-term infants, birth weight 2.8-5.2 kg, exclusively breastfed. | Milk intake was measured at 1, 2, 3 months pp, with 72h test-weighing, at home by mothers. Collection of mature milk at 1, 2, 3 months pp, expressed manually or with manual pump. Stored frozen until analysis. | AAS | 26 |
| Pietrzak-Fiecko 2020 (125) | Cross-sectional study | Poland | Women 21-34 years. |  | Collection of mature milk 3-4 months pp, hindmilk, with pump, one breast. Stored at -20°C until analysis. | FAAS | 18 |
| Qian 2002 (129) | Cross-sectional study | China | Healthy women 18-36 years, non-smoking, no chronic medication, parity of 1-2. |  | Collection of transitional milk. | FAAS | 120 |
| Qian 2010 (130) | Cross-sectional study | China | Healthy women 22-36 years, primiparous, living in residential areas from urban and suburban areas, no chronic illness, consuming normal varied diets. | Healthy full-term infants. | Collection of transitional milk 8-10 d pp, foremilk, 9-11 AM, by manual expression. Stored at -20°C until analysis. | AAS | 120 |
| Qian 2012 (281) | Cross-sectional study | China | Women 18-36 years, uncomplicated pregnancy, primiparous, no disease, no lactation failure. | Infants, singleton, no fetal malformation. | Collection of mature milk 42 d pp, foremilk, in the morning, by manual expression. | AAS | 750 |
| Qian 2022 (131) | Cohort study | China | Healthy women 20-42 years, no chronic diseases, 449 out of 678 had parity of 1, 405 out of 678 had vaginal delivery, |  | Collection of colostrum (0-5 day postpartum) and transitional milk (10-14 day postpatum), and mature milk (40-45 d, 200-240d, and 300-400d postpartum). Stored at -80°C until analysis. | ICP-MS | 678 |
| Queiroz Bortolozo 2004 (132) | Cross-sectional study | Brazil | Milk donors of term and pre-term infants. | Term (n=20) and preterm (<=36 weeks, n=10) infants. | Milk from milk bank, pasteurized. Collection of colostrum and mature milk. | AAS | 30 |
| Rajalakshmi 1980 (282) | Cohort study | India | Women, from low-income urban (n=412), high-income urban (n=100), low-income rural (n=222) populations studied cross-sectionally and low-income (n=24) populations studies longitudinally. |  | Collection of colostrum, transitional and mature milk from 2 d to >13 months pp, expressed manually, before feeding. | FAAS | 758 |
| Rodriguez Rodriguez 2000 (283) | Cross-sectional study | Spain | Healthy women 21-35 years. |  | Collection of mature milk 2 weeks-5 months pp, fore- and hindmilk, several samples representative of the day, with a mechanical pump. | AAS | 11 |
| Rossipal 1998 (284)^[[15]](#footnote-15)^ | Cross-sectional study | Austria | Healthy women, uneventful pregnancies, consumed customary food, not taking trace element supplements. | Term infants. | Collection of colostrum (1-3 d pp), transitional (4-17 d pp) and mature milk (40-293 d pp), 3-4 h since last feeding, from breast not used in last feeding, expressed with electric pump. Stored at -20°C until analysis. | ICP-MS | 44 |
| Sabatier 2019 (136) | Cohort study | Switzerland | Healthy women >18 years, no diabetes, no alcohol or drugs consumption. | Term (<37 weeks) and preterm (28-32 weeks) infants. | Collection of transitional and mature milk at 1, 2, 3, 4, 5, 6, 7, 8 weeks pp for preterm and term infants, and at 10, 12 , 14, 16 weeks pp for preterm infants, from a full single breast, between 6-12 AM, with electric pump. Stored at -18 and then -80°C until analysis. | ICP-MS | 61 |
| **Salmenpera 1994** (286) | Controlled trial | Finland | Healthy women, non-smoking, uncomplicated pregnancy and delivery, allocated to no (66 mg iron), low (20 mg zinc, 2 mg copper, 266 mg iron) or high (40 mg zinc, 4 mg copper, 266 mg iron) daily zinc supplement, all women received multivitamin supplement (vitamin A, D3, E, folate, B1, B2, B6, niacin). | Healthy full-term infants, AGA, Apgar score >=8. | Collection of colostrum (4-5 d pp) and mature milk (2, 4, 6, 7.5, 9, 10, 11, 12 months pp), expressed manually. Stored at -20°C until analysis. | PIXE | 200 |
| Samuel 2014 (287)^[[16]](#footnote-16)^ | Cohort study | India | Women 18-40 years, no illness, primiparous (76%) and multiparous (24%), not from low SES, mean BMI 25±5. | Infants, singleton, mean GA 39±1 weeks. | Collection of mature milk at 1, 3, 6 months pp, mid-feed, from morning, afternoon and evening, expressed by hand. Stored at -20°C until analysis. | FAAS | 50 |
| Samuel 2020 (138) | Cohort study | France, Italy, Norway, Portugal, Romania, Spain, Sweden | Women 18-40 years, BMI 19-29, no condition or medication that contraindicates breastfeeding, with (n=108) or without (n=197) subclinical mastitis. | Infants, mean GA 39-40 ±1 weeks, mean birth weight 3.2-4±0.4-5 kg. | Collection of colostrum and mature milk at 2, 17, 30, 60, 90, 120 days pp, with an electric pump, 9 AM-1 PM, from one complete breast. Stored at -18 and then -80°C until analysis. | ICP-MS | 331 |
| Sann 1981 (139) | Cross-sectional study | France |  | Infants, term (n=61, GA 38-41 weeks) and preterm (n=41, GA 26-35 weeks). | Collection of milk <=6 d, 7-14 d, >=15 d pp, expressed by mechanical or manual methods, 4-6x per day. Stored at 4°C and pasteurized. | Voltametry | 102 |
| Sazawal 2013 (289) | Randomized controlled trial | India | Women, mean age 24-26±4-5 years, no chronic systemic illness, no severe anemia, not requiring vitamin or micronutrient prescription, randomized to multinutrient supplement without or with zinc (40 mg/d) for 1 month. | Infants 0-3 months or 4-6 months old. | Collection of milk at baseline and endline (after 1 month), mid-feed, by manual expression, from both breasts. Stored at -20°C until analysis. | FAAS | 212 |
| Severi 2013 (290) | Cohort study | Uruguay | Women 24-35 years (n=123) and adolescents 13-19 years (n=122), no pathology, no chronic disease. | Infants, singleton, mean GA 39±2 weeks. | Collection of mature milk at 4 months pp, by manual expression. Stored at -20°C until analysis. | FAAS | 245 |
| Shaaban 2005 (291) | Randomized controlled trial | Egypt | Healthy women, primiparous, no history of medical or obstetric illness, randomized to daily multivitamin supplement with or without zinc (10 mg/d) for 2 months. | Healthy full-term infants, singleton, exclusively breastfed, no neonatal intensive care admission needed. | Collection of milk at baseline/delivery and after 2 months supplementation/pp, 10AM and 10PM. | FAAS | 60 |
| **Sharda 1983** (292) | Cross-sectional study | India | Healthy women, no evidence of undernutrition, vitamin deficiency or anemia. | Term infants. | Collection of colostrum (2-5 d pp), transitional (6-10 d pp) and mature (>10 d pp) milk, in morning, foremilk, by manual expression. | AAS | 178 |
| Sharda 1999 (293) | Cross-sectional study | India |  | Infants, preterm and term, 26-41 weeks gestational age, AGA and SGA, birthweight 0.6-3.8 kg. | Collection of colostrum milk, from both breasts, by mechanical expression, at variable intervals. | AAS | 155 |
| Shawahna 2022 (294) | Cross-sectional study | Palestine | Women, ≥18 y. |  | Collection of foremilk, with manual expression, 9-11 AM. Stored at -20°C until analysis. | ICP-MS | 390 |
| Shi 2011 (143) | Cohort study | China | Healthy women, mean age 28 years, mainly primigravida, non-vegetarian. | Healthy full-term infants. | Collection of colostrum, transitional, and mature milk, from both breasts, after lunch (2nd feed of the day), extracted with pump. Stored at -20°C until analysis. | AAS | 80 |
| Sian 2002 (295) | Cross-sectional study | China | Healthy women, mean BMI 24.3, from rural area, no acute or chronic disease, non-smoking, no alcohol, no supplements. | Infants 1-2 months old, exclusively breastfed, no acute or chronic disease. | Collection of mature milk sample 1-2 months pp, by hand expression, midfeed, from one breast. Stored at -20°C until analysis. | FAAS | 18 |
| Sievers 1992 (296) | Cohort study | Germany | Healthy women 26-35 years, primi- and multiparous. | Term infants, breastfed. | Collection of mature milk at 17, 35, 57, 85, 113 ±3 days pp, fore- and hindmilk from side used for nursing. Stored at -20°C until analysis. | FAAS | 10 |
| Silva 1997 (144) | Cross-sectional study | Brazil | Women, from city of Brasilia. |  | Collection of milk at different stages of lactation, by manual expression. Stored at -20°C until analysis. | ICP-AES | 203 |
| Silvestre 2000a (297) | Cross-sectional study | Spain |  |  | Collection of colostrum, transitional and mature (30, 60, 90 d pp) milk. Stored at -18°C until analysis. | FAAS | 50 |
| Silvestre 2000b (298) | Cohort study | Spain | Healthy women, well-nourished, primiparous and multiparous, non- and smoking, iron supplements 0-160 mg/d. | Infants, AGA. | Collection of colostrum and transitional milk 3-15 d pp, with automatic breast pump. Stored at -18°C until analysis. | FAAS | 62 |
| Silvestre 2001 (299) | Cohort study | Spain | Healthy women, well-nourished. | Full-term infants, AGA. | Collection of colostrum (2-4 d pp), transitional (14 d pp), mature milk (30, 60, 90 d pp), from both breasts, 11AM-4PM, with electric pump. Stored at -18°C until analysis. | FAAS | 39 |
| Simmer 1990 (300) | Cohort study | Bangladesh |  |  | Collection of mature milk at 1, 2-3, 6, 9, 12 months pp, 10AM-2PM, from both breasts, fore- and hindmilk. Stored at -20°C until analysis. | AAS | 34 |
| Snoj Tratnik 2019 (301) | Cross-sectional study | Slovenia | Healthy women 18-49 years, primiparous, no problem during pregnancy, no living within 100-200m of heavy metal contamination sites. | Healthy infants 2 weeks-7 months old, singleton. | Collection of mature milk 2 weeks-7 months pp (73% 6-8 weeks pp), from multiple feeds in maximum 6 days. Stored at -20°C until analysis.. | ICP-MS | 536 |
| Szukalska 2021 (146) | Case-control study | Poland | Healthy women 18-40 y, non-smoking, exposed to second-hand smoke or smoking. |  | Collection of colostrum (0-2 d pp) and mature milk (23-37 d pp), by electronic pump. Stored at -80°C until analysis. | ICP-MS | 150 |
| Tahboub 2021 (302) | Cross-sectional study | Jordan |  |  | Collection of milk with a breast pump. Stored at -20°C until analysis. | ICP-MS | 76 |
| Trugo 1998 (303) | Cross-sectional study | Brazil | Women 16-40y, from low SES, no supplementation during pregnancy. | Term and preterm infants, AGA, exclusively breastfed. | Collection of colostrum/transitional milk (0-36 d pp), 9-10 AM, by manual expression, before feeding infant. Stored at -20°C until analysis. | AAS | 52 |
| **Taravati Javad 2018** (147) | Cross-sectional study | Iran | Healthy women 19-42 years, no supplements, non-vegetarian, no medication to enhance milk production. | Full-term infants, normal birth weight, exclusively breastfed, no chronic disease. | Collection of mature milk at 1, 2, 6, 7, 12 months pp, in the morning, more than 1h since last feed, expressed with manual pump. Stored at 4 and the -20°C until analysis. | ICP-MS | 100 |
| Tripathi 1999 (304) | Cross-sectional study | India |  |  |  | FAAS | 30 |
| Turan 2001 (305) | Cross-sectional study | Turkey | Women 18-33 years, from middle-class, non-smokers and smokers, with and without supplementation. |  | Collection of colostrum, extracted with pump. Stored in fridge until analysis. | FAAS | 30 |
| Umeta 2003 (151) | Cross-sectional study | Ethiopia | Women, mean age 26-27±0.4-6 years, BMI 20±0.1-0.2, in rural area. | Infants 5-11 months old, not stunted. | Collection of mature milk, from right breast 1h after last feeding. Stored -20°C until analysis. | AAS | 195 |
| Ustundag 2005 (306) | Cohort study | Turkey | Healthy women, mean age 21-23±1-2, non-smoking, no anti-inflammatory medication. | Preterm and term infants. | Collection of colostrum (0-7 d pp), transitional (7-14 d pp) and mature (21 and 60 d pp) milk, from one breast, 2h since first feeding of the morning, 8-11AM. Stored at 4 and then -40°C. | FAAS | 40 |
| Van der Elst 1986 (307) | Case-control study | South Africa | Women, thin BMI 17-22 (n=30) and non-thin BMI 24-28 (n=30), uneventful pregnancy and delivery. | Infants, AGA, clinically normal, breastfed for at least 21 days. | Collection of mature milk 19-21 d pp. | AAS | 60 |
| Vanderja 2002 (153)^[[17]](#footnote-17)^ | Cross-sectional study | Nigeria | Women 15-45 years, from Fulani ethnicity, parity 1-10, BMI 14.5-24. | Infants 2-24 weeks old (mean age 11 weeks). | Collection of mature milk 2-24 weeks pp, by manual expression, mid-feed. Stored at 20°C and then frozen until analysis. | ICP-OES | 34 |
| **Vaughan 1979** (155) | Cohort study | USA | Women, Caucasian, good or excellent nutritional status, no complication during pregnancy or delivery, primiparous (n=22) and multiparous (n=16). | Full-term infants. | Collection of mature milk monthly 1-31 months pp, expressed by hand, over 3-5 days in morning, afternoon and evening feeds, randomly during feed. Stored frozen until analysis. | AAS | 38 |
| Vuori 1979 (308) | Cohort study | Finland | Healthy women 20-35 years, well-nourished, primiparous. |  | Collection of mature milk from 2 weeks to 9 months pp, fore- and hindmilk during 24h. Stored frozen until analysis. | FAAS | 27 |
| Vuori 1980 (309) | Cohort study | Finland | Women 24-35 years old, from high SES, 80% used iron supplements. |  | Collection of mature milk at 6-8 and 17-22 weeks pp, fore- and hindmilk, all feeds during 24h. | FAAS | 15 |
| Wang 2001 (310) | Cross-sectional study | China | Healthy women 22-32 years of age. | Healthy full-term infants. | Collection of sample from women at 5 different lactating stages. | AAS | 193 |
| Wang 2007 (158) | Cross-sectional study | China | Healthy women 18-38 years with no recent supplementation of calcium, magnesium, manganese, or iron. |  | Collection of mature milk 38-510 d pp, from one breast, in the morning, 7-10 AM. Stored at -70°C until analysis. | AAS | 55 |
| Wasowicz 2001 (311) | Cross-sectional study | Poland | Healthy women, uncomplicated pregnancy and delivery, no microelement supplementation during pregnancy. | Full-term infants. | Collection of colostrum, transitional, mature milk 1-30 d pp, expressed by hand. Stored at -20°C until analysis. | ICP-AES | 131 |
| Wei 2020 (159) | Cross-sectional study | China | Healthy women 20-40 years, from different geographical regions of China. |  | Collection of colostrum (3-6 d pp), transitional (10-13 d pp) and mature (21-25 d pp), from second feeding in the morning (9 AM). Stored -20°C and the -80°C until analysis | GB-AAS | 175 |
| Winiarska-Mieczan 2014 (312) | Cross-sectional study | Poland | Healthy women 20-40 years, 13% smoking. | Infants 1-12 months old. | Collection of mature milk 1-12 months pp, by manual expression. Stored at -20°C until analysis. | FAAS | 320 |
| Wu 2020 (313) | Case-control study | China | Healthy women, mean age 28-29±4 years, 83-86% primiparous, 1-2% smoking, no extracorporeal fertilization, vaginal (n=617) or cesarean (n=626) delivery. | Healthy full-term infants, singleton, 1-min Apgar score >=8, mean birth weight 3.2-4±0.4 kg. | Collection of colostrum milk 3-5 d pp, 8-10 AM. Stored at -20°C until analysis. | ICP-MS | 1243 |
| **Xiang 2007** (314) | Cross-sectional study | China | Healthy women 18-36 years, non-smoking, no chronic medication, parity of 1 or 2. | Healthy full-term infants, 1 and 3 months old, exclusively breastfed. | Collection of mature milk, 5 samples during the day, 2 min after start of feed, extracted by hand . Stored at 4°C for max 10h and then at -20°C until analysis. | AAS | 41 |
| **Yalcin 2010** (315)^[[18]](#footnote-18)^ | Cross-sectional study | Turkey | Healthy women 18-38 years, BMI 18-31, normal and healthy pregnancy, normal blood pressure, no gestational hypertension, vaginal and cesarean (n=3) delivery. | Term infants, birth weight >=2.5 kg, singleton, exclusively breastfed until 5 months of age at least. | Collection of transitional/mature milk 10-20 d pp, in the morning before feeding, from both breasts, expressed by hand. Stored at -20°C until analysis. | AAS | 44 |
| Yamawaki 2005 (160) | Cross-sectional study | Japan | Women <40 years, non-smoking, no vitamin supplements. | Infants, birth weight >=2.5 kg, no symptoms of atopy. | Collection of colostrum/transitional/mature milk 1-365 d pp, mid-feed. Stored at -20 and then -40°C until analysis. | ICP-AES | 1197 |
| Yoshinaga 1991 (162) | Cross-sectional study | Japan | Women, mean age 28±5 years, mean BMI 22±2. | Infants, mean birth weight 3.1±0.4 kg. | Collection of transitional (5-9 d pp) milk, by manual expression. Stored at -20°C until analysis. | ICP-AES | 51 |
| Young 2019 (317) | Cross-sectional study | USA | Human milk donors, dairy-free diet (4%) or not (96%). | Infants, term (n=174) and preterm (n=38). | Milk from Mothers Milk Bank from 3 d to 9.8 months pp, after pasteurization. | AAS | 138 |
| **Zapata 1994** (318) | Controlled trial | Brazil | Women 19-35 years (mean 27 years), from low SES, had prenatal care, received iron supplement during second half of pregnancy, uncomplicated pregnancy and delivery, non-smoking, iron sufficient, no mastitis, no infection, assigned to 40 mg/d iron supplement (n=14) or no supplement (n=14) from 1-2 d pp for 90 days, no other supplement, average intake 11 mg/d zinc (range: 5-18 mg/d) | Healthy full-term infants, exclusively breastfed. | Collection of milk, 8-10 AM, by manual expression, both breasts. Stored at -20°C until analysis. | FAAS | 28 |
| Zavaleta 1995 (319) | Cross-sectional study | Peru | Healthy women 18-44 years, BMI mean 25±4 range >19.5, from low SES. Data from women with acute febrile infection were not used. | Healthy infants 1-6 months old, exclusively breastfed. | Collection of mature milk 1-6 months pp, on 3 days (1, 7, 14). Stored at -20°C until analysis. | FAAS | 38 |
| Zhao 2011 (163) | Cross-sectional study | China | Healthy women with mean age of 26.36, primigravida, non-smoking, no medication history. | Infants, exclusively breastfed. | Collection of milk samples, Stored at -20°C until analysis. | ICP-MS | 39 |
| **Zhao 2014** (164) | Cross-sectional study | China | Healthy women 18-45 years old, living in 3 cities of China, without diabetes, hypertension or other chronic or acute disease, no nipple or lacteal gland disease, no use of hormone, no postpartum depression or other mental disease, 96% Han ethnicity, mean BMI 23, vaginal and cesarean delivery. | Health full-term infants, singleton. | Collection of milk at 5-11, 12-30, 31-60, 61-120, 121-240 d pp, from one complete breast, 9-11 AM, with electric pump. Stored at -80°C until analysis. | ICP-MS | 444 |

Note : Studies included in selection of key studies in **bold**.

# ADDITIONAL FILE 4. Characteristics of studies included for breast milk VITAMIN D concentration

| **ID [Reference]** | **Study design** | **Country** | **Mother description** | **Child description** | **Milk description** | **Analytical method desription** | **Sample size** |
| --- | --- | --- | --- | --- | --- | --- | --- |
| Ala-Houhala 1988 (320) | Controlled trial | Finland | Women, supplemented with 0, 1000 (25 ug) or 2000 (50 ug) IU vitamin D2 or D3/d from birth to 15/20 weeks (2 experiments). | Infants born in winter or summer. | Collection of mature milk at 8 and 15/20 weeks pp, fore- and/or only hindmilk, first morning feed, during winter and summer. Stored at -70°C until analysis. | Vitamin D and 25OHD were measured with HPLC and CPBA. Method described in (321). Standards were obtained. Intra- and inter-assay CV were <15%. | 85 |
| Amukele 2013 (322) | Cohort study | Malawi | Women, HIV infected. | Infants, HIV uninfected. | Collection of milk at birth, 6-8 weeks, 12, 15, 18, 24 months pp. | Total 25OHD, 25OHD2, 25OHD3 was measured with LC-MS/MS. Method described in (323). Accuracy of 102-105% compared with standards. LOD 0.1 ng/mL. All samples had concentrations below detection. | 21 |
| Atkinson 1987 (324) | Cohort study | Canada | Women, with minimum vitamin D intake of 400 IU/d (from diet and supplements). | Infants, preterm (n=8 and 6, GA 24-32 weeks) and term (n=9, GA >=37 weeks). | Collection of transitional/mature milk at 14-21 d, and 29-32 (substudy) pp, complete 24h expressions, with manual or electric pump. | Vitamin D2, vitamin D3, 25OHD2, 25OHD3 were measured with HPLC and CPBA. Method described in (325). | 23 |
| Ballester 1987 (326) | Cross-sectional study | Spain | Women from different social classes, with different nutritional status, nutritional supplements, and sun exposure. | Term infants. | Collection of milk samples in late autumn and early spring. | Vitamin D was measured with HPLC and CPBA. Labeled vitamin D3 was added and recovery was 65-75%. Intra-assay error was 6-16% and inter-assay variation led to similar means. The method was compared with (325) and provided similar values. | 14 |
| Cancela 1986 (34) | Cohort study | France | Women, mainly African origin, not taking vitamin D supplements (all except one). | Full-term infants, 0-1 month. | Collection of colostrum (3-5d pp), transitional (15-18d pp), and mature milk (30-45d pp), extracted with manual pump. Stored at -20°C until analysis. | Vitamin D was measured with HPLC and CPBA. Method described in (325,327). Labeled vitamin D3 and 25OHD3 were added and recovery was 60% and 70-80%. Intra- and inter-assay CV were ≤15%. | 13 |
| Dawodu 2019 (328) | Randomized controlled trial | Qatar | Healthy women, mean age 30 years, mean BMI 29, Arab, no calcium disorders, no thyroid disease, no type 1 diabetes, no liver disease, randomized to vitamin D supplements to mother only (6000 IU/d) or to mother (600 IU/d) and infant (400 IU/d). | Term infants, exclusively breastfed 4-6 months, no congenital anomalies. | Collection of mature milk at 4 weeks, 4 months, and 7 months pp. Stored frozen until analysis. | Vitamin D was measured with LC-MS/MS, after purification with HPLC. Method described in (329). Inter- and intra-assay CV ≤12%. LOD was 10 pg/mL. At baseline, 57% had concentrations below detection. | 190 |
| Demers-Mathieu 2021 (330) | Case-control study | USA | Women with COVID-19, with viral symptoms or with no infection, 23-40 years old, 75% took prenatal vitamins. | Infants 2-13 months old. | Collection of milk 2-13 months pp, 50% of samples collected during winter. | Vitamin D3 was measured with ELISA kit (MyBioSource). | 40 |
| Ferreiro-Vera 2013 (331) | Cross-sectional study | Spain | Healthy women, not taking medication, not taking supplementary vitamins. |  |  | Vitamin D was measured with LC-MS/MS system with a triple quadrupole mass spectrometer (experimental set-up). Used standards and found recoveries for vitamin D2, D3, 25OHD2, 25OHD3 85-100%. Vitamin D2 and 25OHD2 were below detection. In discussion, mentions that values are higher than (325). | 5 |
| Gates 2021 (60) | Cohort study | USA | Women, 18-37 y, not vegan or vegetarian, not restricting caloric intake, HIV negative. | Preterm (≤33 GA) infants. | Collection of milk at 7, 14, 21 and 28 d pp, with electric breastpump, 24h collection, emptying both breasts every 3h. Stored at -29°C until analysis. | Vitamin D was measured with UV-HPLC. | 36 |
| Gjerde 2020 (332) | Randomized controlled trial | Norway | Women, primiparous, no fish allergy, no chronic disease that affects iodine status, who were randomized to receiving 400g cod/week or not for 16 weeks during pregnancy. | Infants, preterm and fullterm, singleton. | Collection of mature milk at 6 weeks and 3 months pp, from beginning, middle and end of feed. Stored at -80°C until analysis. | Vitamin D was measured with LC-MS/MS with Agilent 1290 UPLC. Validation study, studied linearity, different levels, LOQ and LOD. Intra-assay CV ≤16% and inter-assay CV ≤30%. Recovery 72-101%. | 137 |
| Gomes 2016 (333) | Cross-sectional study | Australia | Healthy women, milk donors. |  | Stored at -80°C until analysis. Extracted values pre-pasteurization. | Vitamin D2, D3, 25OHD2, 25OHD3 were measured with LC-MS/MS. Method described in (334). Repeatability/intra-assay CV was ≤13% and recovery 94-99%. Used standards to calibrate method. | 16 |
| Greer 1981 (335) | Cross-sectional study | USA | Women, Caucasian, some tool vitamin D supplements (400 IU) (n=3). | Infants, term (n=3) and preterm (n=3), 3-168 days old. | Collection of fore-, mid- and hind-milk from first feed of the day, at 3-168 d pp, during summer. | 25OHD was measured with HPLC and CPBA. HPLC had LOD of 0.5 ng/mL, CV of 15%, and recovery 60%. CPBA, method described in Haddad and Chyu 1971, had a sensitivity of 0.025 ng and CV of 15%. All samples had concentrations below detection level. | 6 |
| Greer 1984 (336) | Trial | USA | Healthy women 26-35 years, White, middle SES, exposed to 1.5 MED UVB, averaging 88 second irradiation. Data from baseline were used. |  | Collection of mature milk 2-10 months pp, after 0h, 24h, 48h, 7d, 14d after UVB exposure. | Vitamin D3, vitamin D2, 25OHD3, 25OHD2 were measured with CPBA and HPLC. Method described in (325,327). | 5 |
| Hollis 1986 (337) | Cross-sectional study | USA | Normal women, consuming approximately 400 IU vitamin D/day. |  | Collection of mature milk from 2 weeks to 3 months pp. Stored at -20°C until analysis. | Vitamin D2, vitamin D3, 25OHD2, and 25OHD3 were measured with HPLC and CPBA. Method described in (325,327). | 51 |
| Hollis 2004 (338) | Randomized controlled trial | USA | Women, mean age 29-31±5-6 years, 73% White and 27% African American, no preexisting diabetes type 1 or 2, no hypertension, no parathyroid disease or uncontrolled disease, received 2 different vitamin D regimens (200 IU/d vs 400 IU/d) | Infants, exclusively breastfed, mean birth weight 3.4-3.5 kg, GA 36-41. | Collection of mature milk (1, 2, 3, 4 months pp). | Vitamin D2, vitamin D3, 25OHD2, 25OHD3 were measured with HPLC and CPBA. Method described in (327,339,340). | 18 |
| Hoogenboezem 1989 (341) | Cross-sectional study | Netherlands | Healthy women, Caucasian. | Term infants, exclusively breastfed. | Collection of transitional milk 1, 2, 3, 4, 8, 13, 21 weeks pp, matching breast. Stored at -20°C until analysis. | Vitamin D was measured with HPLC and CPBA. Used standards and tracers. Assay sensitivity of 12 pg/tube. Intra- and inter-assay CV of 16 and 22%. | 39 |
| Kamao 2007a (342) | Cross-sectional study | Japan | Women, mean age 31±4 years. |  | Collection of colostrum/transitional/mature milk 1.5 d to 1.2 months pp. | Vitamin D2, vitamin D3, 25OHD2, and 25OHD3 were measured with LC-APCI/MS/MS. Development of method in plasma. Used internal standard. Recoveries were 98-104%. Intra- and inter-assay CV were ≤6% and ≤5%. Compared method with DiaSorin RIA kit. | 51 |
| Kamao 2007b (343) | Cross-sectional study | Japan | Women 18-39 years. |  | Collection of colostrum/transitional/mature milk 3-265 d pp (mean 49 d pp), by manual expression, midfeed. Stored at -20°C until analysis. | Vitamin D2, vitamin D3, 25OHD2, and 25OHD3 were measured with LC-MS/MS. Standards were obtained. Recovery 91-105%. Inter-assay CV ≤12%. LOD was 1-2 pg/20 mL. | 82 |
| Khaghani 2008 (344) | Cohort study | Iran | Healthy women, assigned to hormonal (n=52) or non-hormonal (n=86) contraception of their choice, no vitamin D supplementation. |  | Collection of mature milk at 6 months pp, before feeding. Stored at -20°C until analysis. | Vitamin D was measured with RIA method. | 138 |
| Mohamed 2014 (345) | Cohort study | Malaysia | Women 19-40 years, Malaysian or Malay ethnicity, no pre-existing chronic disease, no pregnancy complication, multiparous (42%) and primiparous, normal BMI (58%), <30% with multivitamin supplement during pregnancy. | Term infants, singleton. | Collection of colostrum/transitional and mature milk 1-14 d pp, 2, 6, 12, months pp, with electric breast pump. Stored at -80°C until analysis. | Vitamin D was measured with HPLC. Method described in (346). LOQ was 2 nmol/L. Used internal standards. | 102 |
| Moya 2000 (347) | Cross-sectional study | Spain | Healthy women, eating at least a portion of fish per week (n=86) or never or less (n=16). |  | Collection of mature milk (>15 d pp). | Vitamin D and 25OHD was measured with HPLC and CPBA. | 102 |
| Nguyen 2020 (348) | Cross-sectional study | China, Korea, Pakistan, Vietnam |  |  |  | Vitamin D2 and vitamin D3 was measured with UV-HPLC. External standards were obtained. LOD were 0.5 and 1 µg/L respectively. All samples had concentrations below detection. | 580 |
| Niramitmahapanya 2017 (349) | Randomized controlled trial | Thailand | Healthy women, mean age 27±5 years, mean BMI 22±5, serum 25OHD level 25-75 nmol/L during 3rd trimester of pregnancy, randomized to vitamin D supplement (1800 IU/d) or placebo for 6 weeks. | Term infants, singleton. | Collection of mature milk at delivery and 6 weeks pp. Stored at -80°C until analysis. | 25OHD was measured with HPLC. | 68 |
| Nishimura 2003 (350) | Case-control study | Japan | Women, long-term hospitalized (n=4) for potential premature delivery or control (n=2), primi- and multiparous. | Infants, singleton and multiples, gestational age 34-41 weeks, no fetal distress or cord coiling. | Collection of milk within a week pp. | 25OHD was measured with HPLC. Method described in (351). | 6 |
| Oberhelman 2013 (352) | Randomized controlled trial | USA | Healthy women >=18 years old, non-pregnant, no travel south of 35°N, no indoor tanning, not taking medication that can affect vitamin D metabolism, breastfeeding only one child, not taking supplements >1000 mg calcium or >400 IU vitamin D3, baseline serum 25OHD >175 nmol/L, no hypercalcemia or hyperphosphatemia, no history of nephrolithiasis, randomized to receive vitamin D3 5000 IU/d for 28 d or 150000 IU once. | Healthy infants 1-6 months old, singleton, exclusively breastfed, weight >1.67 kg, baseline serum 25OHD >175 nmol/L, no hypercalcemia or hyperphosphatemia, no serious health problem. | Collection of mature milk 1-6 months pp, on days 0, 1, 3, 7, 14, 28 of intervention. | Vitamin D was measured with isotope-dilution LC-MS/MS. Method described in (353). Assays were standardized against National Institute of Standards and Technology reference material. Intra- and inter-assay CV were 8.0% and 6.1%. Recovery and linearity were 104% and 100%. LOD and LOQ were 0.96 and 7.0 ng/mL. Vitamin D3 was below quantification in baseline samples. 25OHD was undetectable in all samples. | 40 |
| Oberson 2020 (354) | Cross-sectional study | USA | Milk donors. |  | Commercial human milk samples. Stored -20 and -80°C until analysis. | Vitamin D was measured with SFC-MS/MS. Development of method. Validation with a standard reference material (SRM 1849a Infant/Adult Formula) from the National Institute of Standards and Technology with certified vitamin D3 value. LOQ 50 pmol/L. Intra- and inter-assay CV ≤15% and ≤20%. Recovery 85-115%. All samples had 25OHD2 concentrations below detection. | 7 |
| Olafsdottir 2001 (355) | Cross-sectional study | Iceland | Women, Icelandic, used to Icelandic dietary habits, living in Iceland for at least 15 years, taking or not cod liver oil supplement. | Infants 2-4 months old, singleton. | Collection of mature milk 2-4 months pp, in glass bottle, expressed manually or with pump, at all times of the day, discarded first drops. Stored at -20°C, freeze-dried and stored at -70°C. | 25OHD was measured with CPBA test kit developed for plasma, serum and urine from Immundiagnostik. Reconstituted lyophilised samples from breast milk. Vitamin D concentration was calculated with logarithmic linear regression. CV was 12.5%. Some samples had undetectables levels of 25OHD. | 77 |
| Panagos 2016 (356) | Case-control study | USA | Women, mean age 30-31±4-5 years, lean (n=21) and obese (n=21), non-smoking, majority Caucasian, with (n=1) and without gestational diabetes, vaginal and cesarean (n=6) delivery. | Term (>35 weeks) infants, singleton, no intrauterine growth restriction, no fetal anomaly, no fetal demise, exclusively (n=25) and partially breastfed. | Collection of mature milk 4-10 weeks pp, complete feed, in the morning, with pump. Stored at -80°C until analysis. | 25OHD was measured after extraction by an equilibrium 125I radioimmunoassay procedure (DiaSorin, Stillwater, MN, USA) using a Packard Cobra II Gamma Counter, with method described in (357), a kit intended for measurement in plasma, serum, and urine. Intra- and inter-assay CV were 9 and 11%. 25OHD is reported in 74% of the samples (probably 26% undetectable). | 42 |
| Saadi 2009 (358) | Randomized controlled trial | United Arab Emirates | Healthy women, Arabs (n=76) and South Asian (n=14), randomized to vitamin D2 supplementation 2000 IU/d or 60000IU/month, all received 600 mg calcium/d. | Infants, singleton (n=88) and twins (n=4), mean 20 days old at baseline. | Collection of milk at baseline and after 3 months of vitamin D supplementation, from full breast. Stored frozen until analysis. | Vitamin D ARA of milk was measured with HPLC and CPBA. Method described in (327). LOD <20 IU/L. Vitamin D ARA was undetectable in all samples at baseline and in one sample at 3 months. | 90 |
| Sakurai 2005 (359) | Cross-sectional study | Japan | Healthy women <40 years old (mean age 29±4 years), non-smoking, no vitamin supplements. | Infants, birth weight >=2.5 kg (mean birth weight 3.1±0.4 kg), no symptoms of atopy. | Collection of colostrum, transitional and mature milk 1-365 d pp, in summer and winter, mid-feed. Stored in freezer, at -20, and then -40°C until analysis. | Vitamin D3 was measured with UV-HPLC. Used standards. Measured according to methods of "Standard Methods of Analysis for Hygienic Chemists" and modified method of (360). | 691 |
| Shi 2011 (143) | Cohort study | China | Healthy women, mean age 28 years, mainly primigravida, non-vegetarian. | Healthy full-term infants. | Collection of colostrum, transitional, and mature milk, from both breasts, after lunch (2nd feed of the day), extracted with pump. Stored at -20°C until analysis. | Vitamin D was measured with HPLC. Method described in (361). 75% of the samples had concentrations below detection. | 80 |
| Specker 1985 (362) | Cross-sectional study | USA | Women, white (n=15) and black (n=10), during fall-winter (n=12) and spring-summer (n=13), from different SES, taking vitamin D2 supplements (0-706 IU/d). | Term infants, exclusively breastfed, not taking vitamins, no major congenital anomalies, no bone disorder, no gastrointestinal tract disease. | Collection of milk, during first daylight feeding, fore-, mid-, and hindmilk. Stored frozen until analysis. | Vitamin D2, D3, 25OHD2, 25OHD3 were measured with HPLC and CPBA. Method described in (327). Intra- and inter-assay CV <10%. | 25 |
| Stoutjesdijk 2017 (363) | Cross-sectional study | Curacao, Malaysia, Netherlands, Tanzania, Vietnam | Apparently healthy women, well-nourished, no pregnancy complication, all women in The Netherlands and one in Curaçao took vitamin D supplements (10 ug/d). | Apparently healthy term infants 0-6 months old. | Collection of milk 0-6 months pp, one complete breast or midfeed sample around noon or in the morning or undefined time. Stored at -20 or -80°C until analysis. | Vitamin D was measured with LC-MS/MS after saponification and extraction, derivatisation by DMEQ-Tad, as described in (343). Inter- and intra-assay CV were <15 and <10%. LOQ 0.1 nmol/L for vitamin D3 and D2 and 0.2 nmol/L for 25OHD2 and 25OHD3. Vitamin D2 and 25OHD2 were generally below detection. | 181 |
| Stoutjesdijk 2019 (364) | Randomized controlled trial | Netherlands | Healthy women 21-38 years, BMI 18-29, parity 0-2, no hyperemesis gravidarum, no vegetarian/vegan diet, pre-pregnancy BMI <30, no pregnancy complication, randomized to 10, 35, 60, 85 ug vitamin D3/d (400, 1400, 2760, 3400 IU/d) from 20 gestational weeks to 4 weeks pp, all received fish oil and multivitamin supplements. | Term infants, GA 37-42 weeks, birth weight 2.4-5.0 kg, singleton. | Collection of mature milk at 4 weeks pp (endline), full single breast, 10AM-14PM, expression manually or with pump. Stored in freezer and then -20°C until analysis. | Vitamin D was measured with LC-MS/MS after saponification and extraction, derivatisation by DMEQ-Tad, as described in (343). Inter- and intra-assay CV were <15 and <10%. LOQ 0.1 nmol/L for vitamin D3 and D2 and 0.2 nmol/L for 25OHD2 and 25OHD3. Vitamin D2 and 25OHD2 were undetectable in nearly all samples. | 43 |
| Streym 2016 (365) | Cohort study | Denmark | Healthy women 24-41 years, normal uncomplicated pregnancy, Caucasian, 54-79% used vitamin D supplements (5-10 ug/d). | Healthy infants. | Collection of transitional and mature milk at 2 weeks, 4, 9 months pp, fore- and hindmilk, manually expressed, during winter and summer. Stored at 4 and then -80°C until analysis. | Vitamin D2, D2, 25OHD2 and 25OHD3 were measured with LC-MS/MS, after saponification and extraction. CVs ≤8%. LOD 0.2 nmol/L. Samples with concentrations below LOD were assigned a value of 0.14 nmol/L. Vitamin D2 and 25OHD2 were detectable only in 1 and 5 samples respectively. Vitamin D concentrations were below detection in 40% of the milk samples. | 107 |
| Takeuchi 1989 (351) | Controlled trial | Japan | Healthy women, assigned to daily vitamin D2 supplement (1200 IU/d) or none for 4 weeks, starting at 1 week pp. | Term infants. | Collection of milk at 1 and 5 week pp, expressed manually, entire breast, in the morning, before suckling, during winter. Stored at -20°C until analysis. | Vitamin D2, vitamin D3, 25OHD2, 25OHD3 were measured with HPLC. Method described in (366). The values below detection were attributed a value of 0. | 50 |
| Tsugawa 2021 (367) | Cross-sectional study | Japan | Women, mean age 28-30±4 y, from 3 regions in Japan, in 1989 and 2016-2017. |  | Collection of mid- and hindmilk in summer and winter, with manual expression. Stored at -80°C until analysis. | Vitamin D2, D3, 25OHD2 and 25OHD3 were measured with LC-MS/MS. LOD was 0.001 nmol/L. Undetectables (5-82%) were attributed half the LOD. | 162 |
| Van Zoeren-Grobben 1987 (367,368) | Cross-sectional study | Netherlands | Healthy women, no medication, no vitamin supplements | Term infants, breastfed for 1-8 months. | Collection of mature milk 1-8 months pp, expressed with hand pump. Stored at -20°C until analysis. | 25OHD was measured in the lipid extract (where there is ≥75% of the vitamin D ARA of milk) with HPLC and CPBA. Method described in (369). | 8 |
| Wagner 2006 (370) | Randomized controlled trial | USA | Women, mean age 28-30±3-6 years, no diabetes, no hypertension, no parathyroid disease, no uncontrolled thyroid disease, 67-89% White, 11% African American, 0-22% Hispanic, parity 1-6, met dietary recommended intakes for protein, fat, carbohydrate, calcium, phosphorus, magnesium and vitamin D, randomized to 400 (n=10) or 6400 (n=9) IU/d vitamin D3 for 6 months (from 1 to 7 months pp). | Infants maximum 1 month old at baseline, mean birth weight 3.4-6±0.3-4 kg, mean GA 39±1 weeks. | Collection of mature milk at 1 (baseline), 2, 3, 4, 5, 6, 7 months pp. | Vitamin D3 and 25OHD3 was measured with HPLC and CBPA and converted to ARA. Method described in (327,339,340). | 19 |
| Wall 2016 (329) | Randomized controlled trial | New Zealand | Women, mean age 27-29±6 years, European 12-23%, Maori 23-25%, Pacific 34-47%, Other 16-20%, randomized to 0 (n=24), 1000 (n=28), 2000 (n=23) IU/d vitamin D3 for 2 months (from 2 weeks to 2 months pp). | Infants, GA 38-40 weeks, duration of exclusive breastfeed 4-12 weeks. | Collection of mature milk at 2 weeks (baseline) and 2 months (endline) pp, expressed manually or with pump, 2h since last feeding, in the morning, one full breast. Stored at -80°C until analysis. | Vitamin D2, D3, 25OHD2, 25OHD3 were measured with LC-MS/MS. Limit of detection was 10 pg/mL. Method described in (327). Calibration curves were constructed. Linearity close to 1.00. Inter- and intra-assay CV were ≤12%. LOD was 10 pg/mL. ARA was calculated. The concentrations in vitamin D2 and 25OHD2 were below detection. | 75 |
| Wang 2020 (371) | Case-control study | Taiwan |  | Infants 2-4 months old, exclusively breastfed, with atopic dermatitis (n=45) or healthy controls (n=45). | Collection of milk, expressed by hand, after morning feed, 8-11 AM. Stored at -80°C until analysis. | 25OHD3 was measured with UPLC-TQ/MS. Mentioned standards. | 90 |
| Weisman 1982 (372) | Cross-sectional study | Israel | Women, not receiving vitamin D supplement or drugs that may affect vitamin D metabolism. |  | Collection of milk 3-21 d pp, expressed manually or with breast pump. Stored at -20°C until analysis. | 25OHD was measured with CPBA and 25OHD3 was also measured with UV-HPLC. With internal standards, recoveries were 59-69%. | 36 |
| Yuvaci 2020 (373) | Cross-sectional study | Turkey | Women, 18-40 years old, married, with a planned/desired live single pregnancy, BMI 20-30, parity ≤2, a minimum of 8 years of education, medium-high income levels, non-smoking, Turkish-speaking, taking vitamin D at the level recommended by the Ministry of Health, no psychiatric disorders, no chronic diseases, no drugs, no alcohol use. | Infants, 4-6 weeks old, exclusively breastfed. | Collection of mature milk 4-6 weeks pp. Samples were centrifuged and serum was stored frozen until analysis. | 25OHD3 was measured in centrifuged milk serum with IDS-iSYS Multi-Discipline Autmated System (serial number B0509, made in France), using automated chemiluminescence immunoassay (CLIA), intended for serum, plasma, urine or saliva. The range of quantification was 7-125 ng/mL and values <7 ng/mL were considered non-detectable. | 75 |

Note : Studies in blue, indicate where there were samples with non-detectable levels.

## References

1. Abdel-Kader MM, Abdel-Hay A, el-Safouri S, Saad el-Din J, Abdel-Aziz MT, Kamal I, et al. Clinical, biochemical, and experimental studies on lactation. 3. Biochemical changes induced in human milk by gestagens. Am J Obstet Gynecol. 1969 Nov 15;105(6):978–85.

2. Adesiyan AA, Akiibinu MO, Olisekodia MJ, Onuegbu AJ, Adeyeye AD. Concentrations of Some Biochemical Parameters in Breast Milk of a Population of Nigerian Nursing Mothers Using Hormonal Contraceptives. Pakistan Journal of Nutrition. 2011 Feb 15;10(3):249–53.

3. Allen JC, Keller RP, Archer P, Neville MC. Studies in human lactation: milk composition and daily secretion rates of macronutrients in the first year of lactation. The American Journal of Clinical Nutrition. 1991 Jul 1;54(1):69–80.

4. Neville MC, Keller R, Seacat J, Lutes V, Neifert M, Casey C, et al. Studies in human lactation: milk volumes in lactating women during the onset of lactation and full lactation. Am J Clin Nutr. 1988 Dec;48(6):1375–86.

5. Anastácio A da S, da Silveira CLP, Miekeley N, Donangelo CM. Distribution of lead in human milk fractions: relationship with essential minerals and maternal blood lead. Biol Trace Elem Res. 2004;102(1–3):27–37.

6. Anderson RR. Variations in major minerals of human milk during the first 5 months of lactation. Nutrition Research. 1992 Jun;12(6):701–11.

7. Arias‐Borrego A, Soto Cruz FJ, Selma‐Royo M, Bäuerl C, García Verdevio E, Pérez‐Cano FJ, et al. Metallomic and Untargeted Metabolomic Signatures of Human Milk from SARS‐CoV‐2 Positive Mothers. Molecular Nutrition Food Res. 2022 Aug;66(16):2200071.

8. Carneiro T, de Oliveira JE. Nutritional studies in human lactation in Brazil. 1. Chemical composition of breast milk. J Trop Pediatr Environ Child Health. 1973 Dec;19(4):384–7.

9. Arver S, Bucht E, Sjöberg HE. Calcitonin-like immunoreactivity in human milk, longitudinal alterations and divalent cations. Acta Physiol Scand. 1984 Dec;122(4):461–4.

10. Astolfi ML, Marconi E, Protano C, Vitali M, Schiavi E, Mastromarino P, et al. Optimization and validation of a fast digestion method for the determination of major and trace elements in breast milk by ICP-MS. Anal Chim Acta. 2018 Dec 21;1040:49–62.

11. Atkinson SA, Radde IC, Chance GW, Bryan MH, Anderson GH. Macro-mineral content of milk obtained during early lactation from mothers of premature infants. Early Hum Dev. 1980 Mar;4(1):5–14.

12. Atkinson SA, Radde IC, Anderson GH. Macromineral balances in premature infants fed their own mothers’ milk or formula. J Pediatr. 1983 Jan;102(1):99–106.

13. Barakat MZ, Shehab SK, Naguib N. A study of the calcium-phosphorus ratio of different milks. Zentralbl Veterinarmed A. 1969 Jul;16(5):444–9.

14. Barltrop D, Hillier R. Calcium and phosphorus content of transitional and mature human milk. Acta Paediatr Scand. 1974 May;63(3):347–50.

15. Bauer J, Gerss J. Longitudinal analysis of macronutrients and minerals in human milk produced by mothers of preterm infants. Clin Nutr. 2011 Apr;30(2):215–20.

16. Bezerra FF, Cabello GMK, Mendonça LMC, Donangelo CM. Bone mass and breast milk calcium concentration are associated with vitamin D receptor gene polymorphisms in adolescent mothers. J Nutr. 2008 Feb;138(2):277–81.

17. Bilston-John SH, Narayanan A, Tat Lai C, Rea A, Joseph J, Geddes DT. Daily and within-feed variation of macro- and trace-element concentrations in human milk and implications for sampling. Food Chemistry. 2021 Nov;363:130179.

18. Björklund KL, Vahter M, Palm B, Grandér M, Lignell S, Berglund M. Metals and trace element concentrations in breast milk of first time healthy mothers: a biological monitoring study. Environ Health. 2012 Dec 14;11:92.

19. Bloom H, Lewis IC, Noller BN. Elemental analysis of dried milks used in infant feeding. Aust Paediatr J. 1978 Dec;14(4):259–64.

20. Bocca B, Alimonti A, Giglio L, Di Pasquale M, Caroli S, Ambruzzi MA, et al. Nutritive significance of element speciation in breast milk. The case of calcium, copper, iron, magnesium, manganese, and zinc. Adv Exp Med Biol. 2000;478:385–6.

21. Bolognini Pereira K, de Azeredo VB, Barros da Sileira C, Pedruzzi LM. Composition of breast milk of lactating adolescents in function of time of lactation. Nutr Hosp. 2013 Nov 1;28(6):1971–6.

22. Bosscher D, Van Caillie-Bertrand M, Robberecht H, Van Dyck K, Van Cauwenbergh R, Deelstra H. In vitro availability of calcium, iron, and zinc from first-age infant formulae and human milk. J Pediatr Gastroenterol Nutr. 2001 Jan;32(1):54–8.

23. Bosscher D, Van Caillie-Bertrand M, Van Cauwenbergh R, Deelstra H. Availabilities of calcium, iron, and zinc from dairy infant formulas is affected by soluble dietary fibers and modified starch fractions. Nutrition. 2003 Jul;19(7–8):641–5.

24. Braga LPM, Palhares DB. Effect of evaporation and pasteurization in the biochemical and immunological composition of human milk. J Pediatr (Rio J). 2007 Feb;83(1):59–63.

25. Butte NF, Garza C, Johnson CA, Smith EO, Nichols BL. Longitudinal changes in milk composition of mothers delivering preterm and term infants. Early Hum Dev. 1984 Feb;9(2):153–62.

26. Butte NF, Garza C, Smith EO, Wills C, Nichols BL. Macro- and trace-mineral intakes of exclusively breast-fed infants. Am J Clin Nutr. 1987 Jan;45(1):42–8.

27. Butte NF, Garza C, Smith EO, Nichols BL. Human milk intake and growth in exclusively breast-fed infants. J Pediatr. 1984 Feb;104(2):187–95.

28. Butte NF, Garza C, Stuff JE, Smith EO, Nichols BL. Effect of maternal diet and body composition on lactational performance. Am J Clin Nutr. 1984 Feb;39(2):296–306.

29. Butte NF, Wills C, Jean CA, Smith EO, Garza C. Feeding patterns of exclusively breast-fed infants during the first four months of life. Early Hum Dev. 1985 Dec;12(3):291–300.

30. Butte NF, Garza C, Burr R, Goldman AS, Kennedy K, Kitzmiller JL. Milk composition of insulin-dependent diabetic women. J Pediatr Gastroenterol Nutr. 1987 Dec;6(6):936–41.

31. Butts CA, Hedderley DI, Herath TD, Paturi G, Glyn-Jones S, Wiens F, et al. Human Milk Composition and Dietary Intakes of Breastfeeding Women of Different Ethnicity from the Manawatu-Wanganui Region of New Zealand. Nutrients. 2018 Sep 4;10(9).

32. Bzikowska-Jura A, Wesołowska A, Sobieraj P, Michalska-Kacymirow M, Bulska E, Starcevic I. Maternal diet during breastfeeding in correlation to calcium and phosphorus concentrations in human milk. J Hum Nutr Diet [Internet]. 2022;((Bzikowska-Jura A.; Wesołowska A.; Starcevic I.) Department of Medical Biology, Faculty of Health Sciences, Medical University of Warsaw, Laboratory of Human Milk and Lactation Research at Regional Human Milk Bank in Holy Family Hospital, Warsaw, Poland). Available from: https://www.embase.com/search/results?subaction=viewrecord&id=L639253392&from=export

33. Campbell-Yeo ML, Allen AC, Joseph KS, Ledwidge JM, Caddell K, Allen VM, et al. Effect of domperidone on the composition of preterm human breast milk. Pediatrics. 2010 Jan;125(1):e107-114.

34. Cancela L, Le Boulch N, Miravet L. Relationship between the vitamin D content of maternal milk and the vitamin D status of nursing women and breast-fed infants. J Endocrinol. 1986 Jul;110(1):43–50.

35. Capriati T, Goffredo BM, Argentieri M, De Vivo L, Bernaschi P, Cairoli S, et al. A Modified Holder Pasteurization Method for Donor Human Milk: Preliminary Data. Nutrients. 2019 May 22;11(5).

36. Carias D, Velásquez G, Cioccia AM, Piñero D, Inciarte H, Hevia P. [The effect of lactation time on the macronutrient and mineral composition of milk from Venezuelan women]. Arch Latinoam Nutr. 1997 Jun;47(2):110–7.

37. Castro F, Harari F, Llanos M, Vahter M, Ronco A. Maternal–Child Transfer of Essential and Toxic Elements through Breast Milk in a Mine-Waste Polluted Area. Amer J Perinatol. 2014 Mar 28;31(11):993–1002.

38. Chan GM. Human milk calcium and phosphate levels of mothers delivering term and preterm infants. J Pediatr Gastroenterol Nutr. 1982;1(2):201–5.

39. Chen HH, Jiang HY, Yang WQ. Investigation and analysis of diet and nutrients in breast milk of mothers from Sanjiang Dong and Nanning. J Guangxi Med Univ. 2007;24:644–7.

40. Citrakesumasari, Kalsum V, Majiding CM, Rahman TAA, Kurniati Y. Mineral Concentrations in Breast Milk across Infant Birth Weight. Pakistan Journal of Nutrition. 2019 Dec 15;19(1):32–7.

41. Codo CRB, Caldas JP de S, Peixoto RRA, Sanches VL, Guiraldelo TC, Cadore S, et al. Electrolyte and mineral composition of term donor human milk before and after pasteurization and of raw milk of preterm mothers. Rev Paul Pediatr. 2018 Jun;36(2):141–7.

42. Dagnelie PC, van Staveren WA, Roos AH, Tuinstra LG, Burema J. Nutrients and contaminants in human milk from mothers on macrobiotic and omnivorous diets. Eur J Clin Nutr. 1992 May;46(5):355–66.

43. Morón de Salim A, Cruces ME, Oviedo Colón G. Calcio y magnesio en leche madura de mujeres lactantes de una comunidad de Valencia. Venezuela. Revista de la Facultad de Ciencias de la Salud. 2010;14(2):8–13.

44. Deng B, Zhang H, Yan C, Zhang L. [Levels of mineral elements composition and heavy metal pollution in human breast milk in Shenzhen City]. Wei Sheng Yan Jiu. 2009 May;38(3):293–5.

45. DeSantiago S, Alonso L, Halhali A, Larrea F, Isoard F, Bourges H. Negative calcium balance during lactation in rural Mexican women. Am J Clin Nutr. 2002 Oct;76(4):845–51.

46. Dewey KG, Lönnerdal B. Milk and nutrient intake of breast-fed infants from 1 to 6 months: relation to growth and fatness. J Pediatr Gastroenterol Nutr. 1983;2(3):497–506.

47. Dewey KG, Finley DA, Lönnerdal B. Breast milk volume and composition during late lactation (7-20 months). J Pediatr Gastroenterol Nutr. 1984 Nov;3(5):713–20.

48. Dorea JG, Myazaki E. Calcium and phosphorus in milk of Brazilian mothers using oral contraceptives. J Am Coll Nutr. 1998 Dec;17(6):642–6.

49. Dumrongwongsiri O, Chongviriyaphan N, Chatvutinun S, Phoonlabdacha P, Sangcakul A, Siripinyanond A, et al. Dietary Intake and Milk Micronutrient Levels in Lactating Women with Full and Partial Breastfeeding. Matern Child Health J. 2021 Jun;25(6):991–7.

50. Dutta S, Saini S, Prasad R. Changes in preterm human milk composition with particular reference to introduction of mixed feeding. Indian Pediatr. 2014 Dec;51(12):997–9.

51. El-Farrash RA, Ismail EAR, Nada AS. Cord blood iron profile and breast milk micronutrients in maternal iron deficiency anemia. Pediatr Blood Cancer. 2012 Feb;58(2):233–8.

52. Feeley RM, Eitenmiller RR, Jones JB, Barnhart H. Calcium, phosphorus, and magnesium contents of human milk during early lactation. J Pediatr Gastroenterol Nutr. 1983 May;2(2):262–7.

53. Fly AD, Uhlin KL, Wallace JP. Major mineral concentrations in human milk do not change after maximal exercise testing. Am J Clin Nutr. 1998 Aug;68(2):345–9.

54. Fransson GB, Lönnerdal B. Zinc, copper, calcium, and magnesium in human milk. J Pediatr. 1982 Oct;101(4):504–8.

55. Fransson GB, Lönnerdal B. Distribution of trace elements and minerals in human and cow’s milk. Pediatr Res. 1983 Nov;17(11):912–5.

56. Fransson GB, Gebre-Medhin M, Hambraeus L. The human milk contents of iron, copper, zinc, calcium and magnesium in a population with a habitually high intake of iron. Acta Paediatr Scand. 1984 Jul;73(4):471–6.

57. Friel JK, Andrews WL, Jackson SE, Longerich HP, Mercer C, McDonald A, et al. Elemental composition of human milk from mothers of premature and full-term infants during the first 3 months of lactation. Biol Trace Elem Res. 1999 Mar;67(3):225–47.

58. Garg M, Thirupuram S, Saha K. Colostrum composition, maternal diet and nutrition in north India. J Trop Pediatr. 1988 Apr;34(2):79–87.

59. Garza C, Johnson CA, Smith EO, Nichols BL. Changes in the nutrient composition of human milk during gradual weaning. Am J Clin Nutr. 1983 Jan;37(1):61–5.

60. Gates A, Marin T, De Leo G, Waller JL, Stansfield BK. Nutrient composition of preterm mother’s milk and factors that influence nutrient content. Am J Clin Nutr. 2021 Nov 8;114(5):1719–28.

61. Gibson RS, Rahmannia S, Diana A, Leong C, Haszard JJ, Hampel D, et al. Association of maternal diet, micronutrient status, and milk volume with milk micronutrient concentrations in Indonesian mothers at 2 and 5 months postpartum. Am J Clin Nutr. 2020 Oct 1;112(4):1039–50.

62. Daniels L, Gibson RS, Diana A, Haszard JJ, Rahmannia S, Luftimas DE, et al. Micronutrient intakes of lactating mothers and their association with breast milk concentrations and micronutrient adequacy of exclusively breastfed Indonesian infants. The American Journal of Clinical Nutrition. 2019 Aug 1;110(2):391–400.

63. Diana A, Haszard JJ, Houghton LA, Gibson RS. Breastmilk intake among exclusively breastfed Indonesian infants is negatively associated with maternal fat mass. European Journal of Clinical Nutrition. 2019 Aug;73(8):1206–8.

64. Góes HCA, Torres AG, Donangelo CM, Trugo NMF. Nutrient composition of banked human milk in Brazil and influence of processing on zinc distribution in milk fractions. Nutrition. 2002 Aug;18(7–8):590–4.

65. Greer FR, Tsang RC, Levin RS, Searcy JE, Wu R, Steichen JJ. Increasing serum calcium and magnesium concentrations in breast-fed infants: Longitudinal studies of minerals in human milk and in sera of nursing mothers and their infants. J Pediatr. 1982 Jan;100(1):59–64.

66. Greer FR, McCormick A. Improved bone mineralization and growth in premature infants fed fortified own mother’s milk. J Pediatr. 1988 Jun;112(6):961–9.

67. Gross SJ, David RJ, Bauman L, Tomarelli RM. Nutritional composition of milk produced by mothers delivering preterm. J Pediatr. 1980 Apr;96(4):641–4.

68. Gulson BL, Mizon KJ, Korsch MJ, Mahaffey KR, Taylor AJ. Dietary intakes of selected elements from longitudinal 6-day duplicate diets for pregnant and nonpregnant subjects and elemental concentrations of breast milk and infant formula. Environ Res. 2001 Dec;87(3):160–74.

69. Gupta AP, Bhandari B, Gupta A, Goyal S. Mineral content of breast milk from north Indian mothers giving birth preterm and at term--implication for mineral nutrition of preterm infants. J Trop Pediatr. 1984 Oct;30(5):286–8.

70. Gutikova LV. [Chemical composition of milk of puerperas suffered from gestosis of different degree of severity]. Biomed Khim. 2007 Jun;53(3):332–7.

71. Harzer G, Haug M, Bindels JG. Biochemistry of maternal milk in early lactation. Hum Nutr Appl Nutr. 1986;40 Suppl 1:11–8.

72. Hibberd CM, Brooke OG, Carter ND, Haug M, Harzer G. Variation in the composition of breast milk during the first 5 weeks of lactation: implications for the feeding of preterm infants. Arch Dis Child. 1982 Sep;57(9):658–62.

73. Honda R. Cadmium exposure and trace elements in human breast milk. Toxicology. 2003 Apr 22;186(3):255–9.

74. Hou YM, Yu S, Zheng XX. [Breast milk composition of lactating women from Jinan]. Matern Child Health Care Cn. 2008;23:241–3.

75. Hsu YC, Chen CH, Lin MC, Tsai CR, Liang JT, Wang TM. Changes in preterm breast milk nutrient content in the first month. Pediatr Neonatol. 2014 Dec;55(6):449–54.

76. Huang Z. Study on the diet of 269 nursing mothers and mineral contents in their breast milk [Master thesis]. Zhongnan University; 2014.

77. Hunt CD, Butte NF, Johnson LK. Boron concentrations in milk from mothers of exclusively breast-fed healthy full-term infants are stable during the first four months of lactation. J Nutr. 2005 Oct;135(10):2383–6.

78. Itabashi K, Miura A, Okuyama K, Takeuchi T, Kitazawa S. Estimated nutritional intake based on the reference growth curves for extremely low birthweight infants. Pediatr Int. 1999 Feb;41(1):70–7.

79. Itriago A, Carrión N, Fernández A, Puig M, Dini E. [Zinc, copper, iron, calcium, phosphorus and magnesium content of maternal milk during the first 3 weeks of lactation]. Arch Latinoam Nutr. 1997 Mar;47(1):14–22.

80. Iwai K, Iwai-Shimada M, Asato K, Nakai K, Kobayashi Y, Nakayama SF, et al. Intra- and Inter-Day Element Variability in Human Breast Milk: Pilot Study. Toxics. 2022 Feb 25;10(3):109.

81. Jarjou LMA, Prentice A, Sawo Y, Laskey MA, Bennett J, Goldberg GR, et al. Randomized, placebo-controlled, calcium supplementation study in pregnant Gambian women: effects on breast-milk calcium concentrations and infant birth weight, growth, and bone mineral accretion in the first year of life. Am J Clin Nutr. 2006 Mar;83(3):657–66.

82. Jarjou LMA, Prentice A, Bennett J. Impact of Calcium Supplementation in the Preceding Pregnancy on the Human Milk Calcium Concentration of Gambian Women. In: Pickering LK, Morrow AL, Ruiz-Palacios GM, Schanler RJ, editors. Protecting Infants through Human Milk [Internet]. Boston, MA: Springer US; 2004 [cited 2021 Jan 17]. p. 347–9. (Advances in Experimental Medicine and Biology; vol. 554). Available from: http://link.springer.com/10.1007/978-1-4757-4242-8_38

83. Karbasi S, Bahrami A, Asadi Z, Shahbeiki F, Naseri M, Zarban A, et al. The association of maternal dietary quality and the antioxidant-proxidant balance of human milk. Int Breastfeed J. 2022 Aug 8;17(1):56.

84. Karra MV, Udipi SA, Kirksey A, Roepke JL. Changes in specific nutrients in breast milk during extended lactation. Am J Clin Nutr. 1986 Apr;43(4):495–503.

85. Karra MV, Kirksey A, Galal O, Bassily NS, Harrison GG, Jerome NW. Zinc, calcium, and magnesium concentrations in milk from American and Egyptian women throughout the first 6 months of lactation. Am J Clin Nutr. 1988 Apr;47(4):642–8.

86. Kent JC, Arthur PG, Retallack RW, Hartmann PE. Calcium, phosphate and citrate in human milk at initiation of lactation. J Dairy Res. 1992 May;59(2):161–7.

87. Khatir Sam A, Osman MM, El-Khangi FA. Determination of protein and trace elementsin human milk using NAA and XFR techniques. Journal of Radioanalytical and Nuclear Chemistry. 1998;231(1–2):21–3.

88. Kim H, Jung BM, Lee BN, Kim YJ, Jung JA, Chang N. Retinol, α-tocopherol, and selected minerals in breast milk of lactating women with full-term infants in South Korea. Nutr Res Pract. 2017 Feb;11(1):64–9.

89. Kippler M, Lönnerdal B, Goessler W, Ekström EC, Arifeen SE, Vahter M. Cadmium interacts with the transport of essential micronutrients in the mammary gland - a study in rural Bangladeshi women. Toxicology. 2009 Mar 4;257(1–2):64–9.

90. Kippler M, Hossain MB, Lindh C, Moore SE, Kabir I, Vahter M, et al. Early life low-level cadmium exposure is positively associated with increased oxidative stress. Environ Res. 2012 Jan;112:164–70.

91. Kirksey A, Ernst JA, Roepke JL, Tsai TL. Influence of mineral intake and use of oral contraceptives before pregnancy on the mineral content of human colostrum and of more mature milk. Am J Clin Nutr. 1979 Jan;32(1):30–9.

92. Klein LD, Breakey AA, Scelza B, Valeggia C, Jasienska G, Hinde K. Concentrations of trace elements in human milk: Comparisons among women in Argentina, Namibia, Poland, and the United States. PLoS One. 2017;12(8):e0183367.

93. Kulski JK, Hartmann PE. Changes in human milk composition during the initiation of lactation. Aust J Exp Biol Med Sci. 1981 Feb;59(1):101–14.

94. Laskey MA, Prentice A, Shaw J, Zachou T, Ceesay SM, Vasquez-Velasquez L, et al. Breast-milk calcium concentrations during prolonged lactation in British and rural Gambian mothers. Acta Paediatr Scand. 1990 May;79(5):507–12.

95. Laskey MA, Dibba B, Prentice A. Low Ratios of Calcium to Phosphorus in the Breast-Milk of Rural Gambian Mothers. Acta Paediatrica. 1991 Feb;80(2):250–1.

96. Laskey MA, Prentice A, Hanratty LA, Jarjou LM, Dibba B, Beavan SR, et al. Bone changes after 3 mo of lactation: influence of calcium intake, breast-milk output, and vitamin D-receptor genotype. Am J Clin Nutr. 1998 Apr;67(4):685–92.

97. Lemons JA, Moye L, Hall D, Simmons M. Differences in the composition of preterm and term human milk during early lactation. Pediatr Res. 1982 Feb;16(2):113–7.

98. Levi M, Hjelm C, Harari F, Vahter M. ICP-MS measurement of toxic and essential elements in human breast milk. A comparison of alkali dilution and acid digestion sample preparation methods. Clin Biochem. 2018 Mar;53:81–7.

99. Li JZ, Yoshinaga J, Suzuki T, Abe M, Morita M. Mineral and trace element content of human transitory milk indentified with inductively coupled plasma atomic emission spectrometry. J Nutr Sci Vitaminol (Tokyo). 1990 Feb;36(1):65–74.

100. Li M, Liu Y, Gao G, Liu XH, Huang CY. [Calcium, iron and magnesium levels in pretem infants and their mothers]. Chinese Journal of Contemporary Pediatrics. 2008 Jun;10(3):349–52.

101. Li C, Solomons NW, Scott ME, Koski KG. Minerals and Trace Elements in Human Breast Milk Are Associated with Guatemalan Infant Anthropometric Outcomes within the First 6 Months. J Nutr. 2016 Oct;146(10):2067–74.

102. Li C, Solomons NW, Scott ME, Koski KG. Subclinical mastitis (SCM) and proinflammatory cytokines are associated with mineral and trace element concentrations in human breast milk. J Trace Elem Med Biol. 2018 Mar;46:55–61.

103. Li C, Solomons NW, Scott ME, Koski KG. Anthropometry before Day 46 and Growth Velocity before 6 Months of Guatemalan Breastfed Infants Are Associated with Subclinical Mastitis and Milk Cytokines, Minerals, and Trace Elements. J Nutr. 2019 Sep 1;149(9):1651–9.

104. Lin TH, Jong YJ, Chiang CH, Yang MH. Longitudinal changes in Ca, Mg, Fe, Cu, and Zn in breast milk of women in Taiwan over a lactation period of one year. Biol Trace Elem Res. 1998 May;62(1–2):31–41.

105. Lipsman S, Dewey KG, Lönnerdal B. Breast-feeding among teenage mothers: milk composition, infant growth, and maternal dietary intake. J Pediatr Gastroenterol Nutr. 1985 Jun;4(3):426–34.

106. Liu Q, Xue H, Guan K. [Determination of zinc, copper, iron, calcium and magnesium in breast milk and its clinical significance]. Chin J Obstetr Gynec and Pediatr. 2008;4:537–9.

107. Liu AP, Zhang C, Zhao CS. [Comparative analysis of some elements in breast milk of lactating women from Lanzhou]. Gansu Sci Technol. 2014;30(4):124–5.

108. Luo Y, Zhang B, Chen M, Wang J, Zhang X, Gao WY, et al. Rapid and simultaneous determination of essential minerals and trace elements in human milk by improved flame atomic absorption spectroscopy (FAAS) with microwave digestion. J Agric Food Chem. 2010 Sep 8;58(17):9396–400.

109. Mahdavi R, Taghipour S, Ostadrahimi A, Nikniaz L, Hezaveh SJG. A pilot study of synbiotic supplementation on breast milk mineral concentrations and growth of exclusively breast fed infants. J Trace Elem Med Biol. 2015 Apr;30:25–9.

110. Mandiá N, Bermejo-Barrera P, Herbello P, López-Suárez O, Fraga JM, Fernández-Pérez C, et al. Human Milk Concentrations of Minerals, Essential and Toxic Trace Elements and Association with Selective Medical, Social, Demographic and Environmental Factors. Nutrients. 2021 May 31;13(6):1885.

111. Maru M, Birhanu T, Tessema DA. Calcium, magnesium, iron, zinc and copper, compositions of human milk from populations with cereal and “enset” based diets. Ethiop J Health Sci. 2013 Jul;23(2):90–7.

112. Mastroeni SSBS, Okada IA, Rondó PHC, Duran MC, Paiva AA, Neto JM. Concentrations of Fe, K, Na, Ca, P, Zn and Mg in maternal colostrum and mature milk. J Trop Pediatr. 2006 Aug;52(4):272–5.

113. Mataloun MM, Leone CR. Human milk mineral intake and serum concentrations of calcium and phosphorus in newborn term infants: influence of intrauterine growth restriction. Acta Paediatr. 2000 Sep;89(9):1093–7.

114. Minato T, Nomura K, Asakura H, Aihara A, Hiraike H, Hino Y, et al. Maternal Undernutrition and Breast Milk Macronutrient Content Are Not Associated with Weight in Breastfed Infants at 1 and 3 Months after Delivery. Int J Environ Res Public Health. 2019 Sep 9;16(18).

115. Motil KJ, Kertz B, Thotathuchery M. Lactational performance of adolescent mothers shows preliminary differences from that of adult women. J Adolesc Health. 1997 Jun;20(6):442–9.

116. Nagra SA. Longitudinal study in biochemical composition of human milk during first year of lactation. J Trop Pediatr. 1989 Jun;35(3):126–8.

117. Neville MC, Keller RP, Seacat J, Casey CE, Allen JC, Archer P. Studies on human lactation. I. Within-feed and between-breast variation in selected components of human milk. Am J Clin Nutr. 1984 Sep;40(3):635–46.

118. Nickkho-Amiry M, Prentice A, Ledi F, Laskey MA, Das G, Berry JL, et al. Maternal vitamin D status and breast milk concentrations of calcium and phosphorus. Arch Dis Child. 2008 Feb;93(2):179.

119. Noh S, Lee E. Relationship between Selected Trace Elements in Human Milk and Psychosocial Characteristics in Korean Early Postpartum Women. IJERPH. 2021 Jan 5;18(1):350.

120. Oliveira MM, Aragon DC, Bomfim VS, Trevilato TMB, Alves LG, Heck AR, et al. Development of a human milk concentrate with human milk lyophilizate for feeding very low birth weight preterm infants: A preclinical experimental study. PLoS One. 2019;14(2):e0210999.

121. Ortega RM, Martínez RM, Quintas ME, López-Sobaler AM, Andrés P. Calcium levels in maternal milk: relationships with calcium intake during the third trimester of pregnancy. Br J Nutr. 1998 Jun;79(6):501–7.

122. Parr RM, DeMaeyer EM, Iyengar VG, Byrne AR, Kirkbright GF, Schöch G, et al. Minor and trace elements in human milk from Guatemala, Hungary, Nigeria, Philippines, Sweden, and Zaire. Results from a WHO/IAEA joint project. Biol Trace Elem Res. 1991 Apr;29(1):51–75.

123. Perrin MT, Fogleman AD, Newburg DS, Allen JC. A longitudinal study of human milk composition in the second year postpartum: implications for human milk banking. Matern Child Nutr. 2017 Jan;13(1).

124. Picciano MF, Calkins EJ, Garrick JR, Deering RH. Milk and mineral intakes of breastfed infants. Acta Paediatr Scand. 1981 Mar;70(2):189–94.

125. Pietrzak-Fiećko R, Kamelska-Sadowska AM. The Comparison of Nutritional Value of Human Milk with Other Mammals’ Milk. Nutrients. 2020 May 14;12(5).

126. Prentice A, Jarjou LM, Cole TJ, Stirling DM, Dibba B, Fairweather-Tait S. Calcium requirements of lactating Gambian mothers: effects of a calcium supplement on breast-milk calcium concentration, maternal bone mineral content, and urinary calcium excretion. Am J Clin Nutr. 1995 Jul;62(1):58–67.

127. Prentice A, Yan L, Jarjou LM, Dibba B, Laskey MA, Stirling DM, et al. Vitamin D status does not influence the breast-milk calcium concentration of lactating mothers accustomed to a low calcium intake. Acta Paediatr. 1997 Sep;86(9):1006–8.

128. Prentice A. Biochemical Markers of Calcium and Bone Metabolism during 18 Months of Lactation in Gambian Women Accustomed to a Low Calcium Intake and in Those Consuming a Calcium Supplement. Journal of Clinical Endocrinology & Metabolism. 1998 Apr 1;83(4):1059–66.

129. Qian J, Wu S, Zhang W, Cao LJ, Yang HW, Ao LM. An investigation of nutrients of human milk in Shanghai area. Shanghai Med J. 2002;25(7):396–8.

130. Qian J, Chen T, Lu W, Wu S, Zhu J. Breast milk macro- and micronutrient composition in lactating mothers from suburban and urban Shanghai. J Paediatr Child Health. 2010 Mar;46(3):115–20.

131. Qian C li, Tian F, Chen R di, Li N, Mao YY, Lu P ting, et al. [The investigation of minerals in human breast milk across lactation stages in six representative areas of China]. Chinese Journal of Disease Control & Prevention. 2022;26(9):1037–42.

132. Bortolozo EAFQ, Tiboni EB, Cândido LMB. [Milk from human milk banks for low birthweight newborns: nutritional contents and supplementation]. Rev Panam Salud Publica. 2004 Sep;16(3):199–205.

133. Rana IA, Gilani M, Jafri S. Nutritive value of human milk and nutritional status of wives of army personnel of low income from Rawalpindi. J Pak Med Assoc. 1990 May;40(5):109–12.

134. Rodríguez Rodríguez EM, Sanz Alaejos M, Díaz Romero C. [Concentrations of calcium, magnesium, sodium and potassium in human milk and infant formulas]. Arch Latinoam Nutr. 2002 Dec;52(4):406–12.

135. Rona MSS, Novak FR, Portilho M, Pelissari FM, Martins ABT, Matioli G. Efeito do tempo e da temperatura de estocagem nas determinações de acidez, cálcio, proteínas e lipídeos de leite de doadoras de bancos de leite humano. Revista Brasileira de Saúde Materno Infantil. 2008 Sep;8(3):257–63.

136. Sabatier M, Garcia-Rodenas CL, Castro CAD, Kastenmayer P, Vigo M, Dubascoux S, et al. Longitudinal Changes of Mineral Concentrations in Preterm and Term Human Milk from Lactating Swiss Women. Nutrients. 2019 Aug 9;11(8).

137. Salah ET, Malik NME, Hassan MS, Mohammed IS, Mohamed M, Mohamed MO, et al. How does the fasting of Ramadan affect breast milk constituents? Sudan JMS. 2016;11(1):17–22.

138. Samuel TM, De Castro CA, Dubascoux S, Affolter M, Giuffrida F, Billeaud C, et al. Subclinical Mastitis in a European Multicenter Cohort: Prevalence, Impact on Human Milk (HM) Composition, and Association with Infant HM Intake and Growth. Nutrients. 2019 Dec 30;12(1).

139. Sann L, Bienvenu F, Lahet C, Bienvenu J, Bethenod M. Comparison of the composition of breast milk from mothers of term and preterm infants. Acta Paediatr Scand. 1981 Jan;70(1):115–6.

140. Schanler RJ, Oh W. Composition of breast milk obtained from mothers of premature infants as compared to breast milk obtained from donors. J Pediatr. 1980 Apr;96(4):679–81.

141. Seki K, Kato T, Sekiya S, Makimura N, Kudoh K, Furuya K, et al. Parathyroid-hormone-related protein in human milk and its relation to milk calcium. Gynecol Obstet Invest. 1997;44(2):102–6.

142. Shehadeh N, Aslih N, Shihab S, Werman MJ, Sheinman R, Shamir R. Human milk beyond one year post-partum: lower content of protein, calcium, and saturated very long-chain fatty acids. J Pediatr. 2006 Jan;148(1):122–4.

143. Shi Y dong, Sun G qing, Zhang Z guo, Deng X, Kang X hong, Liu Z dong, et al. The chemical composition of human milk from Inner Mongolia of China. Food Chemistry. 2011 Aug;127(3):1193–8.

144. Silva PR, Dorea JG, Boaventura GR. Multielement determination in small samples of human milk by inductively coupled plasma atomic emission spectrometry. Biol Trace Elem Res. 1997;59(1–3):57–62.

145. Sunarić S, Denić M, Lalić J, Jovanović T, Spasić A, Živković J, et al. Physicochemical and biochemical parameters in milk of Serbian breastfeeding women. Turk J Med Sci. 2017 Feb 27;47(1):246–51.

146. Szukalska M, Merritt TA, Lorenc W, Sroczyńska K, Miechowicz I, Komorowicz I, et al. Toxic metals in human milk in relation to tobacco smoke exposure. Environ Res. 2021 Jun;197:111090.

147. Taravati Javad M, Vahidinia A, Samiee F, Elaridi J, Leili M, Faradmal J, et al. Analysis of aluminum, minerals and trace elements in the milk samples from lactating mothers in Hamadan, Iran. J Trace Elem Med Biol. 2018 Dec;50:8–15.

148. Terheggen HG. [On the calcium, potassium and soidum content of human milk. A flame analysis]. Z Kinderheilkd. 1965 Mar 10;92:193–200.

149. Thacher TD, Pettifor JM, Fischer PR, Okolo SN, Prentice A. Case-control study of breast milk calcium in mothers of children with and without nutritional rickets. Acta Paediatr. 2006 Jul;95(7):826–32.

150. Toddywalla WS, Joshi L, Virkar K. Effect of contraceptive steroids on human lactation. American Journal of Obstetrics and Gynecology. 1977 Feb;127(3):245–9.

151. Umeta M, West CE, Verhoef H, Haidar J, Hautvast JGAJ. Factors associated with stunting in infants aged 5-11 months in the Dodota-Sire District, rural Ethiopia. J Nutr. 2003 Apr;133(4):1064–9.

152. Van Steenbergen WM, Kusin JA, Van Rens MM. Lactation Performance of Akamba Mothers, Kenya. Breast Feeding Behaviour, Breast Milk Yield and Composition. Journal of Tropical Pediatrics. 1981 Jun 1;27(3):155–61.

153. Vanderja GT DJ, Shores JT, Okolo SN, Millson M, Ezeogu AF, Glew RH. Mineral content of the milk of Fulani women and the sera of their breast-fed infants. Highland Medical Research Journal. 2002;1(2):6–11.

154. Shores JT, VanderJagt DJ, Millson M, Huang YS, Glew RH. Correlation between the content of intermediate chain-length fatty acids and copper in the milk of Fulani women. Prostaglandins, Leukotrienes and Essential Fatty Acids (PLEFA). 2000 Oct;63(4):203–7.

155. Vaughan LA, Weber CW, Kemberling SR. Longitudinal changes in the mineral content of human milk. Am J Clin Nutr. 1979 Nov;32(11):2301–6.

156. Vítolo MR, Valente Soares LM, Carvalho EB, Cardoso CB. Calcium and magnesium concentrations in mature human milk: influence of calcium intake, age and socioeconomic level. Arch Latinoam Nutr. 2004 Mar;54(1):118–22.

157. Wang XS, Wang CG. [Calcium, phosphorus, selenium composition in breast milk and GPX activity comparison]. Cn Public Health. 2002;18:991.

158. Wang WQ, Liu LM, Sun XM, Zhang ZF, Zhang JH, Jia M, et al. [Changes of inorganic elements of breast milk in rural lactating women]. Chinese Journal of Clinical Nutrition. 2007 Apr 1;15:82–5.

159. Wei M, Deng Z, Liu B, Ye W, Fan Y, Liu R, et al. Investigation of amino acids and minerals in Chinese breast milk. J Sci Food Agric. 2020 Aug;100(10):3920–31.

160. Yamawaki N, Yamada M, Kan-no T, Kojima T, Kaneko T, Yonekubo A. Macronutrient, mineral and trace element composition of breast milk from Japanese women. J Trace Elem Med Biol. 2005;19(2–3):171–81.

161. Yoneyama K, Ikeda J, Nagata H. [Interrelations of the calcium concentration in breast milk with maternal intake of cow’s milk and milk products, bone resorption and bone mineral density during lactation]. Nihon Eiseigaku Zasshi. 1997 Jan;51(4):770–9.

162. Yoshinaga J, Li JZ, Suzuki T, Karita K, Abe M, Fujii H, et al. Trace elements in human transitory milk. Variation caused by biological attributes of mother and infant. Biol Trace Elem Res. 1991 Nov;31(2):159–70.

163. Zhao XH, Zhao A, Zhang YM. [The correlation analysis of lactation women’s diet and water-soluble vitamins, minerals content in breast milk in a north county]. Chinese Nutrition Society. 2011;317–22.

164. Zhao A, Ning Y, Zhang Y, Yang X, Wang J, Li W, et al. Mineral compositions in breast milk of healthy Chinese lactating women in urban areas and its associated factors. Chin Med J (Engl). 2014;127(14):2643–8.

165. Zimmerman DR, Goldstein L, Lahat E, Braunstein R, Stahi D, Bar-Haim A, et al. Effect of a 24+ hour fast on breast milk composition. J Hum Lact. 2009 May;25(2):194–8.

166. Abdulrazzaq YM, Osman N, Nagelkerke N, Kosanovic M, Adem A. Trace element composition of plasma and breast milk of well-nourished women. J Environ Sci Health A Tox Hazard Subst Environ Eng. 2008 Feb 15;43(3):329–34.

167. Ahmed L. Antioxidant Micronutrient Profile (Vitamin E, C, A, Copper, Zinc, Iron) of Colostrum: Association with Maternal Characteristics. Journal of Tropical Pediatrics. 2004 Dec 1;50(6):357–8.

168. Al-Awadi FM, Srikumar TS. Trace-element status in milk and plasma of Kuwaiti and non-Kuwaiti lactating mothers. Nutrition. 2000 Nov;16(11–12):1069–73.

169. Al-Awadi FM, Srikumar TS. Trace elements and their distribution in protein fractions of camel milk in comparison to other commonly consumed milks. Journal of Dairy Research. 2001 Aug;68(3):463–9.

170. Al-Terehi M, Zaidan HK, Al-Mamoori AMJ, Al-Saadi AH, Harjan I. Effective of different factors on trace elements concentrations in Iraqi lactating mother’s milk. IJPRIF. 2015;8(10):151–7.

171. Alam S, Hennigar SR, Gallagher C, Soybel DI, Kelleher SL. Exome Sequencing of SLC30A2 Identifies Novel Loss- and Gain-of-Function Variants Associated with Breast Cell Dysfunction. J Mammary Gland Biol Neoplasia. 2015 Dec;20(3–4):159–72.

172. Almeida AA, Lopes CMPV, Silva AMS, Barrado E. Trace elements in human milk: correlation with blood levels, inter-element correlations and changes in concentration during the first month of lactation. J Trace Elem Med Biol. 2008;22(3):196–205.

173. Alves Peixoto RR, Bianchi Codo CR, Lacerda Sanches V, Guiraldelo TC, Ferreira da Silva F, Ribessi RL, et al. Trace mineral composition of human breast milk from Brazilian mothers. J Trace Elem Med Biol. 2019 Jul;54:199–205.

174. Anderson RR. Comparison of trace elements in milk of four species. J Dairy Sci. 1992 Nov;75(11):3050–5.

175. Anderson RR. Longitudinal changes of trace elements in human milk during the first 5 months of lactation. Nutrition Research. 1993 May 1;13(5):499–510.

176. Aquilio E, Spagnoli R, Seri S, Bottone G, Spennati G. Trace element content in human milk during lactation of preterm newborns. Biol Trace Elem Res. 1996 Jan;51(1):63–70.

177. Arnaud J, Favier A. Determination of ultrafiltrable zinc in human milk by electrothermal atomic absorption spectrometry. Analyst. 1992 Oct;117(10):1593–8.

178. Arnaud J, Prual A, Preziosi P, Cherouvrier F, Favier A, Galan P, et al. Effect of iron supplementation during pregnancy on trace element (Cu, Se, Zn) concentrations in serum and breast milk from Nigerian women. Ann Nutr Metab. 1993;37(5):262–71.

179. Arnaud J, Favier A. Copper, iron, manganese and zinc contents in human colostrum and transitory milk of French women. Sci Total Environ. 1995 Jan 6;159(1):9–15.

180. Atinmo T, Omololu A. Trace element content of breastmilk from mothers of preterm infants in Nigeria. Early Hum Dev. 1982 Jul;6(3):309–13.

181. Aumeistere L, Ciproviča I, Zavadska D, Bavrins K, Borisova A. Zinc Content in Breast Milk and Its Association with Maternal Diet. Nutrients. 2018 Oct 5;10(10).

182. Bamgbose O, Opeolu BO, Bamgbose JT. Levels of zinc in breast milk of selected women in Abeokuta township, Ogun state, Nigeria. Trace Elements and Electrolytes. 2012;29(3):149–53.

183. Benemariya H, Robberecht H, Deelstra H. Copper, zinc and selenium concentrations in milk from middle-class women in Burundi (Africa) throughout the first 10 months of lactation. Science of The Total Environment. 1995 Mar 15;164(2):161–74.

184. Robberecht H, Benemariya H, Deelstra H. Daily dietary intake of copper, zinc, and selenium of exclusively breast-fed infants of middle-class women in Burundi, Africa. Biol Trace Elem Res. 1995 Sep;49(2–3):151–9.

185. Biego GH, Joyeux M, Hartemann P, Debry G. Determination of mineral contents in different kinds of milk and estimation of dietary intake in infants. Food Addit Contam. 1998 Oct;15(7):775–81.

186. Bzikowska-Jura A, Sobieraj P, Michalska-Kacymirow M, Wesołowska A. Investigation of Iron and Zinc Concentrations in Human Milk in Correlation to Maternal Factors: An Observational Pilot Study in Poland. Nutrients. 2021 Jan 21;13(2):303.

187. Cardoso OO, Julião FC, Alves RIS, Baena AR, Díez IG, Suzuki MN, et al. Concentration profiles of metals in breast milk, drinking water, and soil: relationship between matrices. Biol Trace Elem Res. 2014 Jul;160(1):116–22.

188. Casey CE, Hambidge KM, Neville MC. Studies in human lactation: zinc, copper, manganese and chromium in human milk in the first month of lactation. Am J Clin Nutr. 1985 Jun;41(6):1193–200.

189. Casey CE, Neville MC, Hambidge KM. Studies in human lactation: secretion of zinc, copper, and manganese in human milk. Am J Clin Nutr. 1989 May;49(5):773–85.

190. Çebi A, Şengül Ü. Toxic metal and trace element status in the breast milk of Turkish new-born mothers. J Trace Elem Med Biol [Internet]. 2022;74((Çebi A., cebiaysegul@hotmail.com) Giresun University, Faculty of Health Sciences, Giresun, Turkey). Available from: https://www.embase.com/search/results?subaction=viewrecord&id=L2020191207&from=export

191. Chierici R, Saccomandi D, Vigi V. Dietary supplements for the lactating mother: influence on the trace element content of milk. Acta Paediatr Suppl. 1999 Aug;88(430):7–13.

192. Cinar N, Ozdemir S, Yucel O, Ucar F. In which regions is breast-feeding safer from the impact of toxic elements from the environment? Bosn J Basic Med Sci. 2011 Nov;11(4):234–9.

193. Cissé AS, Dossou N, Ndiaye M, Guèye AL, Diop EHI, Diaham B, et al. Stable isotope aided evaluation of community nutrition program: effect of food supplementation schemes on maternal and infant nutritional status. Food Nutr Bull. 2002 Sep;23(3 Suppl):169–73.

194. Santos da Costa RS, Tavares do Carmo M das G, Saunders C, Lopes RT, O de Jesus EF, Simabuco SM. Trace Elements Content of Colostrum Milk in Brazil. Journal of Food Composition and Analysis. 2002 Feb;15(1):27–33.

195. da Costa RSS, do Carmo MGT, Saunders C, de Jesus EFO, Lopes RT, Simabuco SM. Characterization of iron, copper and zinc levels in the colostrum of mothers of term and pre-term infants before and after pasteurization. Int J Food Sci Nutr. 2003 Mar;54(2):111–7.

196. Dang HS, Jaiswal DD, Wadhwani CN, Somasunderam S, Dacosta H. Infants with a congenital anomaly and the concentration of Mo, As, Mn, Zn and Cu in the mother’s milk. Science of The Total Environment. 1983 Mar;27(1):43–7.

197. Dang HS, Jaiswal DD, Somasundaram S, Deshpande A, Dacosta H. Concentrations of four essential trace elements in breast milk of mothers from two socio-economic groups: preliminary observations. Sci Total Environ. 1984 Apr 5;35(1):85–9.

198. Dang HS, Jaiswal DD, Wadhwani CN, Somasunderam S, Dacosta H. Breast feeding: Mo, As, Mn, Zn and Cu concentrations in milk of economically poor Indian tribal and urban women. Science of The Total Environment. 1985 Aug;44(2):177–82.

199. de Figueiredo CSM, Palhares DB, Melnikov P, Moura AJ da CM, dos Santos SC. Zinc and copper concentrations in human preterm milk. Biol Trace Elem Res. 2010 Jul;136(1):1–7.

200. de Oliveira Trinta V, Padilha P de C, Petronilho S, Santelli RE, Braz BF, Freire AS, et al. Total metal content and chemical speciation analysis of iron, copper, zinc and iodine in human breast milk using high-performance liquid chromatography separation and inductively coupled plasma mass spectrometry detection. Food Chemistry. 2020 Oct;326:126978.

201. Dempster WS, Pocock FH, Kirsten G, Watermeyer S, Heese HD. Measurement of zinc and copper levels in breast milk. S Afr Med J. 1981 May 23;59(22):785–7.

202. Dhonukshe-Rutten RAM, Vossenaar M, West CE, Schümann K, Bulux J, Solomons NW. Day-to-day variations in iron, zinc and copper in breast milk of Guatemalan mothers. J Pediatr Gastroenterol Nutr. 2005 Feb;40(2):128–34; discussion 120-121.

203. Dijkhuizen MA, Wieringa FT, West CE, Muherdiyantiningsih null, Muhilal null. Concurrent micronutrient deficiencies in lactating mothers and their infants in Indonesia. Am J Clin Nutr. 2001 Apr;73(4):786–91.

204. Djurović D, Milisavljević B, Mugoša B, Lugonja N, Miletić S, Spasić S, et al. Zinc concentrations in human milk and infant serum during the first six months of lactation. J Trace Elem Med Biol. 2017 May;41:75–8.

205. Domellöf M, Lönnerdal B, Dewey KG, Cohen RJ, Hernell O. Iron, zinc, and copper concentrations in breast milk are independent of maternal mineral status. Am J Clin Nutr. 2004 Jan;79(1):111–5.

206. Donangelo CM, Trugo NM, Koury JC, Barreto Silva MI, Freitas LA, Feldheim W, et al. Iron, zinc, folate and vitamin B12 nutritional status and milk composition of low-income Brazilian mothers. Eur J Clin Nutr. 1989 Apr;43(4):253–66.

207. Doneray H, Olcaysu E, Yildirim A, Ozden A. The effect of the zinc concentration in breast milk on neonatal weight gain. J Trace Elem Med Biol. 2017 May;41:32–5.

208. Dorea JG, Horner MR, Campanate ML. Lacteal zinc and copper in relation to volume, total ash and energy during the first three months of lactation of Brazilian women. Acta Paediatr Scand. 1985 Nov;74(6):891–6.

209. Dorea J, Costa T, Marques A. Effects of contraceptives on mother’s serum and milk zinc. The Journal of Nutritional Biochemistry. 1993 Feb;4(2):86–91.

210. Dórea JG. Zinc and copper in breast-milk and home-prepared milk fed to urban infants from low-income families. The Journal of Trace Elements in Experimental Medicine. 2002 Jan 1;15(3):123–9.

211. Dumrongwongsiri O, Suthutvoravut U, Chatvutinun S, Phoonlabdacha P, Sangcakul A, Siripinyanond A, et al. Maternal zinc status is associated with breast milk zinc concentration and zinc status in breastfed infants aged 4-6 months. Asia Pac J Clin Nutr. 2015;24(2):273–80.

212. Dumrongwongsiri O, Winichagoon P, Chongviriyaphan N, Suthutvoravut U, Grote V, Koletzko B. Zinc and iron adequacy and relative importance of zinc/iron storage and intakes among breastfed infants. Maternal & Child Nutrition [Internet]. 2022 Jan [cited 2022 Nov 26];18(1). Available from: https://onlinelibrary.wiley.com/doi/10.1111/mcn.13268

213. Duncan JR. Zinc nutriture in pregnant and lactating women in different population groups. S Afr Med J. 1988 Feb 6;73(3):160–2.

214. Đurović D, Milisavljević B, Nedović-Vuković M, Potkonjak B, Spasić S, Vrvić M. Determination of Microelements in Human Milk and Infant Formula Without Digestion by ICP-OES. Acta Chim Slov. 2017 Jun;64(2):276–82.

215. Edem VF, Akintunde K, Adelaja YA, Nwozo SO, Charles-Davies M. Zinc, lead, and cadmium levels in serum and milk of lactating women in Ibadan, Nigeria. Toxicol Ind Health. 2017 Jan;33(1):28–35.

216. Ehsani P, Afsahri P, Soori H, Zahedi Asl S. [Effect of iron supplementation on zinc and magnesium concentrations in maternal milk and plasma]. Koomesh. 2009;10(4):229–36.

217. Ejezie FE, Nwagha UI. Zinc Concentration during Pregnancy and Lactation in Enugu, South-East Nigeria. Ann Med Health Sci Res. 2011;1(1):69–76.

218. Ezz El Din ZM, Abd El Ghaffar S, El Gabry EK, Fahmi WA, Bedair RF. Is stored expressed breast milk an alternative for working Egyptian mothers? East Mediterr Health J. 2004 Nov;10(6):815–21.

219. Elmastas M, Can M, Uzun S, Aboul‐Enein HY. Determinations of Copper, Zinc, Cadmium, and Nickel in Cows’, Goats’, Ewes’, and Human Milk Samples Using Flame Atomic Absorption Spectrometry (FAAS) Microwave Digestion. Analytical Letters. 2005 Jan;38(1):157–65.

220. Ergül AB, Öztürk MA, Leblebici Z. Erken ve zamanında doğmuş bebeklerin anne sütlerinin çinko, bakır ve demir düzeylerinin karşılaştırılması. Türk Pediatri Arşivi. 2010 Sep 15;45(3):272–9.

221. Ezechukwu CC, Ubom G, Airede KI. Zinc and copper levels of mature breast milk of healthy lactating women within the first six months of lactation. Journal of Biomedical Investigation. 2004;2(1):10–6.

222. Feeley RM, Eitenmiller RR, Jones JB, Barnhart H. Copper, iron, and zinc contents of human milk at early stages of lactation. Am J Clin Nutr. 1983 Mar;37(3):443–8.

223. Fernández-Menéndez S, Fernández-Sánchez ML, Fernández-Colomer B, de la Flor St Remy RR, Cotallo GDC, Freire AS, et al. Total zinc quantification by inductively coupled plasma-mass spectrometry and its speciation by size exclusion chromatography-inductively coupled plasma-mass spectrometry in human milk and commercial formulas: Importance in infant nutrition. J Chromatogr A. 2016 Jan 8;1428:246–54.

224. Fouché C, van Niekerk E, du Plessis LM. Differences in Breast Milk Composition of HIV-Infected and HIV-Uninfected Mothers of Premature Infants: Effects of Antiretroviral Therapy. Breastfeed Med. 2016 Nov;11:455–60.

225. Frković A, Medugorac B, Alebić-Juretić A. Zinc levels in human milk and umbilical cord blood. Sci Total Environ. 1996 Dec 2;192(2):207–12.

226. Fung EB, Ritchie LD, Woodhouse LR, Roehl R, King JC. Zinc absorption in women during pregnancy and lactation: a longitudinal study. Am J Clin Nutr. 1997 Jul;66(1):80–8.

227. Gross R, Hänsel H, Schultink W, Shrimpton R, Matulessi P, Gross G, et al. Moderate zinc and vitamin A deficiency in breast milk of mothers from East-Jakarta. Eur J Clin Nutr. 1998 Dec;52(12):884–90.

228. Hampel D, Shahab-Ferdows S, Gertz E, Flax VL, Adair LS, Bentley ME, et al. The effects of a lipid-based nutrient supplement and antiretroviral therapy in a randomized controlled trial on iron, copper, and zinc in milk from HIV-infected Malawian mothers and associations with maternal and infant biomarkers. Matern Child Nutr. 2018 Apr;14(2):e12503.

229. Hannan MA, Dogadkin NN, Ashur IA, Markus WM. Copper, selenium, and zinc concentrations in human milk during the first three weeks of lactation. Biol Trace Elem Res. 2005 Oct;107(1):11–20.

230. Hannan MA, Faraji B, Tanguma J, Longoria N, Rodriguez RC. Maternal milk concentration of zinc, iron, selenium, and iodine and its relationship to dietary intakes. Biol Trace Elem Res. 2009 Jan;127(1):6–15.

231. Higashi A, Ikeda T, Uehara I, Matsuda I. Zinc and copper contents in breast milk of Japanese women. Tohoku J Exp Med. 1982 May;137(1):41–7.

232. Hunt K, Malek A, DellaValle D, Greenberg D, St. Peter J, Marriott B. Low calorie sweetener and macronutrient intake in the US adult population:NHANES 2007-2010. FASEB Journal. 29:2015.

233. Islam MM, Brown KH. Zinc transferred through breast milk does not differ between appropriate- and small-for-gestational-age, predominantly breast-fed Bangladeshi infants. J Nutr. 2014 May;144(5):771–6.

234. Jagodic M, Potočnik D, Snoj Tratnik J, Mazej D, Pavlin M, Trdin A, et al. Selected elements and fatty acid composition in human milk as indicators of seafood dietary habits. Environ Res. 2020 Jan;180:108820.

235. Jariwala M, Suvarna S, Kiran Kumar G, Amin A, Udas AC. Study of the concentration of trace elements fe, zn, cu, se and their correlation in maternal serum, cord serum and colostrums. Indian J Clin Biochem. 2014 Apr;29(2):181–8.

236. Kantola M, Vartiainen T. Changes in selenium, zinc, copper and cadmium contents in human milk during the time when selenium has been supplemented to fertilizers in Finland. J Trace Elem Med Biol. 2001;15(1):11–7.

237. Karra MV, Kirksey A, Galal O, Bassily NS, Harrison GG, Jerome NW. Effect of short-term oral zinc supplementation on theconcentration of zinc in milk from american and egyptian women. Nutrition Research. 1989 May;9(5):471–8.

238. Keizer SE, Gibson RS, O’Connor DL. Postpartum folic acid supplementation of adolescents: impact on maternal folate and zinc status and milk composition. Am J Clin Nutr. 1995 Aug;62(2):377–84.

239. Khaghani S, Ezzatpanah H, Mazhari N, Givianrad MH, Mirmiranpour H, Sadrabadi FS. Zinc and copper concentrations in human milk and infant formulas. Iran J Pediatr. 2010 Mar;20(1):53–7.

240. Kim SY, Park JH, Kim EAR, Lee-Kim YC. Longitudinal study on trace mineral compositions (selenium, zinc, copper, manganese) in Korean human preterm milk. J Korean Med Sci. 2012 May;27(5):532–6.

241. Kirsten GF, Heese HD, Watermeyer S, Dempster WS, Pocock F, Varkevisser H. Zinc and copper levels in the breast-milk of Cape Town mothers. S Afr Med J. 1985 Sep 14;68(6):402–5.

242. Kosanovic M, Adem A, Jokanovic M, Abdulrazzaq YM. Simultaneous Determination of Cadmium, Mercury, Lead, Arsenic, Copper, and Zinc in Human Breast Milk by ICP‐MS/Microwave Digestion. Analytical Letters. 2008 Feb;41(3):406–16.

243. Krebs NF, Hambidge KM, Jacobs MA, Rasbach JO. The effects of a dietary zinc supplement during lactation on longitudinal changes in maternal zinc status and milk zinc concentrations. Am J Clin Nutr. 1985 Mar;41(3):560–70.

244. Krebs NF, Hambidge KM, Jacobs MA, Mylet S. Zinc in human milk: diurnal and within-feed patterns. J Pediatr Gastroenterol Nutr. 1985 Apr;4(2):227–9.

245. Krebs NF, Reidinger CJ, Hartley S, Robertson AD, Hambidge KM. Zinc supplementation during lactation: effects on maternal status and milk zinc concentrations. Am J Clin Nutr. 1995 May;61(5):1030–6.

246. Lamounier JA, Danelluzzi JC, Vannucchi H. Zinc concentrations in human milk during lactation: a 6-month longitudinal study in southern Brazil. J Trop Pediatr. 1989 Feb;35(1):31–4.

247. Lastre-Amell G, Carrero González CM, Delgado Diloreto FB, Suarez-Villa M, Granadillo Morán V, Orostegui Santander MA. [Concentrations of trace elements like copper and zinc in breast, cow’s and goat’s milk]. Revista Cubana de Pediatría. 2020;92(2):e649.

248. Lauber E, Reinhardt M. Studies on the quality of breast milk during 23 months of lactation in a rural community of the Ivory Coast. Am J Clin Nutr. 1979 May;32(5):1159–73.

249. Leotsinidis M, Alexopoulos A, Kostopoulou-Farri E. Toxic and essential trace elements in human milk from Greek lactating women: association with dietary habits and other factors. Chemosphere. 2005 Oct;61(2):238–47.

250. Li F, Mo JL. [Influence of dietary intervention on the dietary nutrition status of lactating women and the concentrations of zinc, copper, and magnesium in breast milk]. Chinese Journal of New Clinical Medicine. 2013;6(6):583–6.

251. Lönnerdal B, Hoffman B, Hurley LS. Zinc and copper binding proteins in human milk. Am J Clin Nutr. 1982 Dec;36(6):1170–6.

252. Lönnerdal B, Zavaleta N, Kusunoki L, Lanata CF, Peerson JM, Brown KH. Effect of postpartum maternal infection on proteins and trace elements in colostrum and early milk. Acta Paediatr. 1996 May;85(5):537–42.

253. Maeda T, Tanaka T, Ohshiro H, Funakawa K, Nose T, Imai S, et al. [Zinc and copper concentrations in breast milk and maternal serum in the postpartum period]. Nihon Eiseigaku Zasshi. 1990 Aug;45(3):781–7.

254. Mahdavi R, Nikniaz L, Gayemmagami SJ. Association between zinc, copper, and iron concentrations in breast milk and growth of healthy infants in Tabriz, Iran. Biol Trace Elem Res. 2010 Jun;135(1–3):174–81.

255. Mandić Z, Mandić ML, Grgić J, Grgić Z, Klapec T, Primorac L, et al. Copper and zinc content in human milk in Croatia. Eur J Epidemiol. 1997 Feb;13(2):185–8.

256. Martínez MÁ, Castro I, Rovira J, Ares S, Rodríguez JM, Cunha SC, et al. Early-life intake of major trace elements, bisphenol A, tetrabromobisphenol A and fatty acids: Comparing human milk and commercial infant formulas. Environ Res. 2019 Feb;169:246–55.

257. Matos C, Moutinho C, Balcão V, Almeida C, Ribeiro M, Marques AF, et al. Total antioxidant activity and trace elements in human milk: the first 4 months of breast-feeding. European Food Research and Technology. 2009 Dec;230(2):201–8.

258. Matsuda I, Higashi A, Ikeda T, Uehara I, Kuroki Y. Effects of zinc and copper content of formulas on growth and on the concentration of zinc and copper in serum and hair. J Pediatr Gastroenterol Nutr. 1984 Jun;3(3):421–5.

259. Mbofung CM, Atinmo T, Omololu A. Mineral content of colostrum and mature milk of lactating Nigerian women as influenced by stage of lactation. Nutr Rep Int. 1984 Nov;30(5):1137–46.

260. Mello-Neto J, Rondó PHC, Oshiiwa M, Morgano MA, Zacari CZ, Domingues S. The influence of maternal factors on the concentration of vitamin A in mature breast milk. Clinical Nutrition. 2009 Apr;28(2):178–81.

261. Mello-Neto J, Rondó PHC, Oshiiwa M, Morgano MA, Zacari CZ, dos Santos ML. Iron supplementation in pregnancy and breastfeeding and iron, copper and zinc status of lactating women from a human milk bank. J Trop Pediatr. 2013 Apr;59(2):140–4.

262. Melnikov P, da Cruz Montes Moura AJ, Batista Palhares D, Martimbianco de Figueiredo CS. Zinc and copper in colostrum. Indian Pediatr. 2007 May;44(5):355–7.

263. Mendelson RA, Anderson GH, Bryan MH. Zinc, copper and iron content of milk from mothers of preterm and full-term infants. Early Hum Dev. 1982 Apr;6(2):145–51.

264. Milnerowicz H, Chmarek M. Effect of smoking on concentrations of cadmium, copper, iron and zinc in early transitional human milk. Acta Toxicologica. 2003;11(2):85–91.

265. Mohd-Taufek N, Cartwright D, Davies M, Hewavitharana AK, Koorts P, McConachy H, et al. The effect of pasteurization on trace elements in donor breast milk. J Perinatol. 2016 Oct;36(10):897–900.

266. Moran JR, Vaughan R, Stroop S, Coy S, Johnston H, Greene HL. Concentrations and total daily output of micronutrients in breast milk of mothers delivering preterm: a longitudinal study. J Pediatr Gastroenterol Nutr. 1983 Nov;2(4):629–34.

267. Moser PB, Reynolds RD. Dietary zinc intake and zinc concentrations of plasma, erythrocytes, and breast milk in antepartum and postpartum lactating and nonlactating women: a longitudinal study. Am J Clin Nutr. 1983 Jul;38(1):101–8.

268. Moser PB, Reynolds RD, Acharya S, Howard MP, Andon MB, Lewis SA. Copper, iron, zinc, and selenium dietary intake and status of Nepalese lactating women and their breast-fed infants. Am J Clin Nutr. 1988 Apr;47(4):729–34.

269. Moser-Veillon PB, Reynolds RD. A longitudinal study of pyridoxine and zinc supplementation of lactating women. Am J Clin Nutr. 1990 Jul;52(1):135–41.

270. Motoyama K, Isojima T, Sato Y, Aihara A, Asakura H, Hiraike H, et al. Trace element levels in mature breast milk of recently lactating Japanese women. Pediatr Int. 2021 Aug;63(8):910–7.

271. Murthy GK, Rhea US. Cadmium, copper, iron, lead, manganese, and zinc in evaporated milk, infant products, and human milk. J Dairy Sci. 1971 Jul;54(7):1001–5.

272. Nakamori M, Ninh NX, Isomura H, Yoshiike N, Hien VTT, Nhug BT, et al. Nutritional status of lactating mothers and their breast milk concentration of iron, zinc and copper in rural Vietnam. J Nutr Sci Vitaminol (Tokyo). 2009 Aug;55(4):338–45.

273. Narang APS, Bains HS, Kansal S, Singh D. Comparative study of trace elements of human milk in preterm and term mothers. Trace Elements and Electrolytes. 2006 Apr 1;23(04):99–102.

274. Ohtake M, Chiba R, Mochizuki K, Tada K. Zinc and copper concentrations in human milk and in serum from exclusively-breast-fed infants during the first 3 months of life. Tohoku J Exp Med. 1981 Dec;135(4):335–43.

275. Ohtake M, Tamura T. Changes in zinc and copper concentrations in breast milk and blood of Japanese women during lactation. J Nutr Sci Vitaminol (Tokyo). 1993 Apr;39(2):189–200.

276. Okolo SN, Onwuanaku C, Okonji M, VanderJagt DJ, Millson M, Churchwell C, et al. Concentration of eight trace minerals in milk and sera of mother-infant pairs in northern Nigeria. J Trop Pediatr. 2000 Jun;46(3):160–2.

277. Ortega RM, Andrés P, Martínez RM, López-Sobaler AM, Quintas ME. Zinc levels in maternal milk: the influence of nutritional status with respect to zinc during the third trimester of pregnancy. Eur J Clin Nutr. 1997 Apr;51(4):253–8.

278. Orun E, Yalcin SS, Aykut O, Orhan G, Morgil GK. Zinc and copper concentrations in breastmilk at the second month of lactation. Indian Pediatr. 2012 Feb;49(2):133–5.

279. Osredkar J, Geršak ŽM, Karas Kuželički N, Snoj Tratnik J, Mazej D, Falnoga I, et al. Association of Zn and Cu Levels in Cord Blood and Maternal Milk with Pregnancy Outcomes among the Slovenian Population. Nutrients [Internet]. 2022;14(21). Available from: https://www.embase.com/search/results?subaction=viewrecord&id=L2020042539&from=export

280. Picciano MF, Guthrie HA. Copper, iron, and zinc contents of mature human milk. Am J Clin Nutr. 1976 Mar;29(3):242–54.

281. Qian L, Wang B, Tang N, Zhang W, Cai W. Polymorphisms of SLC30A2 and selected perinatal factors associated with low milk zinc in Chinese breastfeeding women. Early Hum Dev. 2012 Aug;88(8):663–8.

282. Rajalakshmi K, Srikantia SG. Copper, zinc, and magnesium content of breast milk of Indian women. Am J Clin Nutr. 1980 Mar;33(3):664–9.

283. Rodríguez Rodríguez EM, Sanz Alaejos M, Díaz Romero C. Concentrations of iron, copper and zinc in human milk and powdered infant formula. Int J Food Sci Nutr. 2000 Sep;51(5):373–80.

284. Rossipal E, Krachler M. Pattern of trace elements in human milk during the course of lactation. Nutrition Research. 1998 Jan;18(1):11–24.

285. Krachler M, Li FS, Rossipal E, Irgolic KJ. Changes in the concentrations of trace elements in human milk during lactation. J Trace Elem Med Biol. 1998 Nov;12(3):159–76.

286. Salmenperä L, Perheentupa J, Näntö V, Siimes MA. Low zinc intake during exclusive breast-feeding does not impair growth. J Pediatr Gastroenterol Nutr. 1994 Apr;18(3):361–70.

287. Samuel TM, Thomas T, Thankachan P, Bhat S, Virtanen SM, Kurpad AV. Breast milk zinc transfer and early post-natal growth among urban South Indian term infants using measures of breast milk volume and breast milk zinc concentrations. Matern Child Nutr. 2014 Jul;10(3):398–409.

288. Samuel TM, Thomas T, Bhat S, Kurpad AV. Are infants born in baby-friendly hospitals being exclusively breastfed until 6 months of age? Eur J Clin Nutr. 2012 Apr;66(4):459–65.

289. Sazawal S, Black RE, Dhingra P, Jalla S, Krebs N, Malik P, et al. Zinc Supplementation does not Affect the Breast Milk Zinc Concentration of Lactating Women Belonging to Low Socioeconomic Population. J Hum Nutr Food Sci. 2013;1(2):1014.

290. Severi C, Hambidge M, Krebs N, Alonso R, Atalah E. Zinc in plasma and breast milk in adolescents and adults in pregnancy and pospartum: a cohort study in Uruguay. Nutr Hosp. 2013 Feb;28(1):223–8.

291. Shaaban SY, El-Hodhod MAA, Nassar MF, Hegazy AET, El-Arab SE, Shaheen FM. Zinc status of lactating Egyptian mothers and their infants: effect of maternal zinc supplementation. Nutrition Research. 2005 Jan;25(1):45–53.

292. Sharda B, Bhandari B, Bhandari LM. Copper, zinc, magnesium and cadmium levels of breast milk of Indian women. Trans R Soc Trop Med Hyg. 1983;77(2):201–3.

293. Sharda B, Adhikari R, Ajmera M, Gambhir R, Singh PP. Zinc and copper in preterm neonates: relationship with breast milk. Indian J Pediatr. 1999 Oct;66(5):685–95.

294. Shawahna R. Predictors of Breast Milk Zinc Levels Among Breastfeeding Women in Palestine: a Cross-Sectional Study. Biol Trace Elem Res. 2022;200(11):4632–40.

295. Sian L, Krebs NF, Westcott JE, Fengliang L, Tong L, Miller LV, et al. Zinc homeostasis during lactation in a population with a low zinc intake. Am J Clin Nutr. 2002 Jan;75(1):99–103.

296. Sievers E, Oldigs HD, Dörner K, Schaub J. Longitudinal zinc balances in breast-fed and formula-fed infants. Acta Paediatr. 1992 Jan;81(1):1–6.

297. Silvestre MD, Lagarda MJ, Farré R, Martı́nez-Costa C, Brines J. Copper, iron and zinc determinations in human milk using FAAS with microwave digestion. Food Chemistry. 2000 Jan;68(1):95–9.

298. Silvestre MD, Lagarda MJ, Farré R, Martínez-Costa C, Brines J, Molina A, et al. A study of factors that may influence the determination of copper, iron, and zinc in human milk during sampling and in sample individuals. Biol Trace Elem Res. 2000 Sep;76(3):217–27.

299. Silvestre D, Martìnez-Costa C, Lagarda MJ, Brines J, Farré R, Clemente G. Copper, iron, and zinc contents in human milk during the first three months of lactation: a longitudinal study. Biol Trace Elem Res. 2001 Apr;80(1):1–11.

300. Simmer K, Ahmed S, Carlsson L, Thompson RP. Breast milk zinc and copper concentrations in Bangladesh. Br J Nutr. 1990 Jan;63(1):91–6.

301. Snoj Tratnik J, Falnoga I, Mazej D, Kocman D, Fajon V, Jagodic M, et al. Results of the first national human biomonitoring in Slovenia: Trace elements in men and lactating women, predictors of exposure and reference values. Int J Hyg Environ Health. 2019 Apr;222(3):563–82.

302. Tahboub YR, Massadeh AM, Al-Sheyab NA, El Shrafat D, Nsserat IA. Levels of Trace Elements in Human Breast Milk in Jordan: a Comparison with Infant Formula Milk Powder. Biol Trace Elem Res. 2021 Nov;199(11):4066–73.

303. Trugo NM, Donangelo CM, Koury JC, Silva MI, Freitas LA. Concentration and distribution pattern of selected micronutrients in preterm and term milk from urban Brazilian mothers during early lactation. Eur J Clin Nutr. 1988 Jun;42(6):497–507.

304. Tripathi RM, Raghunath R, Sastry VN, Krishnamoorthy TM. Daily intake of heavy metals by infants through milk and milk products. Sci Total Environ. 1999 Mar 9;227(2–3):229–35.

305. Turan S, Saygi S, Kilic Z, Acar O. Determination of heavy metal concentrations in human colostrum samples by electrothermal atomic absorption spectrophotometry. Journal of Tropical Pediatrics. 2001;47(2):81–5.

306. Ustundag B, Yilmaz E, Dogan Y, Akarsu S, Canatan H, Halifeoglu I, et al. Levels of cytokines (IL-1beta, IL-2, IL-6, IL-8, TNF-alpha) and trace elements (Zn, Cu) in breast milk from mothers of preterm and term infants. Mediators Inflamm. 2005 Dec 14;2005(6):331–6.

307. Van der Elst CW, Dempster WS, Woods DL, Heese HD. Serum zinc and copper in thin mothers, their breast milk and their infants. J Trop Pediatr. 1986 Jun;32(3):111–4.

308. Vuori E, Kuitunen P. The concentrations of copper and zinc in human milk. A longitudinal study. Acta Paediatr Scand. 1979 Jan;68(1):33–7.

309. Vuori E, Mäkinen SM, Kara R, Kuitunen P. The effects of the dietary intakes of copper, iron, manganese, and zinc on the trace element content of human milk. Am J Clin Nutr. 1980 Feb;33(2):227–31.

310. Wang YH, Li WD, Wang WD, Bai SZ. [The content change of Cu, Fe and Zn in breast milk and comparison with the cow milk]. J Inner Mongolia Univ National. 2001;16(3):249–50.

311. Wasowicz W, Gromadzinska J, Szram K, Rydzynski K, Cieslak J, Pietrzak Z. Selenium, zinc, and copper concentrations in the blood and milk of lactating women. Biol Trace Elem Res. 2001 Mar;79(3):221–33.

312. Winiarska-Mieczan A. Cadmium, lead, copper and zinc in breast milk in Poland. Biol Trace Elem Res. 2014 Jan;157(1):36–44.

313. Wu MQ, Wu DQ, Yang Y, Zhou CC, Yan CH. Impact of delivery mode on the levels of essential trace elements in breast milk. J Matern Fetal Neonatal Med. 2020 Oct;33(19):3293–9.

314. Xiang M, Harbige L, Zetterström R. Breast milk levels of zinc and ω-6 polyunsaturated fatty acids and growth of healthy Chinese infants. Acta Paediatrica. 2007 Mar;96(3):387–90.

315. Yalçin SS, Yurdakök K, Yalçin S, Engür-Karasimav D, Coşkun T. Maternal and environmental determinants of breast-milk mercury concentrations. Turk J Pediatr. 2010 Feb;52(1):1–9.

316. Yalçin SS, Baykan A, Yurdakök K, Yalçin S, Gücüş AI. The factors that affect milk-to-serum ratio for iron during early lactation. J Pediatr Hematol Oncol. 2009 Feb;31(2):85–90.

317. Young BE, Borman LL, Heinrich R, Long J, Pinney S, Westcott J, et al. Effect of Pooling Practices and Time Postpartum of Milk Donations on the Energy, Macronutrient, and Zinc Concentrations of Resultant Donor Human Milk Pools. J Pediatr. 2019 Nov;214:54–9.

318. Zapata C. Effect of iron supplementation during lactation on human milk composition. The Journal of Nutritional Biochemistry. 1994 Jul;5(7):331–7.

319. Zavaleta N, Lanata C, Butron B, Peerson JM, Brown KH, Lönnerdal B. Effect of acute maternal infection on quantity and composition of breast milk. Am J Clin Nutr. 1995 Sep;62(3):559–63.

320. Ala-Houhala M, Koskinen T, Parviainen MT, Visakorpi JK. 25-Hydroxyvitamin D and vitamin D in human milk: effects of supplementation and season. Am J Clin Nutr. 1988 Oct;48(4):1057–60.

321. Parviainen MT, Koskinen T, Ala-Houhala M, Visakorpi JK. A method for routine estimation of vitamin D activity in human and bovine milk. Acta Vitaminol Enzymol. 1984;6(3):211–9.

322. Amukele TK, Soko D, Katundu P, Kamanga M, Sun J, Kumwenda NI, et al. Vitamin D levels in Malawian infants from birth to 24 months. Archives of Disease in Childhood. 2013 Mar 1;98(3):180–3.

323. Hoofnagle AN, Laha TJ, Donaldson TF. A rubber transfer gasket to improve the throughput of liquid-liquid extraction in 96-well plates: application to vitamin D testing. J Chromatogr B Analyt Technol Biomed Life Sci. 2010 Jun 1;878(19):1639–42.

324. Atkinson SA, Reinhardt TA, Hollis BW. Vitamin D activity in maternal plasma and milk in relation to gestational stage at delivery. Nutrition Research. 1987 Oct;7(10):1005–11.

325. Hollis BW, Roos BA, Lambert PW. Vitamin D in plasma: quantitation by a nonequilibrium ligand binding assay. Steroids. 1981 Jun;37(6):609–19.

326. Ballester I, Cortes E, Moya M, Campello MJ. Improved method for quantifying vitamin D in proprietary infants’ formulas and in breast milk. Clin Chem. 1987 Jun;33(6):796–9.

327. Hollis BW. Individual quantitation of vitamin D2, vitamin D3, 25-hydroxyvitamin D2, and 25-hydroxyvitamin D3 in human milk. Anal Biochem. 1983 May;131(1):211–9.

328. Dawodu A, Salameh KM, Al-Janahi NS, Bener A, Elkum N. The Effect of High-Dose Postpartum Maternal Vitamin D Supplementation Alone Compared with Maternal Plus Infant Vitamin D Supplementation in Breastfeeding Infants in a High-Risk Population. A Randomized Controlled Trial. Nutrients. 2019 Jul 17;11(7).

329. Wall CR, Stewart AW, Camargo CA, Scragg R, Mitchell EA, Ekeroma A, et al. Vitamin D activity of breast milk in women randomly assigned to vitamin D3 supplementation during pregnancy. Am J Clin Nutr. 2016 Feb;103(2):382–8.

330. Demers-Mathieu V, Lavangnananda S, Medo E. Influence of Vitamin D3 Levels and T Cell-Related Cytokines in Human Milk on Coronavirus Disease 2019 Infection in Lactating Women. Breastfeed Med. 2021 Dec;16(12):995–1003.

331. Ferreiro-Vera C, Priego-Capote F, Luque de Castro MD. An approach for quantitative analysis of vitamins D and B9 and their metabolites in human biofluids by on-line orthogonal sample preparation and sequential mass spectrometry detection. Analyst. 2013 Apr 7;138(7):2146–55.

332. Gjerde J, Kjellevold M, Dahl L, Berg T, Bøkevoll A, Markhus MW. Validation and Determination of 25(OH) Vitamin D and 3-Epi25(OH)D3 in Breastmilk and Maternal- and Infant Plasma during Breastfeeding. Nutrients. 2020 Jul 29;12(8).

333. Gomes FP, Shaw PN, Whitfield K, Koorts P, McConachy H, Hewavitharana AK. Effect of pasteurisation on the concentrations of vitamin D compounds in donor breastmilk. International Journal of Food Sciences and Nutrition. 2016 Jan 2;67(1):16–9.

334. Gomes FP, Shaw PN, Whitfield K, Hewavitharana AK. Simultaneous quantitative analysis of eight vitamin D analogues in milk using liquid chromatography–tandem mass spectrometry. Analytica Chimica Acta. 2015 Sep;891:211–20.

335. Greer FR, Ho M, Dodson D, Tsang RC. Lack of 25-hydroxyvitamin D and 1,25-dihydroxyvitamin D in human milk. J Pediatr. 1981 Aug;99(2):233–5.

336. Greer FR, Hollis BW, Cripps DJ, Tsang RC. Effects of maternal ultraviolet B irradiation on vitamin D content of human milk. J Pediatr. 1984 Sep;105(3):431–3.

337. Hollis BW, Pittard WB, Reinhardt TA. Relationships among vitamin D, 25-hydroxyvitamin D, and vitamin D-binding protein concentrations in the plasma and milk of human subjects. J Clin Endocrinol Metab. 1986 Jan;62(1):41–4.

338. Hollis BW, Wagner CL. Vitamin D requirements during lactation: high-dose maternal supplementation as therapy to prevent hypovitaminosis D for both the mother and the nursing infant. Am J Clin Nutr. 2004 Dec;80(6 Suppl):1752S-8S.

339. Hollis BW, Pittard WB. Evaluation of the total fetomaternal vitamin D relationships at term: evidence for racial differences. J Clin Endocrinol Metab. 1984 Oct;59(4):652–7.

340. Hollis BW, Kamerud JQ, Selvaag SR, Lorenz JD, Napoli JL. Determination of vitamin D status by radioimmunoassay with an 125I-labeled tracer. Clin Chem. 1993 Mar;39(3):529–33.

341. Hoogenboezem T, Degenhart HJ, de Muinck Keizer-Schrama SM, Bouillon R, Grose WF, Hackeng WH, et al. Vitamin D metabolism in breast-fed infants and their mothers. Pediatr Res. 1989 Jun;25(6):623–8.

342. Kamao M, Tsugawa N, Suhara Y, Okano T. Determination of Fat-Soluble Vitamins in Human Plasma, Breast Milk and Food Samples: Application in Nutrition Survey for Establishment of “Dietary Reference Intakes for Japanese.” Journal of Health Science. 2007;53(3):257–62.

343. Kamao M, Tsugawa N, Suhara Y, Wada A, Mori T, Murata K, et al. Quantification of fat-soluble vitamins in human breast milk by liquid chromatography-tandem mass spectrometry. J Chromatogr B Analyt Technol Biomed Life Sci. 2007 Nov 15;859(2):192–200.

344. Khaghani S, Pasalar P, Nowrouzi A. Effect of Progesterone­only Contraception on Vitamin D in Human Milk. Iran J Ped. 2008;18(1):25–30.

345. Jan Mohamed HJ, Rowan A, Fong B, Loy SL. Maternal serum and breast milk vitamin D levels: findings from the Universiti Sains Malaysia Pregnancy Cohort Study. PLoS One. 2014;9(7):e100705.

346. Lewis JG, Elder PA. Serum 25-OH vitamin D2 and D3 are stable under exaggerated conditions. Clin Chem. 2008 Nov;54(11):1931–2.

347. Moya M, Juste M, Cortés E, Carratalá F. Fatty acid composition of mature breast milk according to the mothers diet during pregnancy. Adv Exp Med Biol. 2000;478:405–6.

348. Nguyen MTT, Kim J, Lee H, Won S, Kim Y, Jung JA, et al. A Comparison of Vitamin and Lutein Concentrations in Breast Milk from Four Asian Countries. Nutrients. 2020 Jun 17;12(6):1794.

349. Niramitmahapanya S, Kaoiean S, Sangtawesin V, Patanaprapan A, Bordeerat NK, Deerochanawong C. Correlation of 25-Hydroxyvitamin D Levels in Serum vs. Breastmilk in Vitamin D-Supplementation Breastfeeding Women during Lactation: Randomized Double Blinded Control Trial. J Med Assoc Thai. 2017 Feb;100 Suppl 1:S165-171.

350. Nishimura K, Shima M, Tsugawa N, Matsumoto S, Hirai H, Santo Y, et al. Long-term hospitalization during pregnancy is a risk factor for vitamin D deficiency in neonates. J Bone Miner Metab. 2003;21(2):103–8.

351. Takeuchi A, Okano T, Tsugawa N, Tasaka Y, Kobayashi T, Kodama S, et al. Effects of ergocalciferol supplementation on the concentration of vitamin D and its metabolites in human milk. J Nutr. 1989 Nov;119(11):1639–46.

352. Oberhelman SS, Meekins ME, Fischer PR, Lee BR, Singh RJ, Cha SS, et al. Maternal vitamin D supplementation to improve the vitamin D status of breast-fed infants: a randomized controlled trial. Mayo Clin Proc. 2013 Dec;88(12):1378–87.

353. Singh RJ. Quantitation of 25-OH-vitamin D (25OHD) using liquid tandem mass spectrometry (LC-MS-MS). Methods Mol Biol. 2010;603:509–17.

354. Oberson JM, Bénet S, Redeuil K, Campos-Giménez E. Quantitative analysis of vitamin D and its main metabolites in human milk by supercritical fluid chromatography coupled to tandem mass spectrometry. Anal Bioanal Chem. 2020 Jan;412(2):365–75.

355. Olafsdottir AS, Wagner KH, Thorsdottir I, Elmadfa I. Fat-soluble vitamins in the maternal diet, influence of cod liver oil supplementation and impact of the maternal diet on human milk composition. Ann Nutr Metab. 2001;45(6):265–72.

356. Panagos PG, Vishwanathan R, Penfield-Cyr A, Matthan NR, Shivappa N, Wirth MD, et al. Breastmilk from obese mothers has pro-inflammatory properties and decreased neuroprotective factors. J Perinatol. 2016 Apr;36(4):284–90.

357. Hart GR, Furniss JL, Laurie D, Durham SK. Measurement of vitamin D status: background, clinical use, and methodologies. Clin Lab. 2006;52(7–8):335–43.

358. Saadi HF, Dawodu A, Afandi B, Zayed R, Benedict S, Nagelkerke N, et al. Effect of combined maternal and infant vitamin D supplementation on vitamin D status of exclusively breastfed infants. Matern Child Nutr. 2009 Jan;5(1):25–32.

359. Sakurai T, Furukawa M, Asoh M, Kanno T, Kojima T, Yonekubo A. Fat-soluble and water-soluble vitamin contents of breast milk from Japanese women. J Nutr Sci Vitaminol (Tokyo). 2005 Aug;51(4):239–47.

360. Hasegawa H, Arima H, Suzuki Y, Isshiki H, Iida K, Yamamoto Y. Determination methods of vitamins in the neutral and acidic liquid preparations for enteral alimentation, in which changes in vitamin contents occurred during storage [Japanese]. Vitamins. 1992;66:513–26.

361. Blanco D, Fernández MP, Gutiérrez MD. Simultaneous determination of fat-soluble vitamins and provitamins in dairy products by liquid chromatography with a narrow-bore column. Analyst. 2000 Mar;125(3):427–31.

362. Specker BL, Tsang RC, Hollis BW. Effect of race and diet on human-milk vitamin D and 25-hydroxyvitamin D. Am J Dis Child. 1985 Nov;139(11):1134–7.

363. Stoutjesdijk E, Schaafsma A, Nhien NV, Khor GL, Kema IP, Hollis BW, et al. Milk vitamin D in relation to the “adequate intake” for 0-6-month-old infants: a study in lactating women with different cultural backgrounds, living at different latitudes. Br J Nutr. 2017 Nov;118(10):804–12.

364. Stoutjesdijk E, Schaafsma A, Kema IP, van der Molen J, Dijck-Brouwer DAJ, Muskiet FAJ. Influence of daily 10-85 μg vitamin D supplements during pregnancy and lactation on maternal vitamin D status and mature milk antirachitic activity. Br J Nutr. 2019 Feb;121(4):426–38.

365. við Streym S, Højskov CS, Møller UK, Heickendorff L, Vestergaard P, Mosekilde L, et al. Vitamin D content in human breast milk: a 9-mo follow-up study. Am J Clin Nutr. 2016 Jan;103(1):107–14.

366. Takeuchi A, Okano T, Tsugawa N, Katayama M, Mimura Y, Kobayashi T, et al. The determination of vitamin D and its metabolites in human breast and cow’s milk. J Micronutrient Anal. 1988;4:193–208.

367. Tsugawa N, Nishino M, Kuwabara A, Ogasawara H, Kamao M, Kobayashi S, et al. Comparison of Vitamin D and 25-Hydroxyvitamin D Concentrations in Human Breast Milk between 1989 and 2016-2017. Nutrients. 2021 Feb 9;13(2):573.

368. Van Zoeren-Grobben D, Schrijver J, Van den Berg H, Berger HM. Human milk vitamin content after pasteurisation, storage, or tube feeding. Arch Dis Child. 1987 Feb;62(2):161–5.

369. Edelstein S, Charman M, Lawson DEM, Kodicek E. Competitive Protein-Binding Assay for 25-Hydroxycholecalciferol. Clinical Science. 1974 Feb 1;46(2):231–40.

370. Wagner CL, Hulsey TC, Fanning D, Ebeling M, Hollis BW. High-dose vitamin D3 supplementation in a cohort of breastfeeding mothers and their infants: a 6-month follow-up pilot study. Breastfeed Med. 2006;1(2):59–70.

371. Wang LC, Chiang BL, Huang YM, Shen PT, Huang HY, Lin BF. Lower vitamin D levels in the breast milk is associated with atopic dermatitis in early infancy. Pediatr Allergy Immunol. 2020 Apr;31(3):258–64.

372. Weisman Y, Bawnik JC, Eisenberg Z, Spirer Z. Vitamin D metabolites in human milk. J Pediatr. 1982 May;100(5):745–8.

373. Uslu Yuvaci H, Yazar H, Köse E, Çoban BN, Aslan MM, Yazici E, et al. Evaluation of the Relationship Between the Level of Vitamin D in Maternal Blood and Breast Milk and Postpartum Depression. J Clin Obstet Gynecol. 2020;30(2):58–64.

1. Data on volume is also reported in (4) and therefore was not extracted from this report. [↑](#footnote-ref-1)
2. Data from this study was also reported in (27–29) and these reports were therefore redundant. [↑](#footnote-ref-2)
3. Data from this study was also reported in (62,63), and therefore the latter were considered rendundant. [↑](#footnote-ref-3)
4. Data from this study was also reported in (82), and therefore the latter was considered rendundant. [↑](#footnote-ref-4)
5. Data from this study was also reported in (95), and therefore the latter was considered rendundant. [↑](#footnote-ref-5)
6. Data from this study was also reported in (103), and therefore the latter was considered rendundant. [↑](#footnote-ref-6)
7. Data from this study was also reported in (127,128), and therefore the latter was considered rendundant. Data from (128) was only taken for the calcium supplementation sub-group analysis. [↑](#footnote-ref-7)
8. Data from this study was also reported in (154), and therefore the latter was considered rendundant. [↑](#footnote-ref-8)
9. Data from this study was also reported in (169), and the latter study was therefore considered redundant. [↑](#footnote-ref-9)
10. Data from this study was also reported in (184), and therefore the latter was considered rendundant. [↑](#footnote-ref-10)
11. Data from this study was also reported in (27–29) and these reports were therefore redundant. [↑](#footnote-ref-11)
12. Data from this study was also reported in (62,63), and therefore the latter were considered rendundant. [↑](#footnote-ref-12)
13. Data from this study was also reported in OBrien 2007, and therefore the latter was considered rendundant. [↑](#footnote-ref-13)
14. Data from this study was also reported in (103), and therefore the latter was considered rendundant. [↑](#footnote-ref-14)
15. Data from this study was also reported in (285), and therefore the latter was considered rendundant. [↑](#footnote-ref-15)
16. Data from this study was also reported in (288), and therefore the latter was considered rendundant. [↑](#footnote-ref-16)
17. Data from this study was also reported in (154), and therefore the latter was considered rendundant. [↑](#footnote-ref-17)
18. Data from this study was also reported in (316), and therefore the latter was considered rendundant. [↑](#footnote-ref-18)
